# Exploring novel thiazolo[5,4-f]quinoline-based scaffolds as promising antimicrobial agents through synthesis and molecular insights

**DOI:** 10.1038/s41598-025-16561-w

**Published:** 2025-08-26

**Authors:** Nanees N. Soliman, Ahmed A. Fadda, Hatem E. Gaffer, Nesma M. Bayoumy

**Affiliations:** 1 Department of Chemistry, Faculty of Science, Mansoura University, Mansoura, 35516 Egypt; 2 Department of Dyeing, Printing, and Auxiliaries, Textile Institute, National Research Centre, Giza, Cairo Egypt; 3 Basic Science Department, Delta University for Science and Technology, Mansoura, Egypt

**Keywords:** Thiazole, Quinoline, Pyridine, Thiophene, Antimicrobial evaluation, Docking study, Chemical biology, Chemistry

## Abstract

In pursuit of novel antimicrobial agents, a series of thiazolo[5,4-f]quinoline derivatives incorporating pyridine and thiophene moieties were synthesized and structurally characterized using IR and ^1^H-NMR spectroscopy. The synthetic strategy employed diverse condensation and cyclization reactions involving cyanoacetamide intermediates, aryl isothiocyanates, and α-halocarbonyl compounds. The resulting compounds were evaluated for their antimicrobial efficacy against *Staphylococcus aureus*, *Escherichia coli*, and *Candida albicans*. Notably, compounds **18**, **19**, and **23** exhibited potent broad-spectrum activity, surpassing standard drugs such as chloramphenicol and cephalothin in several assays (MIC = 3.125–6.25 µg/mL). Structure-activity relationship (SAR) analysis revealed that the incorporation of bulky, lipophilic aromatic systems and electron-withdrawing substituents significantly enhanced antimicrobial potency. Molecular docking studies further supported these findings, with compound **19** demonstrating the strongest binding affinity (-6.6951 kcal/mol) to bacterial DNA gyrase (PDB: 6F86) via a synergistic network of hydrogen bonds and π-interactions. These results highlight the thiazoloquinoline framework, particularly derivatives bearing thiophene and naphthoyl functionalities, as a promising scaffold for future antimicrobial drug development.

**Supplementary Information:**

The online version contains supplementary material available at 10.1038/s41598-025-16561-w.

## Introduction

The global rise in microbial infections and the rapid emergence of multidrug-resistant (MDR) pathogens have created an urgent need for the development of novel antimicrobial agents^1,2^. The limitations of existing antibiotics, in terms of both resistance and efficacy, underscore the importance of identifying new chemotypes with distinct mechanisms of action^3^. Medicinal chemistry plays a pivotal role in addressing this challenge by designing and synthesizing novel compounds with potential therapeutic benefits.

Heterocyclic scaffolds, particularly thiazoles, have garnered significant interest due to their broad spectrum of biological activities. Among them, **2-aminothiazole** is a well-established pharmacophore with demonstrated antiviral^4^, antibacterial^5^, antiprion^6^, anticancer^7^, and neuroactive^8^ properties. Furthermore, 2-aminothiazole derivatives have been explored as adenosine receptor antagonists^9^, anti-inflammatory agents^10^, antidiabetic drugs^11^, antituberculosis^12^ anthelmintic agents^13^, and different other biological activity^14–16^.

The **pyridine** nucleus is another key heterocyclic moiety in drug design, with numerous derivatives showing potent antibacterial, antifungal, and antiviral activities^17–19^. Fusion of pyridine with other heterocycles often enhances both potency and pharmacokinetic profiles^21,22^. Specific substituents such as amino, hydroxyl, methoxy, sulfamide, and hydrazide groups have been shown to further augment biological activity^20^.

Similarly, **thiophene** derivatives are widely recognized for their pharmacological potential, particularly in antimicrobial drug development^23–25^. Thiophene’s electron-rich, planar structure facilitates strong interactions with microbial targets and has been frequently incorporated into therapeutic agents^24,25^.

**Aryl isothiocyanates**, characterized by the reactive –N = C = S group, are versatile intermediates in heterocyclic synthesis and contribute to diverse biological functions, including antimicrobial, antifungal, and antiprotozoal effects^26–28^. The utility of **cyanoacetamide derivatives** as multifunctional precursors has also been documented in the construction of five- and six-membered heterocycles such as pyrazoles, pyridines, pyrimidines, and fused polyheterocycles^29–37^.

Given the wide-ranging bioactivities of thiazole, pyridine, and thiophene scaffolds, this study focuses on the synthesis of novel **thiazolo[5**,**4-f]quinoline** derivatives incorporating pyridine and thiophene moieties. These compounds were designed based on the 2-aminothiazoloquinoline core and synthesized via condensation and cyclization reactions involving cyanoacetamides and aryl isothiocyanates. Their antimicrobial properties were systematically evaluated to identify promising leads against *Staphylococcus aureus*, *Escherichia coli*, and *Candida albicans*. The current work aims to provide both synthetic insights and biological evaluation of this novel scaffold as a potential foundation for future antimicrobial development.

**Fig. 1:**
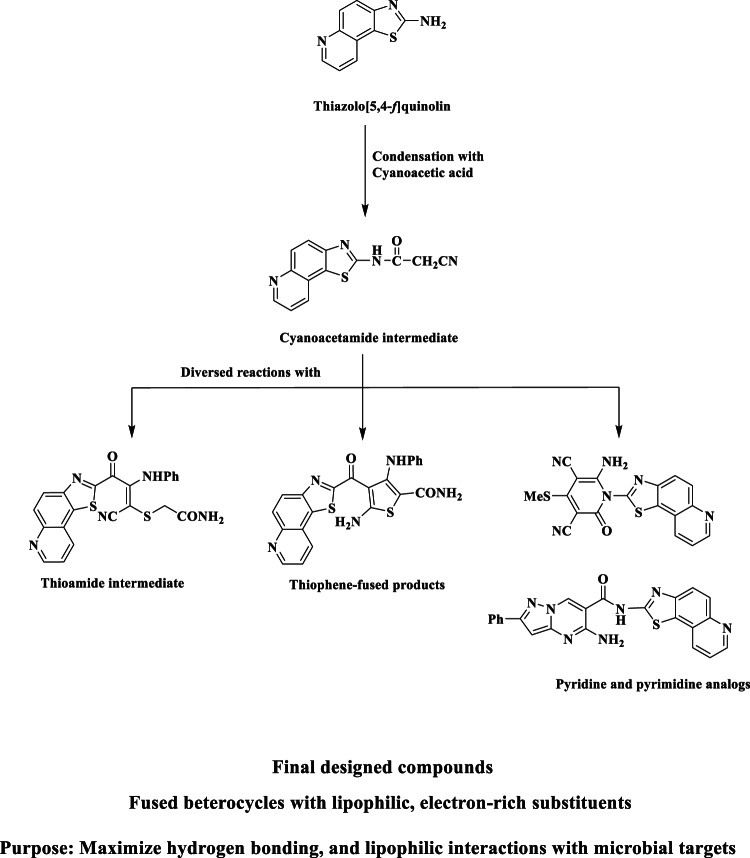
Rational design strategy for thiazolo[5,4-f]qulinnoline-based antimicrobial agents.

In light of the extensive bioactivity exhibited by thiazole, pyridine, and thiophene derivatives, we rationally designed a new series of **thiazolo[5**,**4-f]quinoline-based compounds** by integrating these pharmacophores into a unified scaffold. The choice of the **thiazolo[5**,**4-f]quinoline** core was driven by its rigid, planar geometry, which is conducive to **π–π stacking** and **DNA intercalation**, potentially enhancing antimicrobial activity. The design strategy is illustrated in Fig. 1.

## Results and discussion

### Chemistry

In the course of our studies on the synthesis of antimicrobial agents our interest was focused on thiazolequinoline derivatives. Thiazolo[5,4-f] and [4,5-f] quinolines were reported by Dohmori and Nagano^38,39^, respectively.

**Fig. 2:**
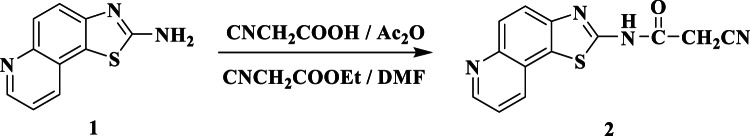
Synthesis of cyano acetamide derivative **2**.

The present work is concerned with the synthesis of thiazolo quinoline derivatives possessing pyridine and thiophene ring systems and their antimicrobial activities. The starting material 2-cyano-N-(thiazolo[5,4-f]quinolin-2-yl)acetamide **(2)** has been prepared by refluxing 2-aminothiazoloquinoline **(1)** with cyanoacetic acid and acetic anhydride or refluxing compound **1** with ethyl cyanoacetate in DMF (Fig. 2). Structure of compound **2** was established by elemental and spectroscopic analyses. The IR spectrum showed absorption bands at *ν* 3230 (NH), 2220 (CN), 1675 (CO), and 1601 cm^− 1^ (C = N), while its ^1^H-NMR spectrum revealed singlet signal at δ 3.21 ppm due to CH_2_ protons.

**Fig. 3:**
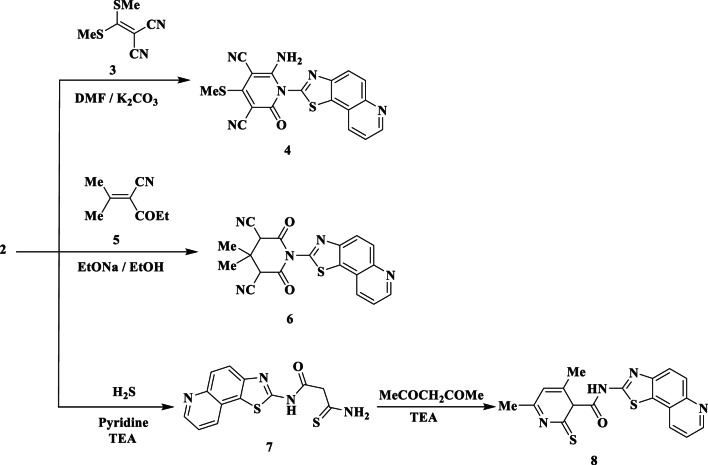
Synthesis of some new pyridine derivatives **4–8**.

A crucial component of the chemical structures of many pharmaceutically approved medications is the pyridine scaffold. Thus, cyanoacetamide **2** and ketene dithioacetal **3** were refluxed in DMF with anhydrous potassium carbonate acting as a catalyst to give dihydropyridine-3,5-dicarbonitrile **(4)** (Fig. 3). The NH_2_, 2 CN, and amidic CO groups were responsible for the absorption bands at *v* 3420, 3336, 2220, 2200, and 1680 cm^− 1^ in the IR spectrum of **4**. S-Me and NH_2_ protons were identified by the ^1^H-NMR as the source of two distinctive singlet signals at δ 2.80 and 6.56 ppm.

Furthermore, in the presence of sodium ethoxide, **2** interacts with ethyl-3-cyano-2-methyl-but-2-enoate **(5)** to produce the pyridine derivative **6** (Fig. 3). The IR spectrum of **6** showed the absorption bands of the cyano and amidic carbonyl groups appeared at *ν* 2219 and 1670 cm^− 1^, respectively, while the ^1^H-NMR displayed singlet signal at δ 0.91 ppm for the methyl protons. 3-Amino-N-(thiazolo[5,4-f]quinolin-2-yl)-3-thioxopropanamide **(7)** was achieved by reacting compound **2** with hydrogen sulfide in refluxing pyridine and a catalytic amount of TEA as a base (Fig. 3). The IR spectrum of **7** showed absorption bands at *ν* 3410, 3230, 1682, and 1150 cm^− 1^ referred to NH_2_, NH, CO, and C = S function groups, respectively. Two singlet signals at δ 3.73 and 6.73 ppm were appeared in its ^1^H-NMR due to CH_2_ and NH_2_ groups, respectively.

Then, using TEA as a catalyst, compound **7** was exposed to a reaction with an acetyl acetone in boiling ethanol, yielding pyridine-2-thione derivative **8** (Fig. 3). The NH_2_ group disappeared, as seen in the IR spectra of **8**. C_4_-CH_3_, C_6_-CH_3_, C_3_-H pyridine, C_5_-H pyridine, and NH protons were represented by singlet signals at δ 1.82, 2.33, 3.12, 5.95, and 12.66 ppm in the ^1^H-NMR spectrum, respectively.

**Fig. 4:**
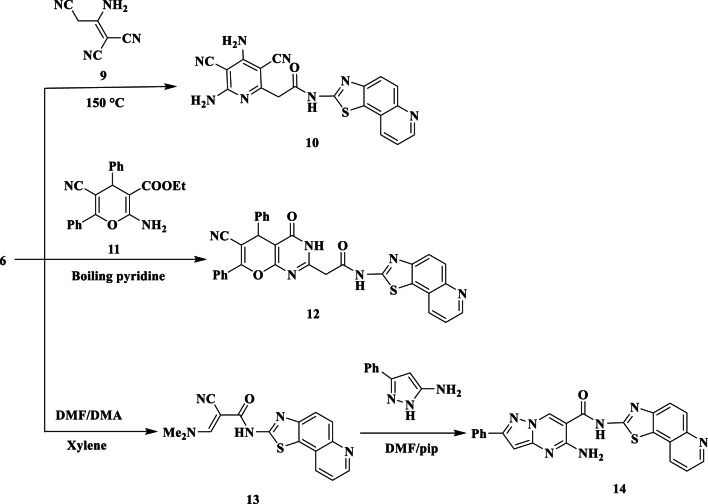
Synthesis of some new pyridine derivatives **10** and **12** and pyrazolopyrimidine derivative **14**.

Furthermore, it was discovered that the pyridine derivative **10** is created when **2** and malononitrile dimer are heated in a pressure tube in a silicon oil bath (Fig. 4). Heating **2** with two moles of malononitrile in refluxing ethanol with a catalytic quantity of piperidine produced a similar result (Fig. 4). NH_2_, NH, CN, CO, and C = N function groups were represented by the absorption bands at *v* 3450, 3235, 2219, 1680, and 1618 cm^− 1^ in the IR spectrum of **10**, whereas the NH_2_ and NH protons were responsible for the distinctive two singlet signals at δ 6.44 and 12.66 ppm in the ^1^H-NMR spectrum.

Dihydropyrano-[2,3-d]primidinone **(12)** is produced when cyanoacetamide **2** combines with 2-amino-3-carboxy-5-cyano-4,6-diphenyl-4 H-pyran **(11)** in boiling pyridine (Fig. 4). Furthermore, **2** reacts with DMF/DMA in refluxing dry xylene to produce enaminonitrile **13** (Fig. 4). The methyl, olefinic-CH, and NH protons, respectively, produced three singlet signals at δ 3.09, 7.50, and 12.76 ppm in the ^1^H-NMR spectrum of **13**.

Compound **13** was subjected to react with 2-aminopyrazole in boiling DMF catalyzed by drops of piperidine afforded pyrazolopyrimidine derivative **14** (Fig. 4). The IR spectra of **14** showed absorption bands at *ν* 3420, 3320, and 1710 cm^− 1^, respectively, due to the NH_2_, NH, and amidic CO groups. At δ 6.57, 6.80, 8.90, and 12.70 ppm, the ^1^H-NMR of **14** displayed characteristic singlet signals due to pyrazole-CH, NH_2_, pyrimidine-CH, and NH.

The synthesis of thiazole, pyrazole, oxazine, and pyrimidine ring systems has been made easier by research into the reaction between phenyl isothiocyanate and active methylene compounds in alkaline circumstances. With phenyl isothiocyanate as a key starting material, our recent synthetic efforts have expanded to include the synthesis of hitherto unavailable heterocyclic ring systems. When several substrates with the N = C = S moiety interact with α-halocarbonyl compounds, they undergo cyclization and produce thiazoles, 2,3-dihydrothiazoles, and thiazolidines, which have been shown to have fungicidal, antiprotozoal, and local anesthetic effects^40^.

**Fig. 5:**
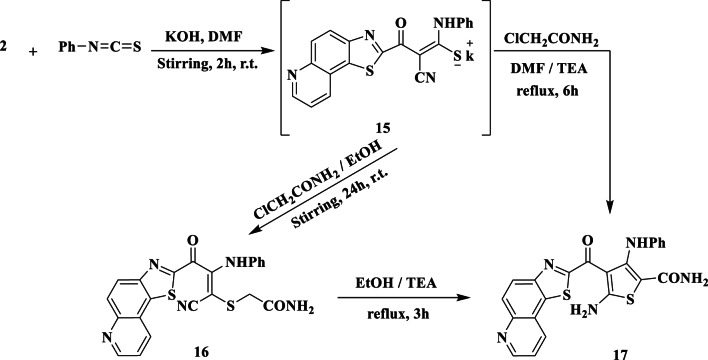
Synthesis of thiocarbamoyl potassium salt **15** and its reaction with α-haloketone.

We present an expansion of the synthetic methodology originally described by Hantzsch and Weber^41^, which demonstrates broad applicability. In this extended approach, the non-isolable intermediate **15** is generated through the base-induced reaction between the acidic methylene compound **2** and phenyl isothiocyanate in dry DMF at ambient temperature (Fig. 5). Subsequent treatment of compound **15** with α-chloroacetamide in boiling DMF, catalyzed by TEA drops, yielded product **17** (Fig. 5). Accurate analysis of this product confirmed its composition as C_22_H_15_N_5_O_2_S_2_, and its spectral data provided insights leading to the determination of structure 17. The IR spectrum exhibited absorption peaks at ν 3450, 3155, 1705, and 1661 cm^− 1^, corresponding to NH_2_, NH, C = O, and CONH_2_ functionalities, respectively, while the CN stretching band was disappeared. Analysis of the ^1^H-NMR spectrum revealed a broad singlet at δ 6.68 ppm attributed to CO-NH_2_, multiplet signals integrated for (9 H) appeared at δ 7.19–7.58 attributed to aromatic protons, NH_2_ and pyridine-C_3_H protons, and a singlet (NH) at 10.49 ppm. Upon agitation of the compound with D_2_O, the broad band signals at δ 6.68, 7.29, and 10.49 ppm disappeared. These observations supported the assignment of structure 17 to this product. Additionally, the structure of **17** was validated through an alternative synthesis route. Treatment of intermediate **15** with α-chloroacetamide in refluxing ethanol resulted in the formation of the acyclic compound **16** (Fig. 5). Based on elemental and spectral analyses, structure 16 was proposed for the reaction product. The IR spectrum of **16** displayed a cyano absorption band at ν 2220 cm^− 1^, along with NH_2_, NH, CO, and C = O (amidic) functionalities observed at ν 3340, 3150, 1700, and 1680 cm^− 1^, respectively. Refluxing **16** in ethanol with a few drops of TEA yielded a product identical in all aspects (melting point, mixed melting point, IR, ^1^H-NMR) to **17**.

**Fig. 6:**
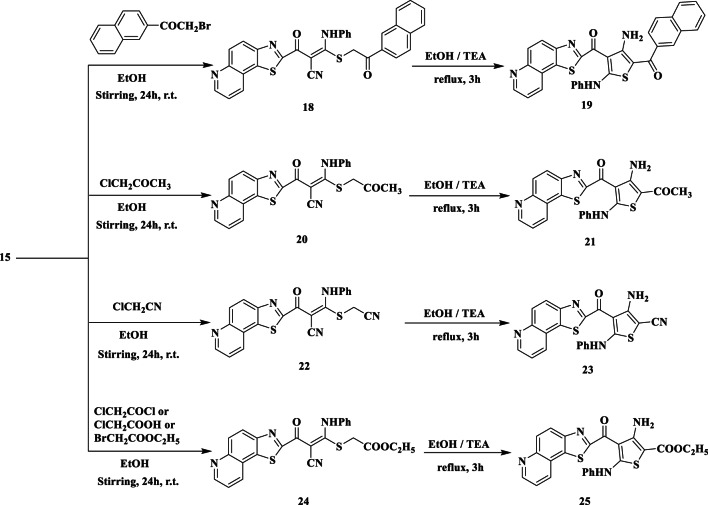
Synthesis of thioacetamide **18**, **20**, **22**, and **24** and thiophene derivatives **19**, **21**, **23**, and **25**.

In boiling ethanol, intermediate **15** also reacts with equimolar amounts of 2-(naphthalen-2-yl) acetyl bromide to produce a yellow-colored product **(18)**. To obtain thiophene derivative **19**, compound **18** was heated controversially for four hours in ethanol and a catalytic quantity of TEA (Fig. 6). Structures 18 and 19 were established as a result of accurate spectral and elemental investigations. The nitrile group was represented by absorption spectra at *v* 2217 cm^− 1^ in compound **18’s** IR spectrum. In contrast, compound **19’s** IR spectrum showed a stretching frequency band at 3450 cm^− 1^, which corresponded to the NH_2_ function, rather than an absorption band in the region at *ν* 2220 cm^− 1^, which was caused by the CN group. The chemical formula C_32_H_20_N_4_O_2_S_2_ (M^+^, 556) was revealed by the mass spectrum of **19**.

By eliminating KCl, intermediate **15** easily reacted with chloroacetone in boiling ethanol to produce the acyclic **20**. The thiophene derivative **21** was produced by refluxing **20** in ethanol with a catalytic amount of TEA (Fig. 6). Spectral data and elementary analysis verified the structure of this compound. An alternative synthesis of **21** further confirmed its structure. Thus, the thiophene derivative **21** can be obtained in a respectably high yield by refluxing intermediate **15** directly with chloroacetone in DMF/TEA (Fig. 6).

Additionally, in refluxing ethanol treated with chloroacetonitrile, the corresponding acyclic **22** is exclusively and well isolated from the intermediate **15** (Fig. 6). Elemental and spectral analyses have been used to confirm the structure of **22**. For example, there are bands in the IR spectrum at ν 3210 (NH), 2220, and 2200 cm^− 1^ (2CN). There is a CH_2_ signal at δ 4.29 ppm in its ^1^H-NMR spectrum. In addition, heating **22** in ethanol with a catalytic quantity of TEA results in the thiophene derivative **23** (Fig. 6). The IR spectrum of thiophene structure 23, which confirmed bands associated with NH_2_, NH, and CN functions, was used to establish the structure. Characteristic broad signals at δ 5.73 and 10.22 ppm due to NH_2_ and NH are revealed in its ^1^H-NMR spectrum. However, it has been found that refluxing **15** and chloroacetonitrile in DMF/TEA directly form it (Fig. 6).

Intermediate **15** underwent treatment with equimolar quantities of chloroacetyl chloride or chloroacetic acid in boiling ethanol, resulting in a favourable yield of a product characterized by the formula C_24_H_18_N_4_O_3_S_2_ (Fig. 6). Its infrared spectrum displayed bands attributable to NH, CN, and CO functionalities, facilitating the determination of the acyclic structure 24. Characteristic signals for *CH*_*3*_CH_2_, CH_2_, and *CH*_*2*_CH_3_ were detected in the ^1^H-NMR spectrum at δ 1.27, 3.99, and 4.22 ppm, respectively. NH signal, exchangeable with D_2_O, at δ 10.66 ppm. On the other hand, when **15** was treated with ethyl bromoacetate in refluxing ethanol, a single product that was identical to **25** in terms of melting point, mixed melting point, and infrared spectrum was formed. It showed the mass spectrum of **25** (M^+^, 474). The equivalent thiophene derivative **25** was obtained by refluxing **24** in ethanol with a catalytic quantity of TEA (Fig. 6).

### Biological activity

The antibacterial and antifungal properties of the synthesized thiazolo[5,4-f]quinoline derivatives were evaluated against *Staphylococcus aureus* (Gram-positive), *Escherichia coli* (Gram-negative), and *Candida albicans* (fungal strain), with the results summarized in Table 1. The biological assays were interpreted in terms of both minimum inhibitory concentration (MIC, µg/mL) and inhibition zone diameter (mm), revealing valuable insights into structure–activity relationships (SAR).

The antibacterial evaluation against *S. aureus* revealed that several derivatives exhibited significant potency. Compounds **18**, **19**, and **23** demonstrated the highest activity, each with a MIC value of 3.125 µg/mL and inhibition zones of 44, 44, and 43 mm, respectively. These results are comparable to, or slightly exceed, the reference drug Chloramphenicol, which had a MIC of 3.125 µg/mL and an inhibition zone of 44 mm. The enhanced activity of these derivatives suggests that specific substitutions particularly aryl ketones and thiophene-based moieties play a critical role in enhancing antibacterial action. Compounds **17**, **21**, and **22** also showed good activity, with MICs of 6.25 µg/mL and inhibition zones of 40, 40, and 39 mm, respectively, indicating that these structures retain considerable potency despite slight modifications in their substitution patterns. Compounds such as **6**, **8**, **10**, **12**, **13**, and **14** showed moderate inhibition (MICs ranging from 12.5 to 25 µg/mL), while others, including **2**, **4**, **7**, **24**, and **25**, displayed poor activity with MIC values between 50 and 100 µg/mL. The declining activity in these compounds may be attributed to the absence of electron-rich or planar substituents, which are likely crucial for optimal interaction with bacterial targets.

Activity against the Gram-negative strain *E. coli* followed a similar trend but was generally lower, consistent with the increased resistance typically exhibited by Gram-negative bacteria due to their outer membrane. Nonetheless, compounds **18**, **19**, and **23** remained highly active, again exhibiting MICs of 3.125 µg/mL and inhibition zones around 43–44 mm. Remarkably, this performance surpassed that of standard antibiotics Cephalothin (6.25 µg/mL, 38 mm) and Chloramphenicol (6.25 µg/mL, 37 mm). These results suggest that the presence of naphthoyl or phenylamino substituents may improve bacterial membrane permeability or binding affinity. Moderate antibacterial effects were observed for compounds **6**, **13**, **14**, **16**, **21**, and **22** (MIC = 25 µg/mL), indicating that subtle differences in electronic or steric features can markedly affect efficacy. In contrast, compounds such as **2**, **4**, **7**, **10**, **24**, and **25** were far less active (MIC ≥ 50 µg/mL), reinforcing the critical influence of specific substitution patterns on activity against Gram-negative organisms.

The antifungal evaluation revealed that compounds **18**, **19**, and **23** also possessed potent activity against *C. albicans*, with MICs of 6.25 µg/mL and inhibition zones of 39–40 mm. While not quite matching the standard antifungal Cycloheximide (3.125 µg/mL, 42 mm), these results are nonetheless impressive and suggest a consistent pharmacophoric trend across both antibacterial and antifungal profiles. The presence of naphthoyl and phenyl-substituted thiophene groups once again appears to enhance biological activity, potentially by increasing lipophilicity and membrane penetration. Compound **17** showed moderate antifungal potential (MIC = 12.5 µg/mL, inhibition zone = 34 mm), while compounds **6**, **12**, **14**, **16**, and **21** exhibited weaker activity with MICs of 50 µg/mL. The remainder of the compounds displayed little to no antifungal effect, with MIC values equal to or exceeding 100 µg/mL, highlighting the specificity of active pharmacophores required to inhibit fungal growth effectively.

**Table 1:** Antimicrobial activity of the newly synthesized compounds.

Compound No.	MIC (µg/mL) and inhibition zone (mm)		
	Gram Positive bacteria	Gram Negative bacteria	Fungi
	*S. aureus*	*E. coli*	*C. albicans*
	ATCC: 6538	ATCC: 9637	ATCC: 10,231
**2**	100 (14)	100 (14)	100 (14)
**4**	50 (17)	100 (15)	100 (14)
**6**	25 (28)	25 (29)	50 (19)
**7**	50 (18)	50 (17)	100 (14)
**8**	25 (27)	50 (18)	100 (14)
**10**	25 (28)	50 (17)	100 (15)
**12**	25 (27)	50 (18)	50 (17)
**13**	25 (27)	25 (27)	50 (19)
**14**	12.5 (35)	25 (28)	50 (18)
**16**	12.5 (34)	25 (26)	50 (17)
**17**	6.25 (40)	25 (28)	12.5 (34)
**18**	3.125 (44)	3.125 (43)	6.25 (39)
**19**	3.125 (44)	3.125 (44)	6.25 (40)
**20**	12.5 (36)	25 (27)	25 (25)
**21**	6.25 (40)	25 (27)	50 (17)
**22**	6.25 (39)	25 (28)	12.5 (35)
**23**	3.125 (43)	3.125 (44)	6.25 (40)
**24**	50 (17)	50 (17)	100 (15)
**25**	50 (17)	50 (16)	100 (14)
**Chloramphenicol**	3.125 (44)	6.25 (37)	NT
**Cephalothin**	6.25 (36)	6.25 (38)	NT
**Cycloheximide**	NT	NT	3.125 (42)

### Structure-Activity relationship (SAR) analysis

A detailed SAR analysis was conducted to elucidate the molecular features responsible for the observed antimicrobial activity of the synthesized thiazolo[5,4-f]quinoline derivatives. Several key structural motifs and substituent effects were identified as critical determinants of biological efficacy.

All compounds in the series possess the thiazolo[5,4-f]quinoline scaffold, which serves as the core pharmacophore. This fused heterocyclic system contributes significantly to biological activity due to its rigid planarity, facilitating potential intercalation into microbial DNA or interaction with enzymatic active sites. The electron-rich sulfur and nitrogen atoms within the fused ring system may further engage in hydrogen bonding or dipole–dipole interactions with microbial targets. In addition, the extended π-system may promote hydrophobic and π–π stacking interactions with membrane components or protein residues, enhancing the general antimicrobial potential of the scaffold.

A key observation from the activity profiles is the superior potency of compounds bearing bulky aromatic moieties, particularly naphthoyl or phenyl rings. Compounds **18**, **19**, and **23** consistently among the most active against *S. aureus*, *E. coli*, and *C. albicans*—share the presence of such hydrophobic groups. These aromatic substituents likely increase the compounds’ lipophilicity, thereby improving membrane permeability, which is particularly advantageous when traversing the outer membrane of Gram-negative bacteria or penetrating fungal cell walls.

Compound **18**, for instance, incorporates a naphthalen-2-yl group and a thiolated acrylonitrile linkage, a combination that appears to maximize both lipophilic and electronic interactions. Similarly, compound **19** contains a naphthoyl moiety paired with a phenylamino-substituted thiophene, promoting both aromatic stacking and electron-donating character. Compound **23** also benefits from a thiophene-phenylamino system, suggesting that the interplay of hydrophobicity and hydrogen-bonding capacity significantly boosts its bioactivity.

The incorporation of electron-withdrawing groups such as cyano (–CN), keto (C = O), and ester (–COOEt) functionalities appears to contribute positively to activity. These groups, as seen in compounds like **18**, **20**, **22**, and **24**, may increase the electrophilicity of the molecule, thereby enhancing reactivity toward nucleophilic residues in microbial enzymes or facilitating covalent interactions with biological targets. When these groups are conjugated with electron-rich systems such as phenylamino or thiophene moieties the electronic polarization likely results in improved binding affinity. However, the orientation and position of these groups are crucial; overly electron-deficient systems may compromise solubility or alter binding geometry unfavorably.

Thiophene-containing derivatives, including compounds **17**, **19**, **21**, **23**, and **25**, exhibited superior antibacterial and antifungal profiles. The inclusion of the thiophene ring a known bioisostere of phenyl groups contributes both electronic richness and planarity, aiding in target engagement and membrane interaction. Particularly in Gram-positive bacteria and fungi, the thiophene substituents appear to support effective hydrogen bonding and hydrophobic contact within the cellular environment. The versatility of thiophene in medicinal chemistry is further supported by its ability to modulate pharmacokinetic properties, possibly enhancing compound stability and bioavailability.

Several moderately active compounds (e.g., **4**, **8**, **10**, **12**) contain free amino or amide functionalities. These groups likely contribute to hydrogen bonding interactions with microbial targets, which can facilitate binding. However, excessive polarity introduced by multiple hydrogen-bond donors may hinder membrane permeation, especially in Gram-negative organisms. Thus, a delicate balance between hydrophilic and lipophilic character is necessary for optimizing antimicrobial efficacy. While these polar groups aid in target specificity, their positioning and surrounding structural context must be carefully optimized to avoid compromising cellular uptake.

Compounds such as **10** and **12** incorporate extended ring systems like substituted dicyanopyridines and pyrano[2,3-d]pyrimidines. These systems introduce rigidity and planarity, which may benefit DNA intercalation or protein binding but can also hinder membrane penetration due to steric bulk. While these compounds demonstrated moderate activity, they were not among the most potent, suggesting that excessive steric hindrance or inflexibility may interfere with optimal molecular recognition or transport across biological barriers. Incorporating flexible linkers or solubilizing groups may be necessary to improve the pharmacokinetic profile of such extended systems.

### Statistical analysis and interpretation of antimicrobial data

1. Statistical analysis.

Table 2 presents the summary statistics for the Minimum Inhibitory Concentration (MIC) values of the tested antimicrobial agent against three different microorganisms: *Staphylococcus aureus*, *Escherichia coli*, and *Candida albicans*. The MIC represents the lowest concentration of the antimicrobial required to inhibit the visible growth of the organism.

**Table 2:** Summary statistics (MIC µg/mL).

Organism	Mean MIC	Min MIC	Max MIC	Median MIC	Std. Dev.
*S. aureus*	25.82	3.125	100.0	25.0	24.80
*E. coli*	37.34	3.125	100.0	25.0	27.43
*C. albicans*	56.25	6.25	100.0	50.0	37.96

2. Interpretation of summary statistics.

- **Mean MIC**:
  - *C. albicans* has the **highest mean MIC (56.25 µg/mL)**, indicating it is the **most resistant** organism to the tested compounds.
  - *S. aureus* shows the **lowest average MIC (25.82 µg/mL)**, suggesting better susceptibility.
  - *E. coli* is moderately susceptible with a mean MIC of 37.34 µg/mL.
- **Median MIC**:
  - Both *S. aureus* and *E. coli* have a **median MIC of 25 µg/mL**, while *C. albicans* has a **higher median of 50 µg/mL**.
- **Minimum MIC**:
  - All three organisms had compounds with **very low MICs (as low as 3.125 µg/mL for** ***S. aureus*** **and** ***E. coli***, **6.25 µg/mL for** ***C. albicans*)**, showing that certain compounds (e.g., 18, 19, 23) are **broad-spectrum and highly potent**.
- **Standard Deviation**:
  - *C. albicans* exhibits the **highest standard deviation (37.96)**, reflecting greater variability in sensitivity across compounds.
  - This suggests that some compounds are very effective against fungi, while others are almost inactive.

3. ANOVA Statistical Test Results.

- **F-statistic**: 4.79.
- **p-value**: 0.0122.

4. ANOVA Interpretation.

The **One-Way ANOVA test** was used to assess whether there are statistically significant differences in the **mean MIC values among the three organisms**.

- The **p-value < 0.05** confirms a **significant difference** exists in MIC means across the groups.
- This result supports the observation that **different organisms respond differently** to the same set of compounds.

5. Overall Discussion and Implications.

- The statistical analysis clearly shows that:
  - *C. albicans* is generally more **resistant** to the tested compounds.
  - *S. aureus* is the most **sensitive**, suggesting these compounds may be more promising as **Gram-positive antibacterial agents**.
  - There is considerable variability in compound performance, especially with antifungal activity, suggesting a strong **structure-activity relationship (SAR)**.
- **Statistical significance** (confirmed by ANOVA) validates that these differences are not due to random chance but reflect **true biological variation** in response.

### Molecular docking

The protein structure **PDB: 6F86**, which represents **bacterial DNA gyrase subunit B**, was selected as the docking target because DNA gyrase is a **well-established and clinically validated target** for antibacterial drug development. It plays a crucial role in regulating DNA supercoiling and replication in bacteria. Inhibiting this enzyme can effectively block bacterial proliferation. The crystal structure 6F86 offers high-resolution coordinates and contains the **ATP-binding site** essential for activity, making it an ideal candidate for assessing the binding affinity of our designed thiazolo[5,4-f]quinoline derivatives. Moreover, previous studies have reported successful docking of other antimicrobial scaffolds to this structure, further supporting its relevance for our study.

**Fig. 7:**
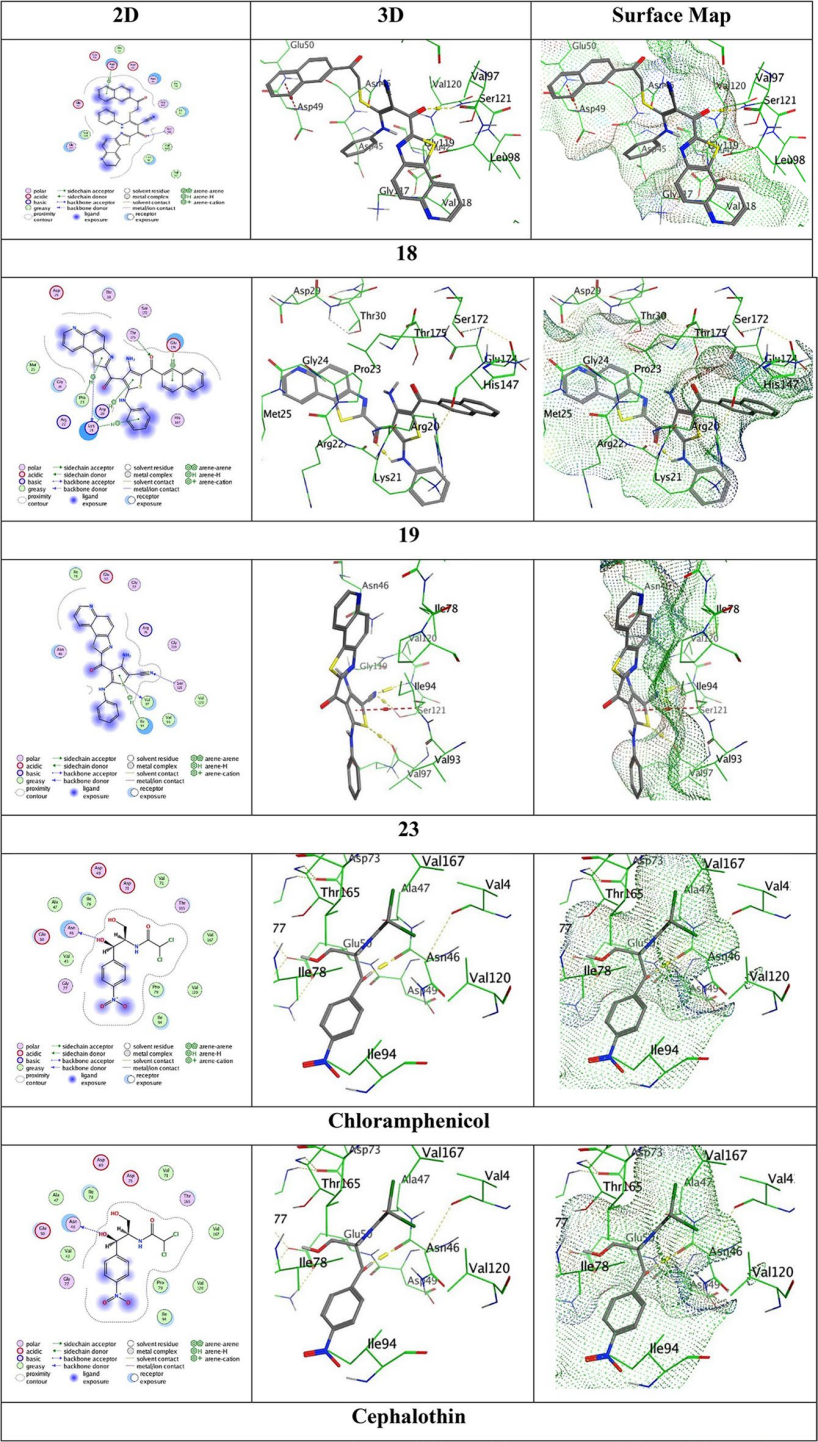
The molecular docking images between the ligands and PDB: with 6F86.

The molecular docking results revealed significant interactions between the selected compounds **18**, **19**, and **23** with potent antimicrobial activities and the target protein (PDB: 6F86) (Table 3 and Fig. 7). Compound **18** established a dual binding mode in the gyrase active site with binding energy − 6.5988 kcal/mol (RMSD 1.8016). The ketonic oxygen formed a hydrogen acceptor bond with Ser121 at a distance 3.46 Å, indicative of moderate electrostatic interaction strength. Simultaneously, its naphthalene ring engaged in π-H stacking with Asp49 at a distance 3.80 Å, characteristic of favourable van der Waals bindings. This combination of polar and hydrophobic bonds suggests a balanced binding, though the relatively high RMSD implies some conformational flexibility during the binding. However, compound **19** emerged as the most promising candidate with the lowest binding energy (-6.6951 kcal/mol) and stable geometry (RMSD 1.7715). Its binding network featured: (1) a sulfur-atom of the thiophene-ring to-Lys21 hydrogen donor bond (3.68 Å), (2) a critical carbonyl oxygen acceptor interaction with Thr175 at 3.06 Å, the shortest and likely strongest bond length in the series, and (3) four π-H interactions involving both aromatic systems (thiazole and aniline rings) with Lys21 (4.03, 4.36 Å), Pro23 (4.32 Å), and Glu174 (3.63 Å), respectively. The intermolecular distance 3.63 Å contact with Glu174 represents the tightest π-H interaction observed, approaching the optimal range for such bonds (3.5-4.0 Å). This multi-point attachment strategy, combining hydrogen bonding and π-stacking, creates a synergistic binding effect that explains its superior affinity over reference antibiotics. Meanwhile, **compound 23** displayed the weakest binding (-5.2126 kcal/mol) but most precise orientation (RMSD 1.1328). Its binding relied on: (1) thiophene sulfur coordination with Val97 (3.76 Å), (2) nitrile nitrogen accepting a hydrogen bond from Ser121 (3.41 Å), and (3) thiophene π-stacking with Ile94 (4.40 Å). While the 3.41 Å Ser121 interaction is relatively strong, the 4.40 Å π-contact exceeds optimal distances for effective stacking, likely contributing to the reduced binding energy. However, the exceptionally low RMSD suggests this scaffold may serve as a rigid core for further optimization through substituent additions to enhance interactions with adjacent residues.

Comparative analysis with clinical references reveals that while chloramphenicol (-5.3271 kcal/mol) forms only one hydrogen bond with Asn46 (2.73 Å), and cephalothin (-6.4089 kcal/mol) establishes two hydrogen bonds (Thr165:3.97 Å, Gly77:3.42 Å), compound **19** achieves stronger binding through a diversified interaction portfolio.

**Table 3:** Molecular Docking results between the potent compounds and PDB: 6F86.

No	Binding energy	RMSD	Ligands and amino acids interaction	Bond type	Distances (A°)
**18**	-6.5988	1.8016	O 2 of the ketonic group with Ser121 Naphthalene- ring with Asp49	H-acceptor pi-H	3.46 3.80
**19**	-6.6951	1.7715	S 31 of the thiophene-ring with Lys21 O 10 of the ketonic group with Thr175 Thiazole-ring with Lys21 Aniline-ring with Lys21 Thiazole-ring with Pro23 Aniline-ring with Glu174	H-donor H-acceptor pi-H pi-H pi-H pi-H	3.68 3.06 4.36 4.03 4.32 3.63
**23**	-5.2126	1.1328	S 5 of the thiophene-ring with Val97 N 30 of the nitrile-group with Ser121 The thiophene- ring with Ile94	H-donor H-acceptor pi-H	3.76 3.41 4.40
**Chloramphenicol**	-5.3271	1.9257	O 20 of the hydroxyl group with Asn46	H-donor	2.73
**Cephalothin**	-6.4089	1.6526	S 8 of the thiazine-ring with Thr165 N 14 of the amid-group with Gly77	H-donor H-donor	3.97 3.42

## Experiment

### Synthesis of 2-cyano-N-(thiazolo[5,4-f]quinolin-2-yl)acetamide (2)

Compound **1** (2.00 g, 0.01 mol) and cyanoacetic acid (0.85 g, 0.01 mol) were refluxed for 6 h (15 mL) in the presence of acetic anhydride (5 mL). Once the reaction mixture had cooled to room temperature, 150 mL of ice-cold water was added. The solid product was filtered and recrystallized from ethanol to give compound **2**.

Yield, 81%; m.p. 260–262 °C; IR (KBr): *ν*_*max*_, cm^− 1^: 3230 (NH), 2220 (CN), 1675 (CO), 1601 (C = N) (Figure S1); ^1^H-NMR (DMSO-*d*_*6*_) *δ* ppm: 3.21 (s, 2 H, CH_2_), 7.51 (m, 1H, pyridine-C_3_H), 7.62 (d, 1H, Ar-H), 8.04 (d, 1H, Ar-H), 8.45 (d, 1H, pyridine-C_4_H), 8.96 (d, 1H, pyridine-C_2_H), 12.33 (s, 1H, NH) (Figure S2); ^13^C-NMR (DMSO-*d*_*6*_) *δ* ppm: 24.8, 120.5, 123.5, 124.0, 125.7, 126.4, 129.4, 131.5, 135.9, 144.0, 148.8, 169.0, 174.1 (Figure S3). MS (m/z, %): 268 (M^+^, 56) (Figure S4). Anal. Calced for C_13_H_8_N_4_OS (268.29): C, 58.20; H, 3.01; N, 20.88%. Found: C, 58.15; H, 2.96; N, 20.82%.

### Synthesis of 6-amino-4-(methylthio)-2-oxo-1-(thiazolo[5,4-f]quinolin-2-yl)-1,2-dihydropyridine-3,5-dicarbonitrile (4)

Compound **2** (2.60 g, 0.01 mol) and 2-(bis(methylthio)methylene malonitrile **(3)** (1.70 g, 0.01 mol) were refluxed for 4 h in dry DMF (15 mL) in the presence of anhydrous potassium carbonate (0.50 g). The solid material was removed from ethanol, dried, and then recrystallized from ethanol to obtain the desired product **4**.

Yield, 73%; m.p. 221–223 °C; IR (KBr): *ν*_*max*_, cm^− 1^: 3420, 3336 (NH_2_), 2220 (CN), 2200 (CN), 1680 (amidic CO), 1642 (C = N) (Figure S5); ^1^H-NMR (DMSO-*d*_*6*_) *δ* ppm: 2.80 (s, 3 H, CH_3_), 6.55 (s, 2 H, NH_2_), 7.35 (m, 1H, pyridine-C_3_H), 7.46 (d, 1H, Ar-H), 8.10 (d, 1H, Ar-H), 8.50 (d, 1H, pyridine-C_4_H), 9.00 (d, 1H, pyridine-C_2_H) (Figure S6); ^13^C-NMR (DMSO-*d*_*6*_) *δ* ppm: 14.1, 67.0, 89.7, 114.5 (2 C), 122.4, 122.9, 127.7, 127.9, 128.2, 129.1, 137.1, 142.0, 147.0, 151.4, 158.8, 159.9, 176.0 (Figure S7). MS (m/z, %): 390 (M^+^, 59). Anal. Calced for C_18_H_10_N_6_OS_2_ (390.44): C, 55.37; H, 2.58; N, 21.53%. Found: C, 55.29; H, 2.50; N, 21.47%.

### Synthesis of 4,4-dimethyl-2,6-dioxo-1-(thiazolo[5,4-f]quinolin-2-yl)piperidine-3,5-dicarbonitrile (6)

For 6 h, compound **2** (2.60 g, 0.01 mol) and ethyl 3-cyano-2-but-2-enate **(5)** (2.01 g) were refluxed in ethanol (20 mL) and sodium ethoxide (0.68 g, 0.01 mol). After cooling to room temperature, the reaction mixture was filtered, dried, and recrystallized from ethanol to provide the desired product **6**.

Yield, 76%; m.p. 247–249 °C; IR (KBr): *ν*_*max*_, cm^− 1^: 2219 (CN), 1670 (amidic CO), 1622 (C = N); ^1^H-NMR (DMSO-*d*_*6*_) *δ* ppm: 0.91 (s, 6 H, 2CH_3_), 3.09 (s, 2 H, 2CH), 7.40 (m, 1H, pyridine-C_3_H), 7.51 (d, 1H, Ar-H), 8.16 (d, 1H, Ar-H), 8.60 (d, 1H, pyridine-C_4_H), 8.95 (d, 1H, pyridine-C_2_H) (Figure S8); ^13^C-NMR (DMSO-*d*_*6*_) *δ* ppm: 18.2, 21.0 (2 C), 43.6 (2 C), 116.1 (2 C), 120.8, 123.6, 126.6, 127.6, 128.8, 130.9, 137.0, 145.0, 149.5, 160.5, 170.6 (2 C) (Figure S9). MS (m/z, %): 375 (M^+^, 50). Anal. Calced for C_19_H_13_N_5_O_2_S (375.41): C, 60.79; H, 3.49; N, 18.66%. Found: C, 60.71; H, 3.42; N, 18.60%.

### Synthesis of 3-amino-N-(thiazolo[5,4-f]quinolin-2-yl)-3-thioxopropanamide (7)

Hydrogen sulfide (H_2_S) gas was passed through a solution of compound **2** (2.60 g, 0.01 mol) in pyridine (20 ml) and TEA (4 drops) until the increase in the weight of the solution is (2 g). The reaction mixture was refluxed for five hours and then left to cool at room temperature. The desired product **7** was obtained by filtering off the solid, drying it, and then recrystallizing it from ethanol.

Yield, 71%; m.p. 198–200 °C; IR (KBr): *ν*_*max*_, cm^− 1^: 3410 (NH_2_), 3320 (NH), 1682 (CO), 1150 (C = S) (Figure S10); ^1^H-NMR (DMSO-*d*_*6*_) *δ* ppm: 3.73 (s, 2 H, CH_2_), 6.73 (s, 2 H, NH_2_), 7.45 (m, 1H, pyridine-C_3_H), 7.70 (d, 1H, Ar-H), 8.03 (d, 1H, Ar-H), 8.40 (d, 1H, pyridine-C_4_H), 8.89 (d, 1H, pyridine-C_2_H), 12.30 (s, 1H, NH) (Figure S11); ^13^C-NMR (DMSO-*d*_*6*_) *δ* ppm: 52.0, 122.0, 123.6, 126.8, 127.0, 127.6, 129.0, 137.3, 144.1, 150.0, 164.9, 175.0, 184.9 (Figure S12). MS (m/z, %): 302 (M^+^, 66) (Figure S13). Anal. Calced for C_13_H_10_N_4_OS_2_ (302.37): C, 51.64; H, 3.33; N, 18.53%. Found: C, 51.59; H, 3.28; N, 18.45%.

### Synthesis of 4,6-dimethyl-N-(thiazolo[5,4-f]quinolin-2-yl)-2-thioxo-2,3-dihydropyridine-3-carboxamide (8)

A solution of compound **7** (3.00 g, 0.01 mol) and pentan-2,4-dione (1 g, 0.01 mol) in absolute ethanol (25 mL) was refluxed for 4 h in the presence of TEA (4 drops). To obtain the desired product **8**, the reaction mixture was cooled to room temperature and then poured over crushed ice. The resulting solid was then filtered out, dried, and recrystallized from ethanol.

Yield, 74%; m.p. 216–218 °C; IR (KBr): *ν*_*max*_, cm^− 1^: 3230 (NH), 1675 (CO), 1618 (C = N) (Figure S14); ^1^H-NMR (DMSO-*d*_*6*_) *δ* ppm: 1.82 (s, 3 H, C_4_-CH_3_), 2.33 (s, 3 H, C_6_-CH_3_), 3.12 (s, 1H, pyridine-C_3_H), 5.95 (s, 1H, pyridine-C_5_H), 7.60 (m, 1H, quinoline-C_3_H), 7.69 (d, 1H, Ar-H), 8.09 (d, 1H, Ar-H), 8.55 (d, 1H, quinoline-C_4_H), 8.89 (d, 1H, quinoline-C_2_H), 12.66 (s, 1H, NH); ^13^C-NMR (DMSO-*d*_*6*_) *δ* ppm: 17.8, 20.0, 66.9, 109.5, 122.0, 123.5, 126.0, 126.9, 129.5, 130.0, 137.1 (2 C), 143.2, 149.7, 161.9, 174.7 (2 C), 197.8 (Figure S15). MS (m/z, %): 366 (M^+^, 61) (Figure S16). Anal. Calced for C_18_H_14_N_4_OS_2_ (366.46): C, 59.00; H, 3.85; N, 15.29%. Found: C, 58.92; H, 3.79; N, 15.22%.

### Synthesis of 2-(4,6-diamino-3,5-dicyanopyridin-2-yl)-N-(thiazolo[5,4-f]quinolin-2-yl)acetamide (10)

Compound **2** (2.60 g, 0.01 mol) and 2-aminoprop-1-ene-1,1,3-tricarbonitrile **(9)** (1.32 g, 0.01 mol) underwent a reaction in a pressure tube in an silicon oil bath at 150 °C. After cooling, the reaction mixture was triturated with ethanol. To obtain the required product **10**, the solid material was filtered, dried, and recrystallized from ethanol-DMF (1:1, 20 mL).

Yield, 79%; m.p. 244–246 °C; IR (KBr): *ν*_*max*_, cm^− 1^: 3450 (NH_2_), 3235 (NH), 2219 (CN), 1680 (CO), 1618 (C = N) (Figure S17); ^1^H-NMR (DMSO-*d*_*6*_) *δ* ppm: 4.22 (s, 2 H, CH_2_), 6.44 (s, 2 H, NH_2_), 6.94 (s, 2 H, NH_2_), 7.76 (m, 1H, quinoline-C_3_H), 7.88 (d, 1H, Ar-H), 8.03 (d, 1H, Ar-H), 8.43 (d, 1H, quinoline-C_4_H), 9.05 (d, 1H, quinoline-C_2_H), 12.66 (s, 1H, NH); ^13^C-NMR (DMSO-*d*_*6*_) *δ* ppm: 37.5, 76.0, 85.8, 115.0 116.0, 120.1, 124.0, 126.7, 127.8, 128.0, 128.9, 136.7, 144.5, 149.8, 166.0, 166.5, 166.8, 169.5, 174.0 (Figure S18). MS (m/z, %): 400 (M^+^, 64) (Figure S19). Anal. Calced for C_19_H_12_N_8_OS (400.42): C, 56.99; H, 3.02; N, 27.98%. Found: C, 56.93; H, 2.91; N, 27.90%.

### Synthesis of 2-(6-cyano-4-oxo-5,7-diphenyl-3,5-dihydro-4 H-pyrano[2,3-d]pyrimidin-2-yl)-N-(thiazolo[5,4-f]quinolin-2-yl)acetamide (12)

In the presence of 15 mL of pyridine, compound **2** (2.60 g, 0.01 mol) and 2-amino-3-carboxy-5-cyano-4,6-diphenyl-4 H-pyran **(11)** (3.46 g, 0.01 mol) were refluxed for 6 h. After pouring the reaction mixture onto crushed ice, it was left to cool to room temperature. To obtain the dihydropyrano[2,3-d] derivative of pyrimidinone **12**, the solid was separated from the ethanol by filtering, drying, and recrystallizing.

Yield, 71%; m.p. 263–265 °C; IR (KBr): *ν*_*max*_, cm^− 1^: 3240 (br. NH), 2220 (CN), 1681 (CO), 1618 (C = N); ^1^H-NMR (DMSO-*d*_*6*_) *δ* ppm: 3.22 (s, 2 H, CH_2_), 4.35 (s, 1H, pyrane-CH), 7.19–7.88 (m, 12 H, Ar-H + pyridine-C_3_H), 8.11 (d, 1H, Ar-H), 8.55 (d, 1H, pyridine-C_4_H), 8.91 (d, 1H, pyridine-C_2_H), 12.33 (s, 1H, pyrimidine-NH), 12.55 (s, 1H, NH); ^13^C-NMR (DMSO-*d*_*6*_) *δ* ppm: 37.1, 38.0, 92.8, 100.5, 117.3, 121.5, 122.8, 122.9, 124.0, 126.8, 127.8 (5 C), 130.0 (5 C), 130.9 (2 C), 137.6, 142.0, 142.7, 150.0, 158.5, 159.4, 161.7, 162.1, 166.0, 175.0 (Figure S20). MS (m/z, %): 568 (M^+^, 57). Anal. Calced for C_32_H_20_N_6_O_3_S (568.61): C, 67.59; H, 3.55; N, 14.78%. Found: C, 67.50; H, 3.49; N, 14.71%.

### Synthesis of 2-cyano-3-(dimethylamino)-N-(thiazolo[5,4-f]quinolin-2-yl)acrylamide (13)

Dimethylformamide/dimethylacetal (1.19 g, 0.01 mol) and compound **2** (2.60 g, 0.01 mol) were refluxed for 7 h in dry xylene (20 mL). The dihydropyrano[2,3 d]pyrimidinone derivative **13** was obtained by drying, recrystallizing, and separating the resultant solid from the ethanol.

Yield, 72%; m.p. 211–213 °C; IR (KBr): *ν*_*max*_, cm^− 1^: 3230 (NH), 2219 (CN), 1676 (CO), 1635 (C = N) (Figure S21); ^1^H-NMR (DMSO-*d*_*6*_) *δ* ppm: 3.09 (s, 6 H, 2CH_3_), 7.50 (s, 1H, olefinic-CH), 7.60 (m, 1H, pyridine-C_3_H), 7.69 (d, 1H, Ar-H), 8.30 (d, 1H, Ar-H), 8.67 (d, 1H, pyridine-C_4_H), 9.17 (d, 1H, pyridine-C_2_H), 12.76 (s, 1H, NH) (Figure S22); ^13^C-NMR (DMSO-*d*_*6*_) *δ* ppm: 44.1 (2 C), 99.6, 115.2, 121.5, 123.9, 124.9, 127.2, 128.0, 130.4, 136.7, 145.1, 149.6, 157.3, 163.7, 174.4 (Figure S23). MS (m/z, %): 323 (M^+^, 60) (Figure S24). Anal. Calced for C_16_H_13_N_5_OS (323.37): C, 59.43; H, 4.05; N, 21.66%. Found: C, 59.37; H, 3.98; N, 21.59%.

### Synthesis of 5-amino-2-phenyl-N-(thiazolo[5,4-f]quinolin-2-yl)pyrazolo[1,5-a]pyrimidine-6-carboxamide (14)

Compound **13** (4.08 g, 0.01 mol) and 3-phenyl-1 H-pyrazol-5-amine was heated for 5 h in the presence of DMF (25 mL) and a few drops of piperidine (1.59 gm, 0.01 mol). The solid was filtered out, dried, and recrystallized from ethanol to provide the dihydropyrano[2,3-d]pyrimidinone derivative **14**.

Yield, 74%; m.p. 251–253 °C; IR (KBr): *ν*_*max*_, cm^− 1^: 3420 (NH_2_), 3320 (NH), 1710 (CO) (Figure S25); ^1^H-NMR (DMSO-*d*_*6*_) *δ* ppm: 6.57 (s, 1H, pyrazole-CH), 6.80 (s, 2 H, NH_2_), 7.58–7.90 (m, 8 H, Ar-H + pyridine-C_3_H), 8.60 (d, 1H, pyridine-C_4_H), 8.90 (s, 1H, pyrimidine-CH), 9.24 (d, 1H, pyridine-C_2_H), 12.70 (s, 1H, NH) (Figure S26); ^13^C-NMR (DMSO-*d*_*6*_) *δ* ppm: 92.6, 113.0, 121.7, 124.8, 125.1, 127.8 (3 C), 128.6 (2 C), 128.8 (3 C), 134.2, 138.8, 143.0 (2 C), 150.5 (2 C), 154.5, 154.9, 164.0, 174.0 (Figure S27). MS (m/z, %): 437 (M^+^, 65). Anal. Calced for C_23_H_15_N_7_OS (437.48): C, 63.15; H, 3.46; N, 22.41%. Found: C, 63.09; H, 3.37; N, 22.35%.

### Synthesis of compound 15

A stirred suspension of potassium hydroxide (0.23 g) in DMF (20 mL) was supplemented with compound **2** (2.60 g, 0.01 mol). After adding phenyl isothiocyanate (1.35 g, 0.01 mol) to the resulting solution, the reaction mixture was stirred overnight at room temperature.

### Synthesis of acyclic intermediates 16, 18, 20, 22, and 24

#### General procedure

For 5 h at room temperature, ethanol and α-chloroacetamide, naphthoyl acetyl bromide, chloroacetone, chloroacetonitrile, and/or ethyl bromoacetate were combined with equimolecular quantities of **15** (0.01 mol). After that, they were left to stand overnight at that temperature. Compounds **16**, **18**, **20**, **22**, and **24** were obtained by filtering out, drying, rinsing with water, and crystallizing the reaction mixture from ethanol.

### 2-((1-Cyano-3-oxo-2-(phenylamino)-3-(thiazolo[5,4-f]quinolin-2-yl)prop-1-en-1-yl)thio)acetamide (16)

Yield, 71%; m.p. 205–207 °C; IR (KBr): *ν*_*max*_, cm^− 1^: 3340 (NH_2_), 3150 (NH), 2220 (CN), 1700 (CO), 1680 (CO); ^1^H-NMR (DMSO-*d*_*6*_) *δ* ppm: 3.97 (s, 2 H, CH_2_), 6.96–7.35 (m, 9 H, Ar-H + NH_2_ + pyridine-C_3_H), 8.00 (d, 1H, Ar-H), 8.41 (d, 1H, pyridine-C_4_H), 8.80 (d, 1H, pyridine-C_2_H), 10.80 (s, 1H, NH) (Figure S28); ^13^C-NMR (DMSO-*d*_*6*_) *δ* ppm: 35.4, 101.4, 115.9, 120.5 (2 C), 121.8, 125.4 (2 C), 128.0, 128.9 (2 C), 129.5 (2 C), 130.0, 137.8, 142.4, 143.1, 147.4, 151.5, 161.0, 170.6, 188.0 (Figure S29). MS (m/z, %): 445 (M^+^, 55) (Figure S30). Anal. Calced for C_22_H_15_N_5_O_2_S_2_ (445.52): C, 59.31; H, 3.39; N, 15.72%. Found: C, 59.25; H, 3.30; N, 15.66%.

### 3-((2-(Naphthalen-2-yl)-2-oxoethyl)thio)-3-(phenylamino)-2-(thiazolo[5,4-f]quinoline-2-carbonyl)acrylonitrile (18)

Yield, 78%; m.p. 261–263 °C; IR (KBr): *ν*_*max*_, cm^− 1^: 3235 (NH), 2217 (CN), 1732 (CO), 1700 (CO), 1649 (C = N) (Figure S33); ^1^H-NMR (DMSO-*d*_*6*_) *δ* ppm: 4.70 (s, 2 H, CH_2_), 6.91–7.50 (m, 13H, Ar-H + pyridine-C_3_H), 8.10 (d, 1H, Ar-H), 8.50 (d, 1H, pyridine-C_4_H), 8.89 (s, 1H, Ar-H), 9.27 (d, 1H, pyridine-C_2_H), 10.79 (s, 1H, NH) (Figure S34); ^13^C-NMR (DMSO-*d*_*6*_) *δ* ppm: 37.0, 69.0, 117.4, 120.3, 122.0, 122.9, 123.5, 123.7 (2 C), 124.2 (2 C), 125.1 (2 C), 126.7 (3 C), 129.4 (4 C), 131.7, 136.0 (2 C), 136.5, 138.0 (2 C), 141.9, 148.9, 161.8, 180.7, 187.8, 194.0 (Figure S35). MS (m/z, %): 556 (M^+^, 58). Anal. Calced for C_32_H_20_N_4_O_2_S_2_ (556.66): C, 69.05; H, 3.62; N, 10.07%. Found: C, 68.99; H, 3.59; N, 10.00%.

### 3-((2-Oxopropyl)thio)-3-(phenylamino)-2-(thiazolo[5,4-f]quinoline-2-carbonyl)acrylonitrile (20)

Yield, 71%; m.p. 244–246 °C; IR (KBr): *ν*_*max*_, cm^− 1^: 3250 (NH), 2218 (CN), 1725 (CO), 1720 (CO), 1622 (C = N); ^1^H-NMR (DMSO-*d*_*6*_) *δ* ppm: 2.34 (s, 3 H, CH_3_), 4.09 (s, 2 H, CH_2_), 6.95–7.77 (m, 7 H, Ar-H + pyridine-C_3_H), 8.13 (d, 1H, Ar-H), 8.49 (d, 1H, pyridine-C_4_H), 8.91 (d, 1H, pyridine-C_2_H), 10.81 (s, 1H, NH); ^13^C-NMR (DMSO-*d*_*6*_) *δ* ppm: 16.7, 42.0, 69.5, 117.1, 121.6 (2 C), 123.1, 123.9, 124.0 (2 C), 127.0 (2 C), 129.0 (2 C), 131.1, 136.1 (2 C), 143.5, 148.0, 159.1, 178.8, 187.5, 200.9 (Figure S39). MS (m/z, %): 444 (M^+^, 63) (Figure S40). Anal. Calced for C_23_H_16_N_4_O_2_S_2_ (444.53): C, 62.15; H, 3.63; N, 12.60%. Found: C, 62.09; H, 3.58; N, 12.54%.

### 3-((Cyanomethyl)thio)-3-(phenylamino)-2-(thiazolo[5,4-f]quinoline-2-carbonyl)acrylonitrile (22)

Yield, 70%; m.p. 187–189 °C; IR (KBr): *ν*_*max*_, cm^− 1^: 3210 (NH), 2220 (CN), 2200 (CN), 1710 (CO), 1639 (C = N) (Figure S43); ^1^H-NMR (DMSO-*d*_*6*_) *δ* ppm: 4.29 (s, 2 H, CH_2_), 7.20–7.58 (m, 7 H, Ar-H + pyridine-C_3_H), 8.10 (d, 1H, Ar-H), 8.50 (d, 1H, pyridine-C_4_H), 8.90 (d, 1H, pyridine-C_2_H), 10.70 (s, 1H, NH) (Figure S44); ^13^C-NMR (DMSO-*d*_*6*_) *δ* ppm: 16.9, 69.8, 113.1, 117.3, 121.9 (2 C), 122.0, 123.4, 123.8, 124.0, 125.5 (2 C), 128.0, 129.4 (2 C), 130.6, 135.0 (2 C), 143.0, 147.6, 161.0, 178.7, 189.8 (Figure S45). MS (m/z, %): 427 (M^+^, 65). Anal. Calced for C_22_H_13_N_5_OS_2_ (427.50): C, 61.81; H, 3.07; N, 16.38%. Found: C, 61.75; H, 3.00; N, 16.31%.

### Ethyl 2-((2-cyano-3-oxo-1-(phenylamino)-3-(thiazolo[5,4-f]quinolin-2-yl)prop-1-en-1-yl)thio)acetate (24)

Yield, 71%; m.p. 216–218 °C; IR (KBr): *ν*_*max*_, cm^− 1^: 3230 (NH), 2219 (CN), 1725 (CO), 1620 (C = N); ^1^H-NMR (DMSO-*d*_*6*_) *δ* ppm: 1.27 (t, 3 H, CH_3_), 3.99 (s, 2 H, CH_2_), 4.22 (q, 2 H, CH_2_), 6.99–7.50 (m, 7 H, Ar-H + pyridine-C_3_H), 8.05 (d, 1H, Ar-H), 8.45 (d, 1H, pyridine-C_4_H), 8.88 (d, 1H, pyridine-C_2_H), 10.66 (s, 1H, NH); ^13^C-NMR (DMSO-*d*_*6*_) *δ* ppm: 14.9, 33.9, 61.0, 68.9, 114.2, 120.5, 121.9, 122.0, 122.6, 123.6 (2 C), 124.2 (2 C), 127.1 (2 C), 130.8, 135.5 (2 C), 144.4, 148.8, 160.7, 170.2, 180.1, 188.0 (Figure S49). MS (m/z, %): 474 (M^+^, 56) (Figure S50). Anal. Calced for C_24_H_18_N_4_O_3_S_2_ (474.55): C, 60.74; H, 3.82; N, 11.81%. Found: C, 60.69; H, 3.75; N, 11.75%.

### Synthesis of thiophene derivatives (17, 19, 21, 23 and 25)

**Method A:** In 20 mL of DMF in the presence of TEA, an equimolecular amounts of **15** (0.01 mol) and α-halo compounds (0.01 mol) were mixed and refluxed for six hours. The reaction mixture produced the corresponding thiophene derivatives **17**, **19**, **21**, **23**, and **25**, respectively, following cooling, filtering, and recrystallization from ethanol.

**Method B:** The corresponding substituted thiophene derivatives, **17**, **19**, **21**, **23**, and **25**, were obtained by refluxing the acyclic intermediates **16**, **18**, **20**, **22**, and/or **24** for three hours in ethanol (20 mL) containing a catalytic quantity of TEA (4 drops).

### 5-Amino-3-(phenylamino)-4-(thiazolo[5,4-f]quinoline-2-carbonyl)thiophene-2-carboxamide (17)

By using diluted HCl (5 mL) to acidify intermediate **15** (0.01 mol) until the medium become acidic, compound **17** was created. After that, solid product **17** was separated from the aqueous ethanol by filtering, washing with water, drying, and recrystallizing.

Yield, 76%; m.p. 227–229 °C; IR (KBr): *ν*_*max*_, cm^− 1^: 3450 (NH_2_), 3155 (NH), 1705 (CO), 1661 (amidic CO); ^1^H-NMR (DMSO-*d*_*6*_) *δ* ppm: 6.68 (s, 2 H, CO-NH_2_), 7.19–7.58 (m, 9 H, Ar-H + NH_2_ + pyridine-C_3_H), 7.97 (d, 1H, Ar-H), 8.34 (d, 1H, pyridine-C_4_H), 8.70 (d, 1H, pyridine-C_2_H), 10.49 (s, 1H, NH) (Figure S31); ^13^C-NMR (DMSO-*d*_*6*_) *δ* ppm: 115.8 (2 C), 121.5, 122.0, 123.1, 124.0, 125.7, 126.7, 127.6 (3 C), 132.9 (2 C), 137.7 (2 C), 142.1, 146.9, 151.0, 161.5, 162.1, 175.6, 182.4 (Figure S32). MS (m/z, %): 445 (M^+^, 51). Anal. Calced for C_22_H_15_N_5_O_2_S_2_ (445.52): C, 59.31; H, 3.39; N, 15.72%. Found: C, 59.22; H, 3.32; N, 15.64%.

### (5-(2-Naphthoyl)-4-amino-2-(phenylamino)thiophen-3-yl)(thiazolo[5,4-f]quinolin-2-yl)methanone (19)

Yield, 74%; m.p. 280–282 °C; IR (KBr): *ν*_*max*_, cm^− 1^: 3450 (NH_2_), 3230 (NH), 1731 (CO), 1700 (CO), 1625 (C = N) (Figure S36); ^1^H-NMR (DMSO-*d*_*6*_) *δ* ppm: 5.96 (s, 2 H, NH_2_), 7.50–8.22 (m, 14 H, Ar-H + pyridine-C_3_H), 8.35 (s, 1H, Ar-H), 8.59 (d, 1H, pyridine-C_4_H), 8.99 (d, 1H, pyridine-C_2_H), 10.33 (s, 1H, NH); ^13^C-NMR (DMSO-*d*_*6*_) *δ* ppm: 95.7, 119.4 (2 C), 120.5, 120.9, 122.0, 124.8, 125.1 (2 C), 126.3 (2 C), 129.5 (3 C), 130.7 (3 C), 130.8 (3 C), 132.1 (2 C), 136.0 (2 C), 137.9 (2 C), 142.0, 149.9, 161.9, 170.7, 182.6, 187.5 (Figure S37). MS (m/z, %): 556 (M^+^, 51) (Figure S38). Anal. Calced for C_32_H_20_N_4_O_2_S_2_ (556.66): C, 69.05; H, 3.62; N, 10.07%. Found: C, 68.97; H, 3.55; N, 9.98%.

### 1-(3-Amino-5-(phenylamino)-4-(thiazolo[5,4-f]quinoline-2-carbonyl)thiophen-2-yl)ethan-1-one (21)

Yield, 76%; m.p. 257–259 °C; IR (KBr): *ν*_*max*_, cm^− 1^: 3425 (NH_2_), 3230 (NH), 1730 (CO), 1725 (CO), 1616 (C = N); ^1^H-NMR (DMSO-*d*_*6*_) *δ* ppm: 2.18 (s, 3 H, CH_3_), 5.85 (s, 2 H, NH_2_), 7.05 (m, 1H, Ar-H), 7.36 (d, 4 H, Ar-H), 7.63 (m, 1H, pyridine-C_3_H), 7.90 (d, 1H, Ar-H), 8.16 (d, 1H, Ar-H), 8.48 (d, 1H, pyridine-C_4_H), 8.89 (d, 1H, pyridine-C_2_H), 10.27 (s, 1H, NH) (Figure S41); ^13^C-NMR (DMSO-*d*_*6*_) *δ* ppm: 26.4, 117.8 (2 C), 121.9, 123.1, 124.0, 124.9, 126.0 (2 C), 129.0 (2 C), 130.0, 133.9, 136.0 (2 C), 142.5, 144.9, 148.7, 152.8, 159.9, 169.4, 183.8, 191.0 (Figure S42). MS (m/z, %): 444 (M^+^, 68). Anal. Calced for C_23_H_16_N_4_O_2_S_2_ (444.53): C, 62.15; H, 3.63; N, 12.60%. Found: C, 62.10; H, 3.55; N, 12.52%.

### 3-Amino-5-(phenylamino)-4-(thiazolo[5,4-f]quinoline-2-carbonyl)thiophene-2-carbonitrile (23)

Yield, 74%; m.p. 224–226 °C; IR (KBr): *ν*_*max*_, cm^− 1^: 3420 (NH_2_), 3230 (NH), 2219 (CN), 1735 (CO) (Figure S46); ^1^H-NMR (DMSO-*d*_*6*_) *δ* ppm: 5.73 (s, 2 H, NH_2_), 7.11 (m, 1H, Ar-H), 7.44 (d, 4 H, Ar-H), 7.66 (m, 1H, pyridine-C_3_H), 7.75 (d, 1H, Ar-H), 8.17 (d, 1H, Ar-H), 8.43 (d, 1H, pyridine-C_4_H), 8.96 (d, 1H, pyridine-C_2_H), 10.22 (s, 1H, NH); ^13^C-NMR (DMSO-*d*_*6*_) *δ* ppm: 85.8, 113.1, 117.6 (2 C), 121.1, 122.3, 123.9, 126.7, 127.7, 128.9 (4 C), 133.5, 136.0 (2 C), 140.0, 142.1, 148.6, 158.4, 167.9, 182.7 (Figure S47). MS (m/z, %): 427 (M^+^, 50) (Figure S48). Anal. Calced for C_22_H_13_N_5_OS_2_ (427.50): C, 61.81; H, 3.07; N, 16.38%. Found: C, 61.72; H, 2.97; N, 16.29%.

### Ethyl 3-amino-5-(phenylamino)-4-(thiazolo[5,4-f]quinoline-2-carbonyl)thiophene-2-carboxylate (25)

Yield, 73%; m.p. 233–235 °C; IR (KBr): *ν*_*max*_, cm^− 1^: 3420 (NH_2_), 3230 (NH), 1725 (CO); ^1^H-NMR (DMSO-*d*_*6*_) *δ* ppm: 1.29 (t, 3 H, CH_3_), 4.36 (q, 2 H, CH_2_), 5.40 (s, 2 H, NH_2_), 7.22–7.50 (m, 7 H, Ar-H + pyridine-C_3_H), 8.19 (d, 1H, Ar-H), 8.54 (d, 1H, pyridine-C_4_H), 8.89 (d, 1H, pyridine-C_2_H), 10.30 (s, 1H, NH) (Figure S51); ^13^C-NMR (DMSO-*d*_*6*_) *δ* ppm: 14.5, 60.8, 118.0 (2 C), 120.5, 121.8, 122.1, 123.0, 125.5, 128.1 (2 C), 129.4 (4 C), 133.1, 135.5, 141.9, 144.0, 148.0, 160.5 (2 C), 169.6, 182.9 (Figure S52). MS (m/z, %): 474 (M^+^, 60). Anal. Calced for C_24_H_18_N_4_O_3_S_2_ (474.55): C, 60.74; H, 3.82; N, 11.81%. Found: C, 60.67; H, 3.73; N, 11.75%.

### Antimicrobial activity

It was occurring according to previously reported work^42^.

### Molecular docking

Protein Data Bank (PDB) (https://www.rcsb.org) was utilized to download the crystal structures of receptor molecule *E. coli gyrase B* protein receptor (ID Code: 6F86;)^43^. The chemical structures of compounds 18, 19, and 23 were drawn using (ChemDraw 16.0 professional) tool assigned with proper 2D, 3D, surface Map orientation, and energy of separate molecule was minimized by ChemBio3D Ultra 16.0, which is part of the ChemOffice Professional Collection by PerkinElmer (https://www.perkinelmer.com/product/chembiooffice-ultra-16-0-download-version-cos1600dl). Meanwhile, the target protein was kept in PDB format, which was then exported into M.O.E. program to formulate the ligands and keep them in PDB format. The docked results were visualized by using the M.O.E. with standard protocol to carry out docking of ligand-receptor interactions.

## Conclusion

In this study, a novel series of **thiazolo[5**,**4-f]quinoline-based compounds** incorporating pyridine and thiophene moieties were successfully synthesized and evaluated for antimicrobial activity. Among the tested compounds, **18**,** 19**,** and 23** exhibited **potent broad-spectrum activity**, with **MIC values as low as 3.125 µg/mL** against *Staphylococcus aureus* and *Escherichia coli*, and **6.25 µg/mL** against *Candida albicans*. These values are **comparable to or better than standard drugs** such as Chloramphenicol (MIC = 3.125–6.25 µg/mL). Structure–activity relationship (SAR) analysis indicated that **bulky lipophilic groups** such as naphthoyl and thiophene significantly enhanced activity. Docking studies further validated these findings: **compound 19** showed the **strongest binding affinity** to bacterial DNA gyrase (PDB: 6F86) with a **binding energy of -6.6951 kcal/mol**, forming **multiple hydrogen bonds and π-interactions**. Statistical analysis confirmed the significance of microbial response variation (ANOVA *p* = 0.0122), with *S. aureus* being the most susceptible (mean MIC = 25.82 µg/mL) and *C. albicans* the most resistant (mean MIC = 56.25 µg/mL). These findings underscore the **potential of thiazoloquinoline derivatives**, especially those bearing thiophene and naphthoyl functionalities, as **promising antimicrobial candidates** worthy of further preclinical development.

## Supplementary Information

Below is the link to the electronic supplementary material.

Supplementary Material 1

## Data Availability

No datasets were generated or analysed during the current study.
