# Supplementary material for: Exploring novel thiazolo[5,4-f]quinoline-based scaffolds as promising antimicrobial agents through synthesis and molecular insights
Source: Sci Rep. 2025 Aug 26;15:31486. doi: 10.1038/s41598-025-16561-w (PMC12381138; doi:10.1038/s41598-025-16561-w)
Supplement: Supplementary file 1 — Supplementary Material 1 [file 41598_2025_16561_MOESM1_ESM.docx]

**Index**

| **Contents** | **Page** |
| --- | --- |
| **Figure S1.** IR spectrum of compound **2** | **3** |
| **Figure S2.** ^1^H-NMR of compound **2** | **4** |
| **Figure S3.** ^13^C-NMR of Compound **2** | **5** |
| **Figure S4.** Mass spectrum of compound **2** | **6** |
| **Figure S5.** IR spectrum of compound **4** | **7** |
| **Figure S6.** ^1^H-NMR of compound **4** | **8** |
| **Figure S7.** ^13^C-NMR of Compound **4** | **9** |
| **Figure S8.** ^1^H-NMR of compound **6** | **10** |
| **Figure S9.** ^13^C-NMR of Compound **6** | **11** |
| **Figure S10.** IR spectrum of compound **7** | **12** |
| **Figure S11.** ^1^H-NMR of compound **7** | **13** |
| **Figure S12.** ^13^C-NMR of Compound **7** | **14** |
| **Figure S13.** Mass spectrum of compound **7** | **15** |
| **Figure S14.** IR spectrum of compound **8** | **16** |
| **Figure S15.** ^13^C-NMR of Compound **8** | **17** |
| **Figure S16.** Mass spectrum of compound **8** | **18** |
| **Figure S17.** IR spectrum of compound **10** | **19** |
| **Figure S18.** ^13^C-NMR of Compound **10** | **20** |
| **Figure S19.** Mass spectrum of compound **10** | **21** |
| **Figure S20.** ^13^C-NMR of Compound **12** | **22** |
| **Figure S21.** IR spectrum of compound **13** | **23** |
| **Figure S22.** ^1^H-NMR of compound **13** | **24** |
| **Figure S23.** ^13^C-NMR of Compound **13** | **25** |
| **Figure S24.** Mass spectrum of compound **13** | **26** |
| **Figure S25.** IR spectrum of compound **14** | **27** |
| **Figure S26.** ^1^H-NMR of compound **14** | **28** |
| **Figure S27.** ^13^C-NMR of Compound **14** | **29** |
| **Figure S28.** ^1^H-NMR of compound **16** | **30** |
| **Figure S29.** ^13^C-NMR of Compound **16** | **31** |
| **Figure S30.** Mass spectrum of compound **16** | **32** |
| **Figure S31.** ^1^H-NMR of compound **17** | **33** |
| **Figure S32.** ^13^C-NMR of Compound **17** | **34** |
| **Figure S33.** IR spectrum of compound **18** | **35** |
| **Figure S34.** ^1^H-NMR of compound **18** | **36** |
| **Figure S35.** ^13^C-NMR of Compound **18** | **37** |
| **Figure S36.** IR spectrum of compound **19** | **38** |
| **Figure S37.** ^13^C-NMR of Compound **19** | **39** |
| **Figure S38.** Mass spectrum of compound **19** | **40** |
| **Figure S39.** ^13^C-NMR of Compound **20** | **41** |
| **Figure S40.** Mass spectrum of compound **20** | **42** |
| **Figure S41.** ^1^H-NMR of compound **21** | **43** |
| **Figure S42.** ^13^C-NMR of Compound **21** | **44** |
| **Figure S43.** IR spectrum of compound **22** | **45** |
| **Figure S44.** ^1^H-NMR of compound **22** | **46** |
| **Figure S45.** ^13^C-NMR of Compound **22** | **47** |
| **Figure S46.** IR spectrum of compound **23** | **48** |
| **Figure S47.** ^13^C-NMR of Compound **23** | **49** |
| **Figure S48.** Mass spectrum of compound **23** | **50** |
| **Figure S49.** ^13^C-NMR of Compound **24** | **51** |
| **Figure S50.** Mass spectrum of compound **24** | **52** |
| **Figure S51.** ^1^H-NMR of compound **25** | **53** |
| **Figure S52.** ^13^C-NMR of Compound **25** | **54** |

**
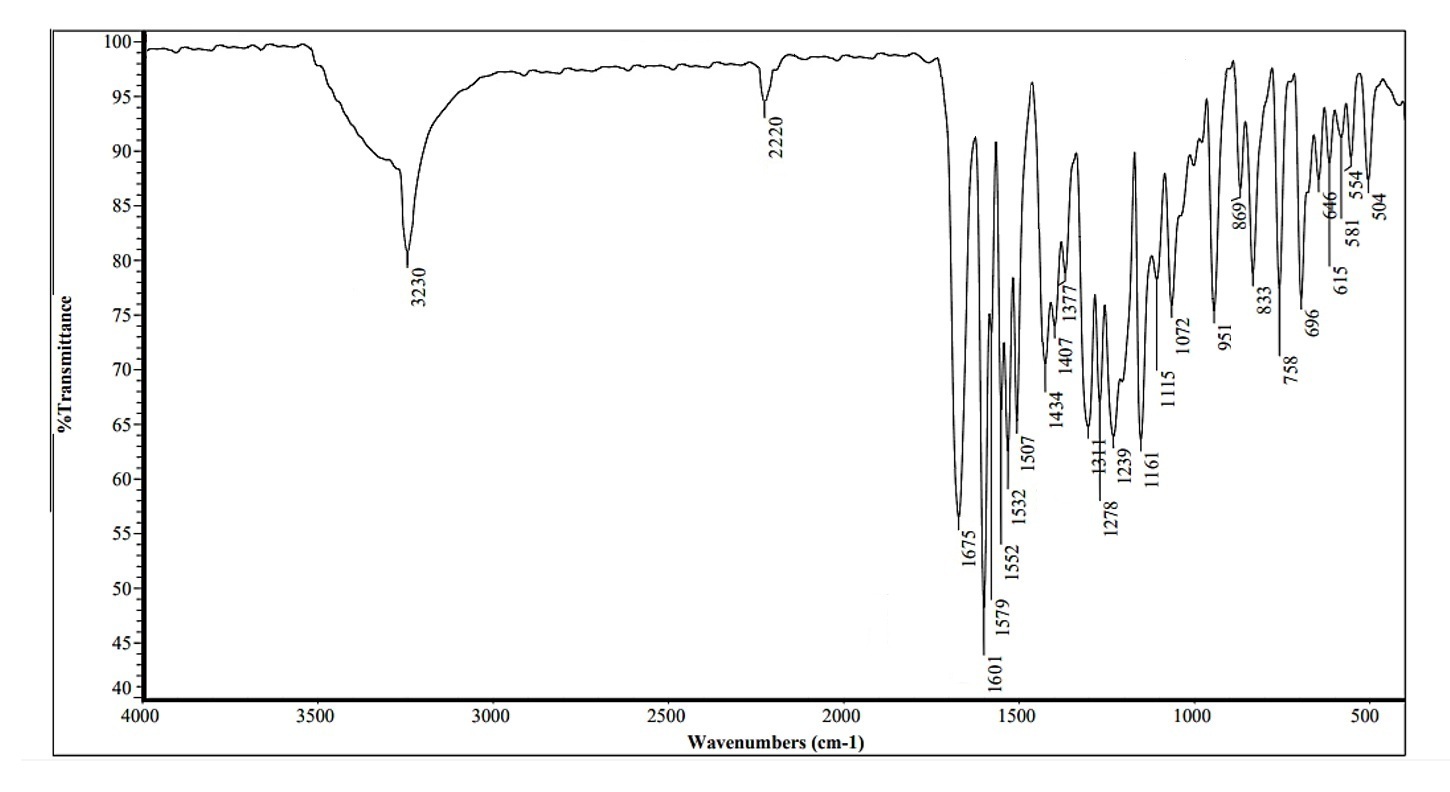
**

**Figure S1.** IR spectrum of compound **2**

IR (KBr): *ν_max_*, cm^-1^: 3230 (NH), 2220 (CN), 1675 (CO), 1601 (C=N) functional groups


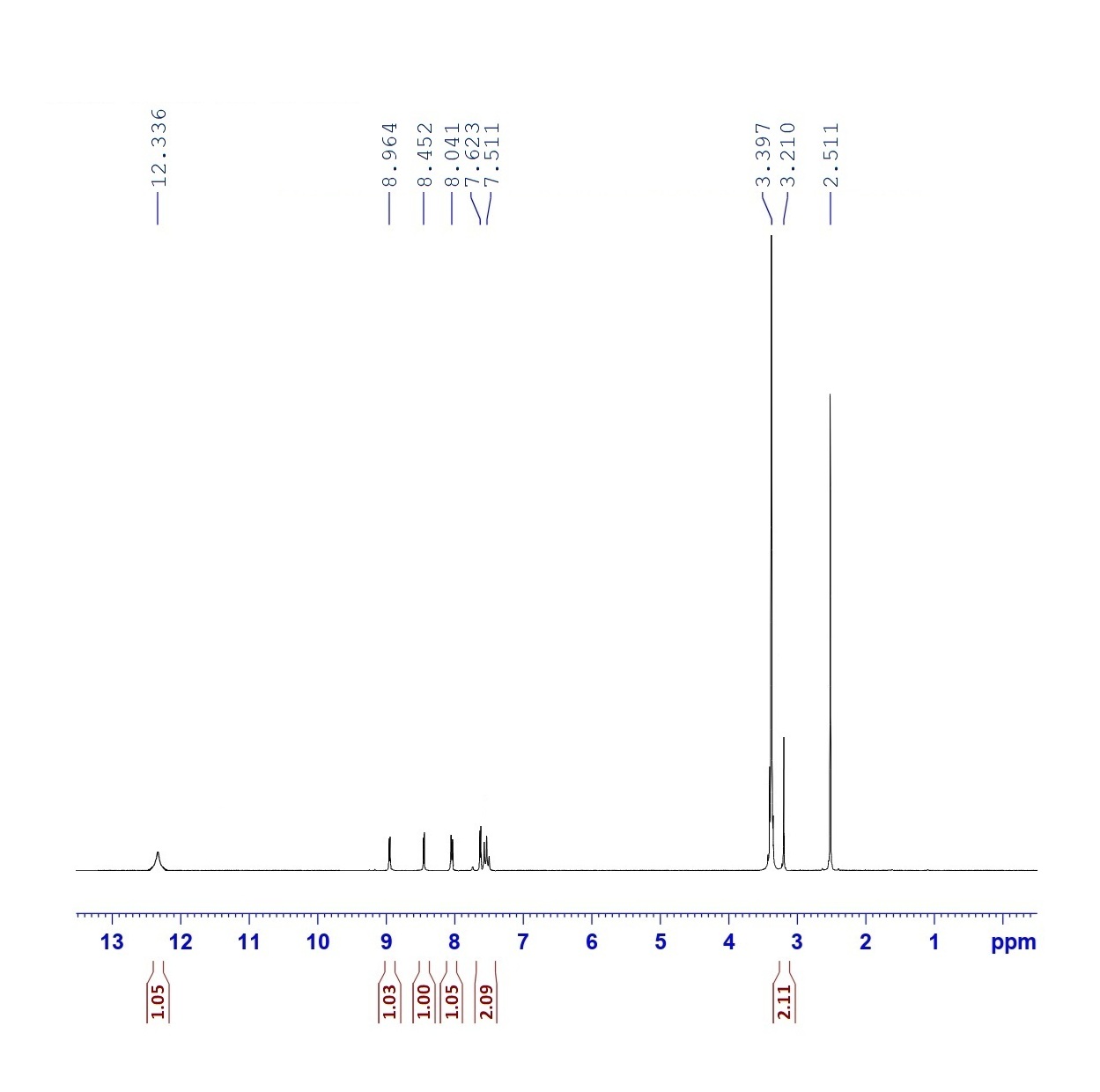


**Figure S2.** ^1^H-NMR of compound **2**

^1^H-NMR (DMSO-*d_6_*) *δ* ppm: 3.21 (s, 2H, CH_2_), 7.51 (m, 1H, pyridine-C_3_H), 7.62 (d, 1H, Ar-H), 8.04 (d, 1H, Ar-H), 8.45 (d, 1H, pyridine-C_4_H), 8.96 (d, 1H, pyridine-C_2_H), 12.33 (s, 1H, NH)

**
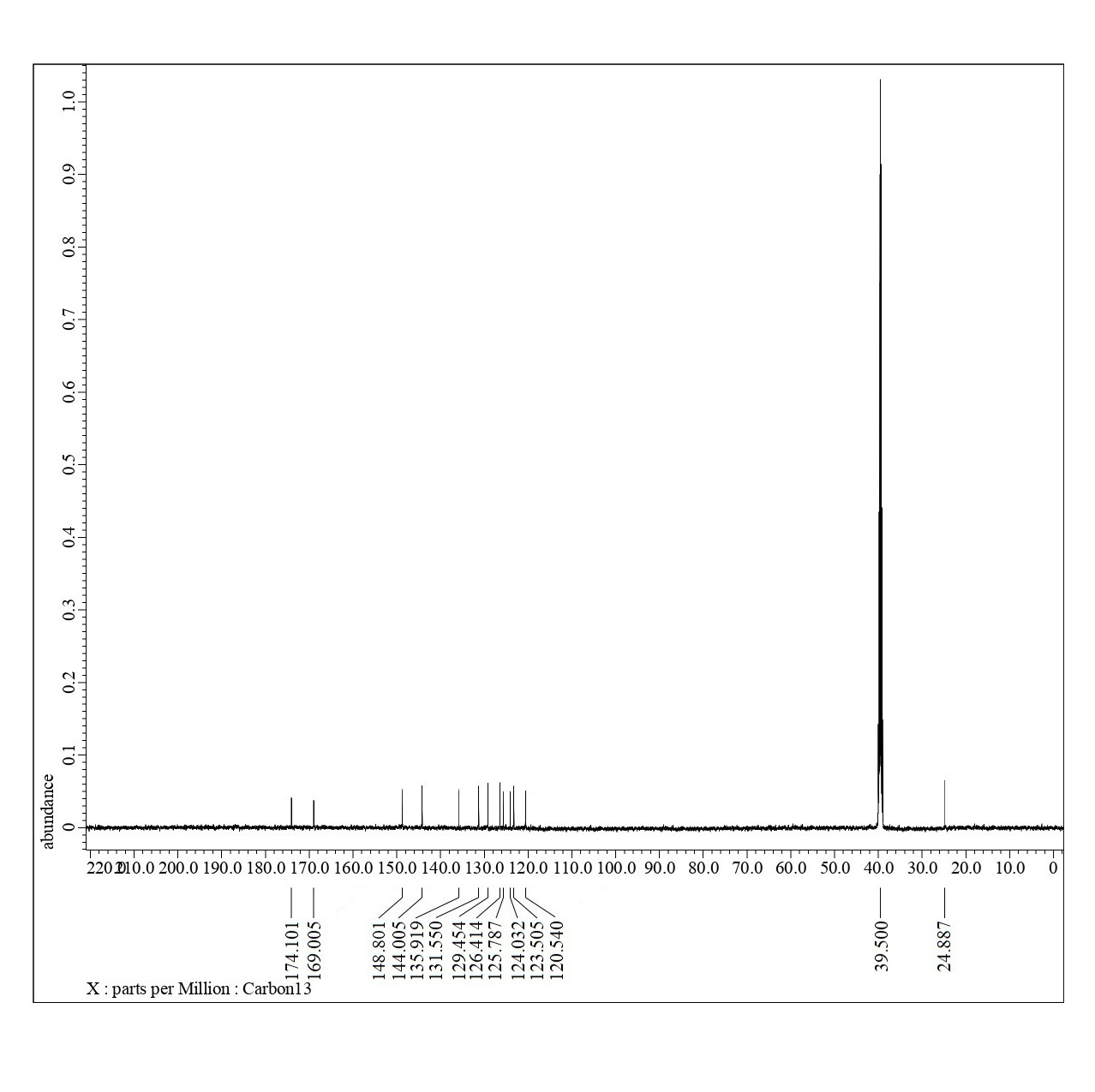
**

**Figure S3.** ^13^C-NMR of Compound **2**

The ^13^C-NMR spectrum reveals the expected chemical shifts for the carbon atoms in the thiazole and quinoline rings, as well as the substituent groups.

**
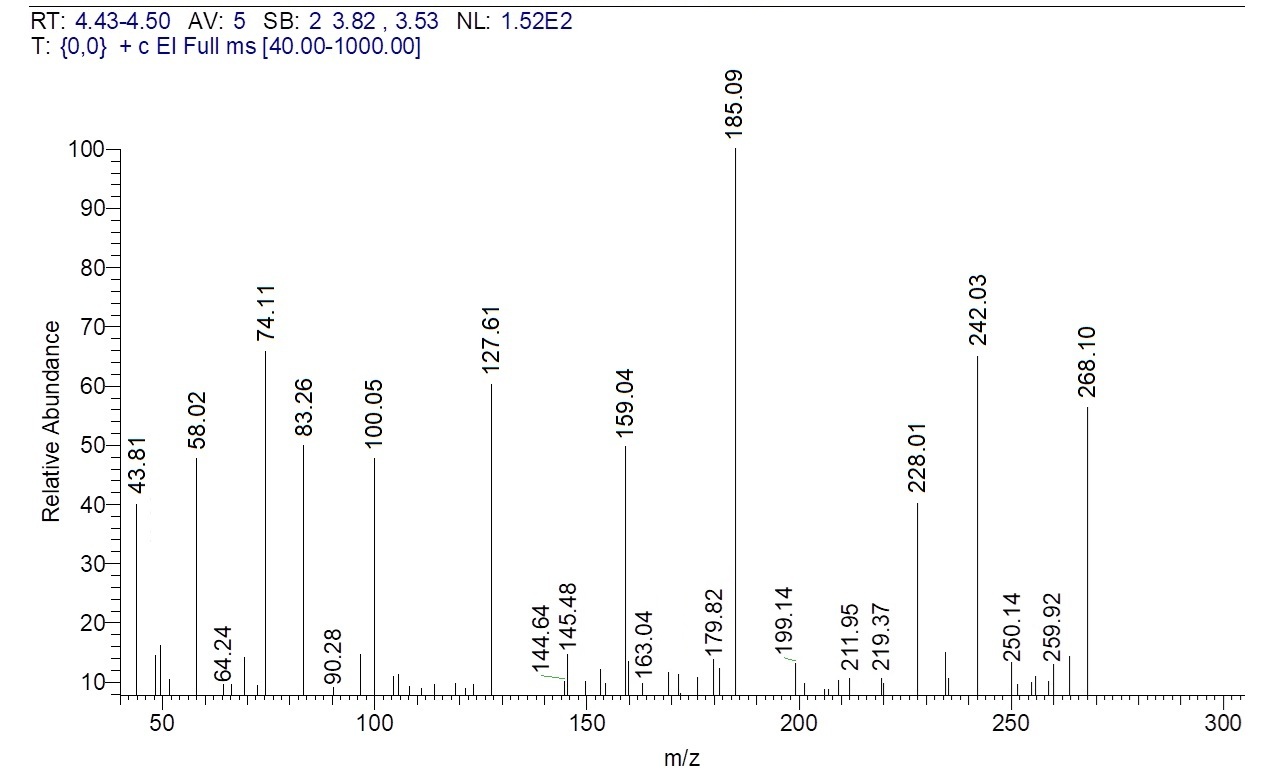
**

**Figure S4.** Mass spectrum of compound **2**

The mass spectrum of compound **2** shows a prominent peak at m/z 268, confirming the molecular weight of the compound.

**
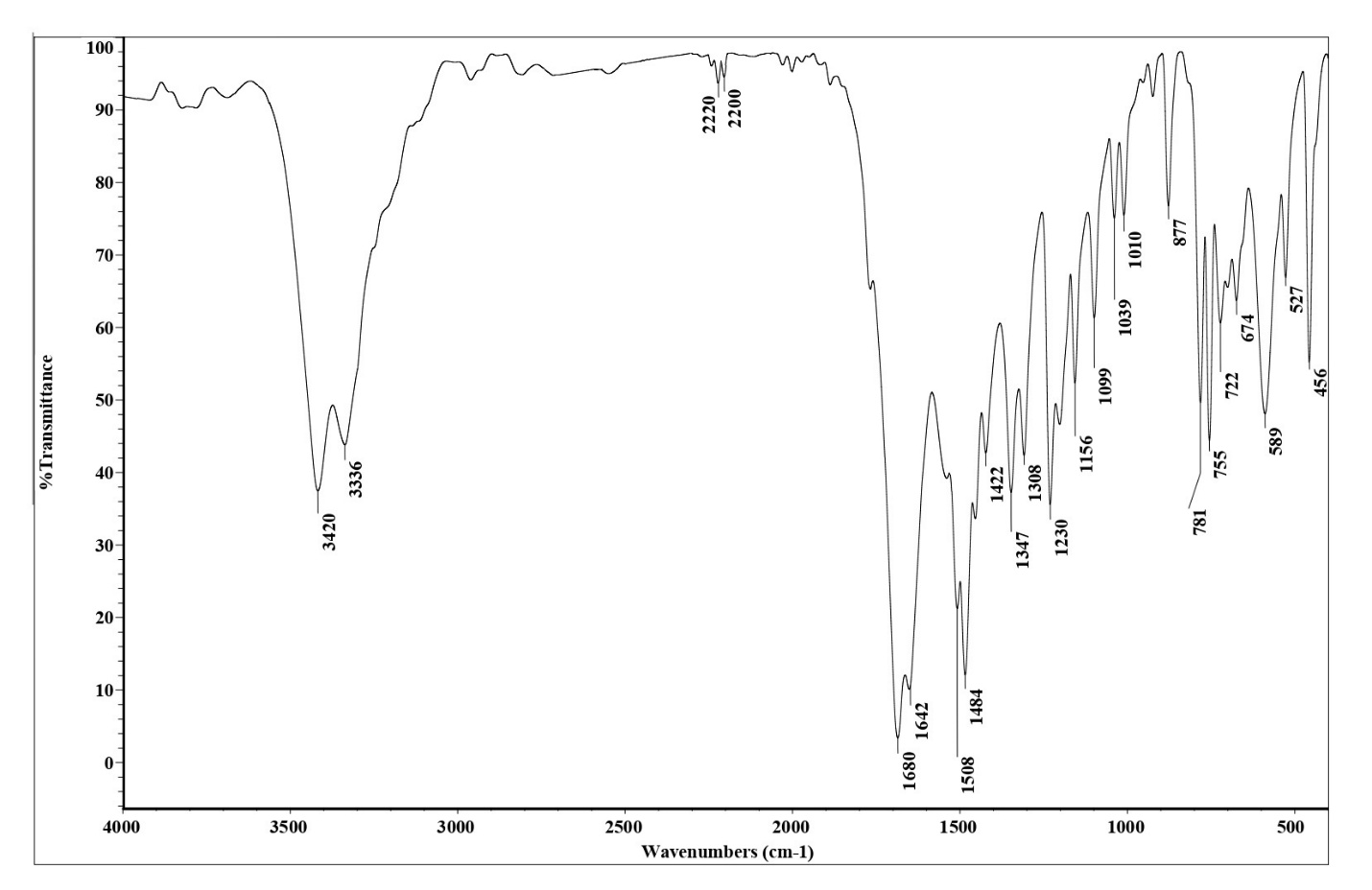
**

**Figure S5.** IR spectrum of compound **4**

IR (KBr): *ν_max_*, cm^-1^: 3420, 3336 (NH_2_), 2220 (CN), 2200 (CN), 1680 (amidic CO), 1642 (C=N) functional groups


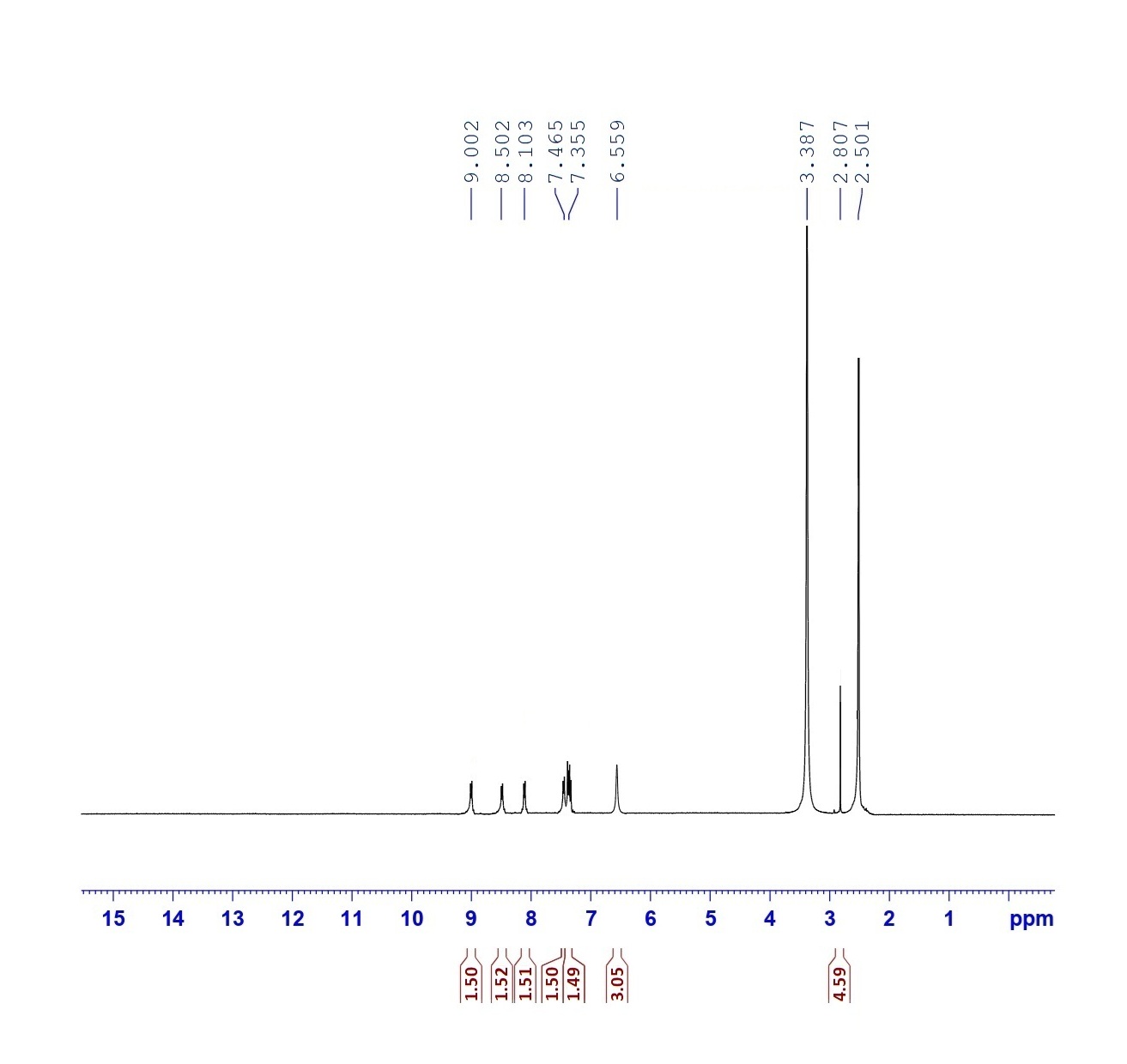


**Figure S6.** ^1^H-NMR of compound **4**

^1^H-NMR (DMSO-*d_6_*) *δ* ppm: 2.80 (s, 3H, CH_3_), 6.55 (s, 2H, NH_2_), 7.35 (m, 1H, pyridine-C_3_H), 7.46 (d, 1H, Ar-H), 8.10 (d, 1H, Ar-H), 8.50 (d, 1H, pyridine-C_4_H), 9.00 (d, 1H, pyridine-C_2_H)

**
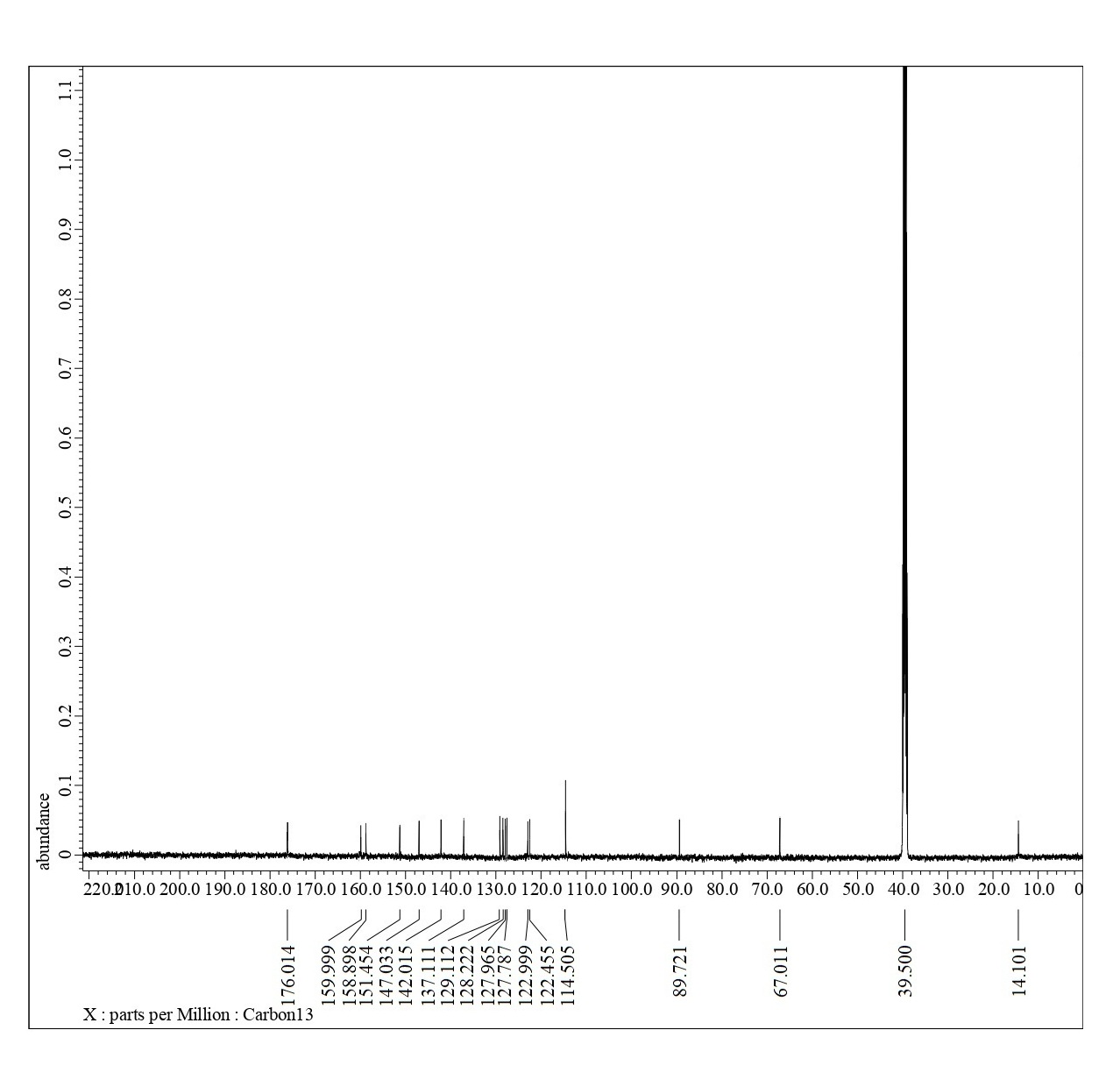
**

**Figure S7.** ^13^C-NMR of Compound **4**

The ^13^C-NMR spectrum shows chemical shifts for the carbon atoms in the pyridine and quinoline ring systems and the cyano and amidic functional groups.


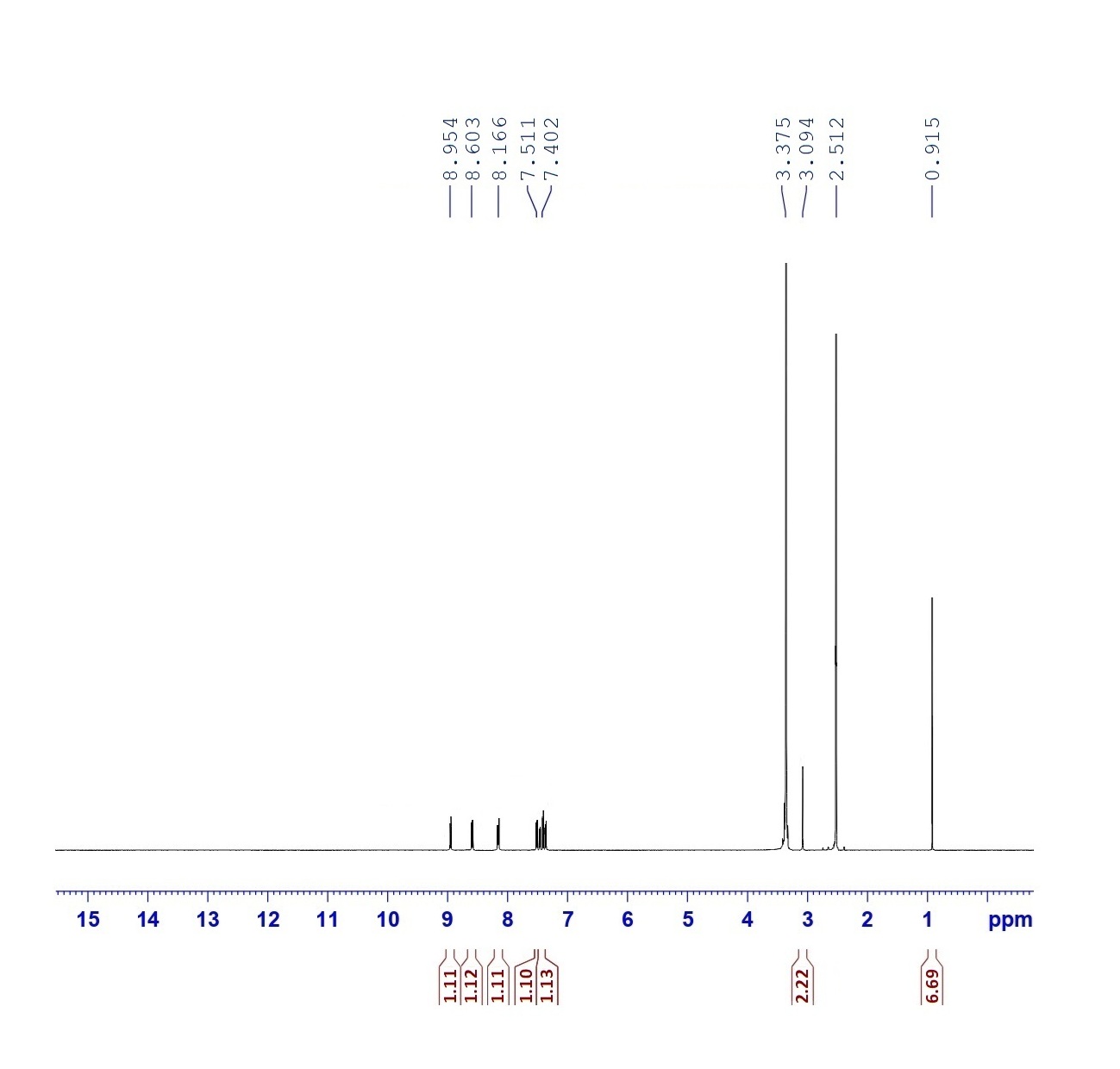


**Figure S8.** ^1^H-NMR of compound **6**

^1^H-NMR (DMSO-*d_6_*) *δ* ppm: 0.91 (s, 6H, 2CH_3_), 3.09 (s, 2H, 2CH), 7.40 (m, 1H, pyridine-C_3_H), 7.51 (d, 1H, Ar-H), 8.16 (d, 1H, Ar-H), 8.60 (d, 1H, pyridine-C_4_H), 8.95 (d, 1H, pyridine-C_2_H)

**
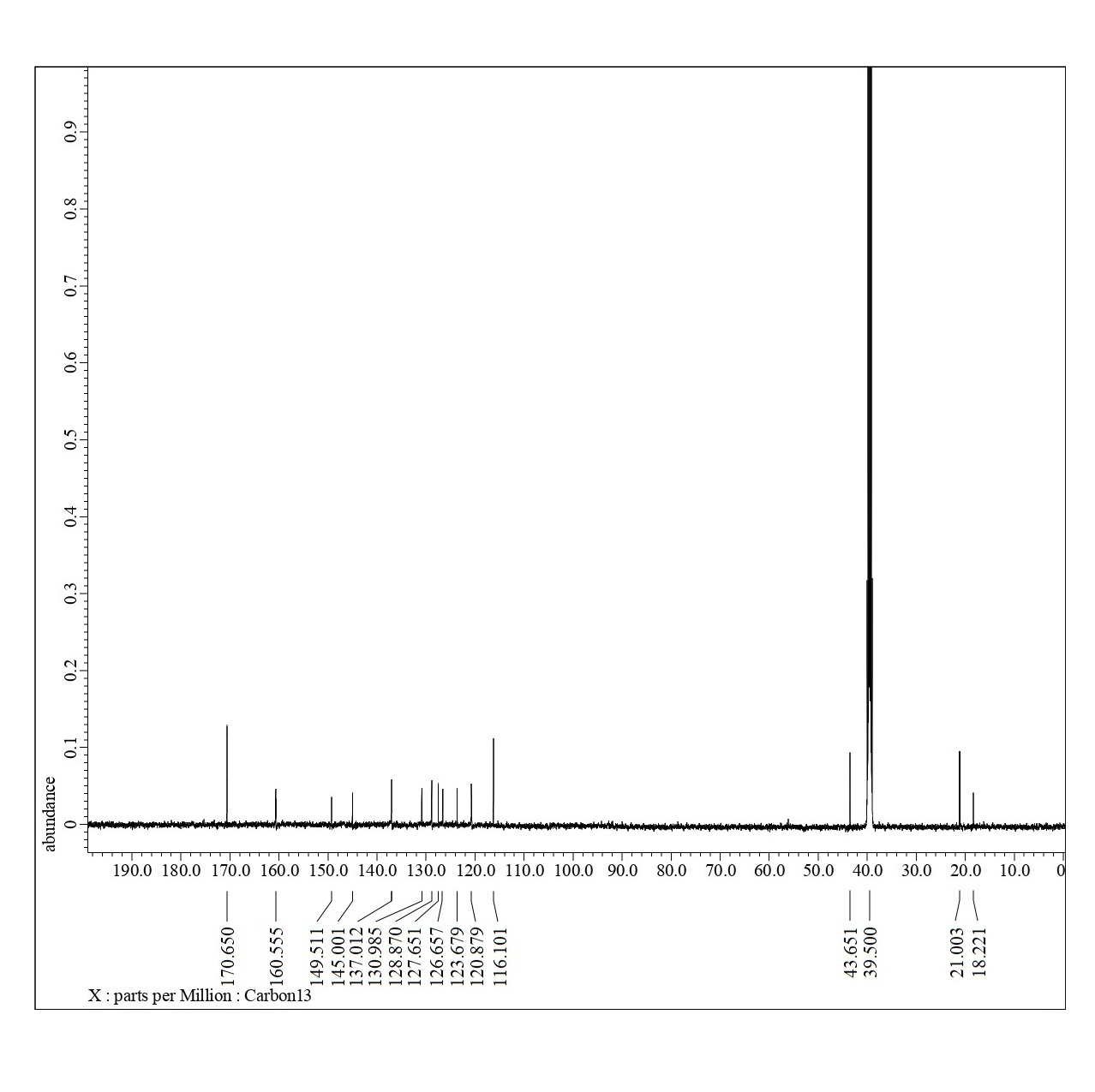
**

**Figure S9.** ^13^C-NMR of Compound **6**

The ^13^C-NMR spectrum shows chemical shifts for the carbon atoms in the pyridine and quinoline ring systems and the cyano and amidic functional groups.

**
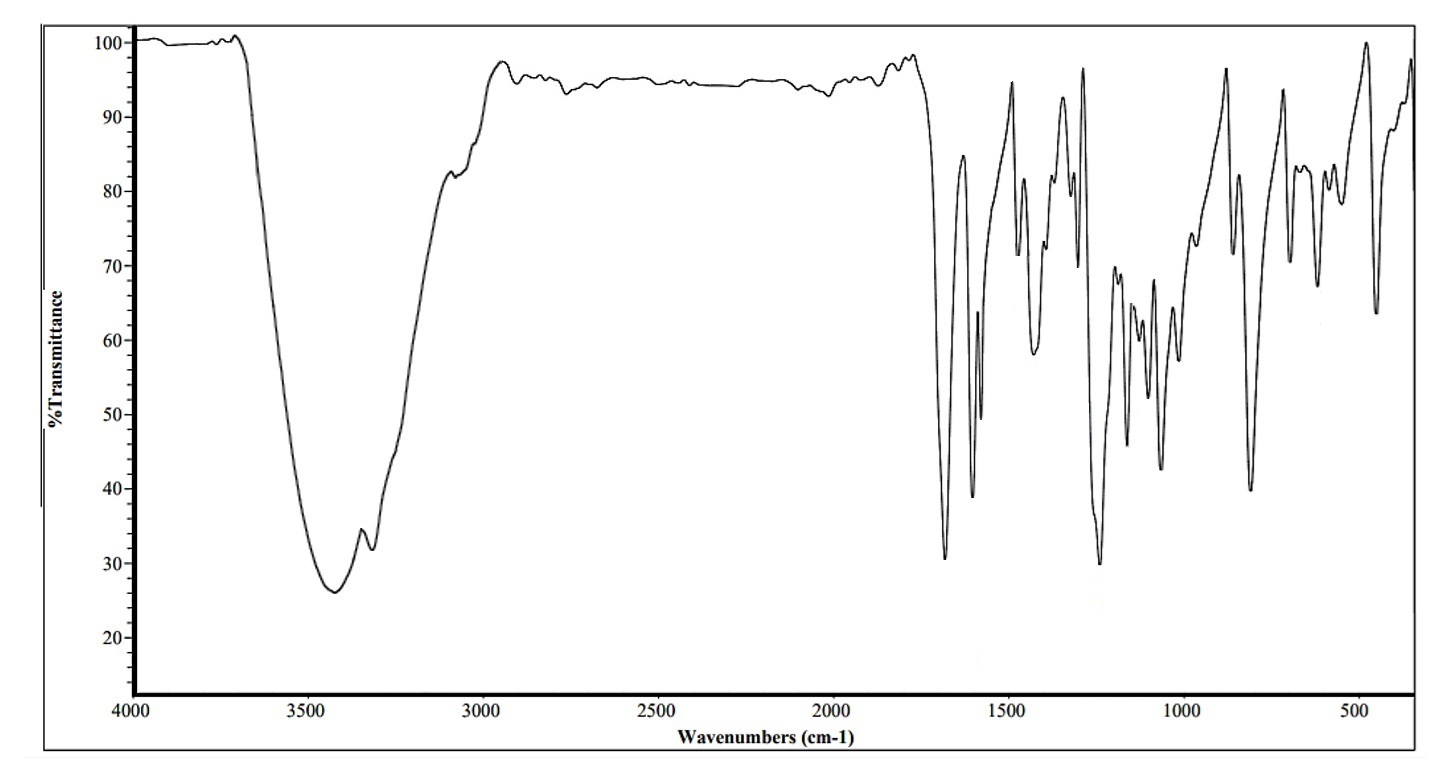
**

**Figure S10.** IR spectrum of compound **7**

IR (KBr): *ν_max_*, cm^-1^: 3410 (NH_2_), 3320 (NH), 1682 (CO), 1150 (C=S) functional groups


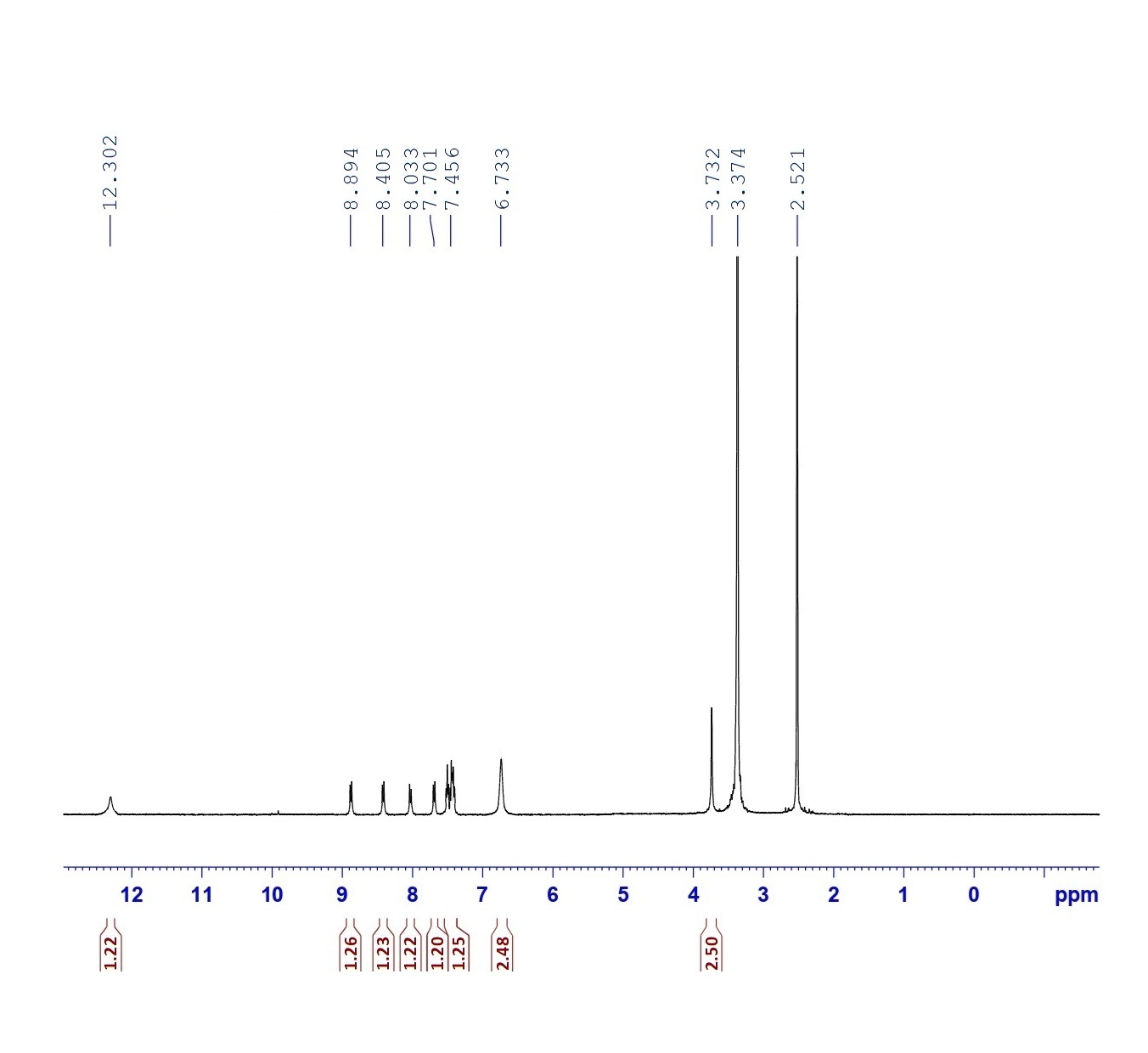


**Figure S11.** ^1^H-NMR of compound **7**

^1^H-NMR (DMSO-*d_6_*) *δ* ppm: 3.73 (s, 2H, CH_2_), 6.73 (s, 2H, NH_2_), 7.45 (m, 1H, pyridine-C_3_H), 7.70 (d, 1H, Ar-H), 8.03 (d, 1H, Ar-H), 8.40 (d, 1H, pyridine-C_4_H), 8.89 (d, 1H, pyridine-C_2_H), 12.30 (s, 1H, NH)

**
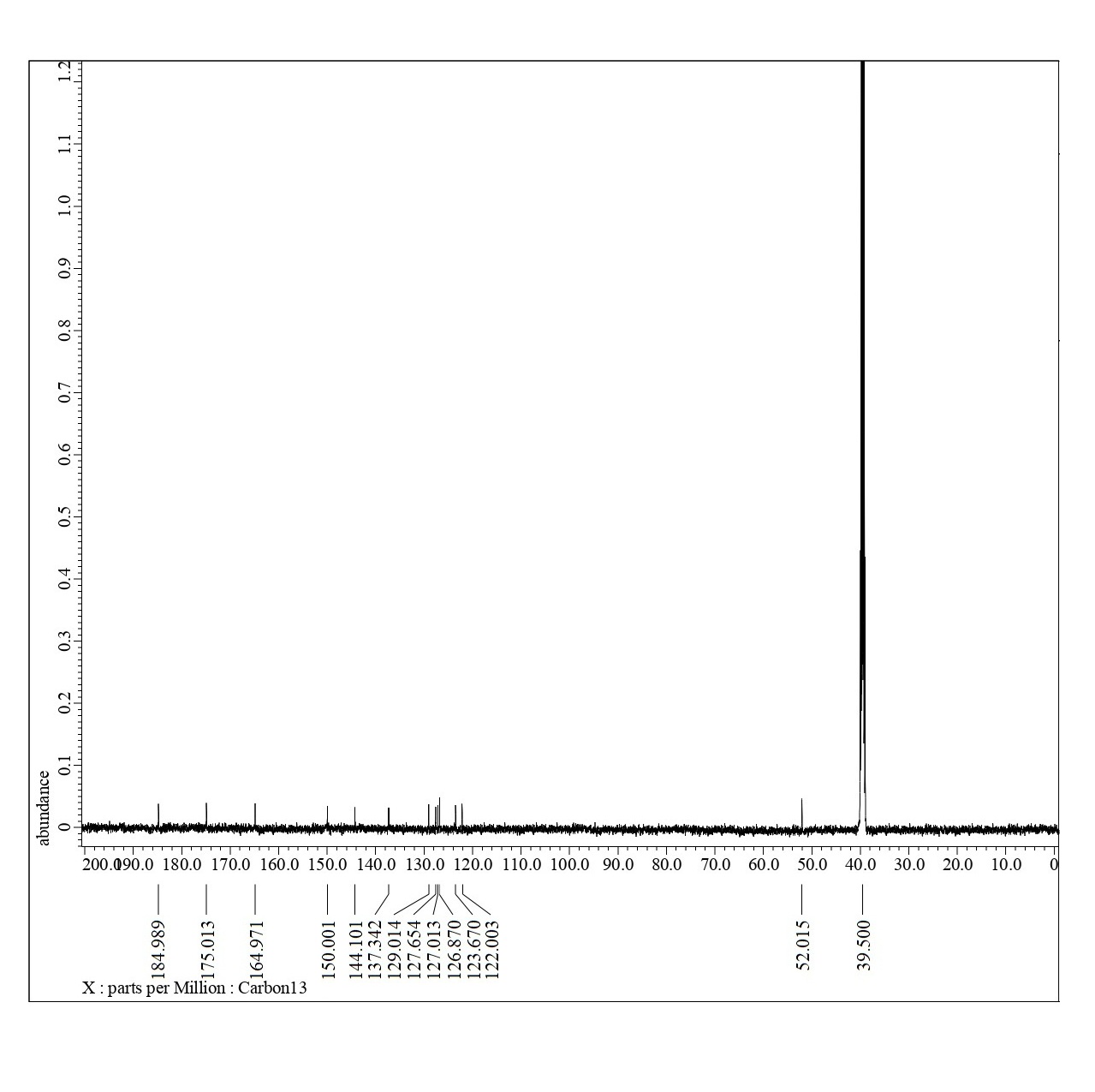
**

**Figure S12.** ^13^C-NMR of Compound **7**

The ^13^C-NMR spectrum shows chemical shifts corresponding to the thiazole and quinoline rings, confirming structural integrity

**
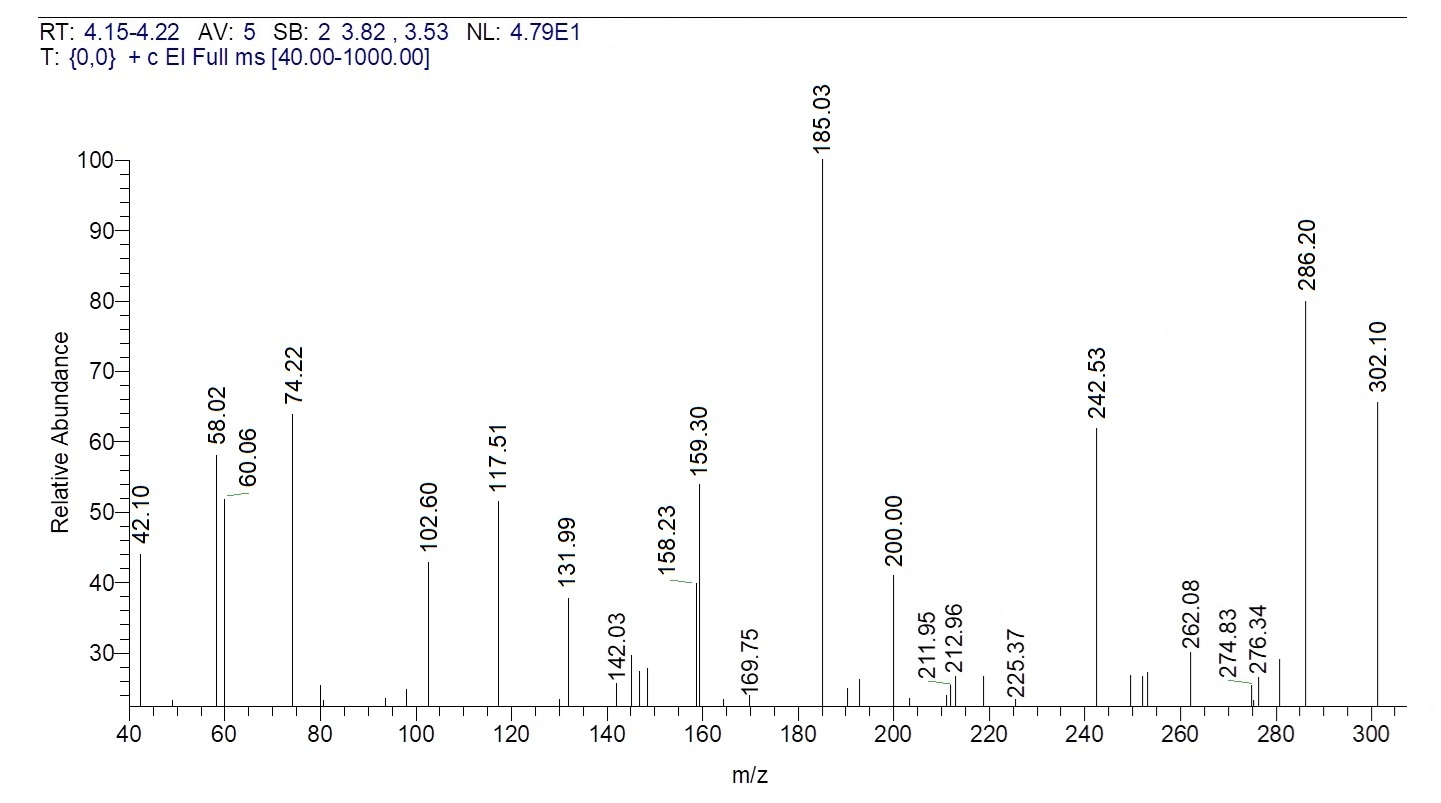
**

**Figure S13.** Mass spectrum of compound **7**

The mass spectrum of compound **7** shows a prominent peak at m/z 302, confirming the molecular weight of the compound.

**
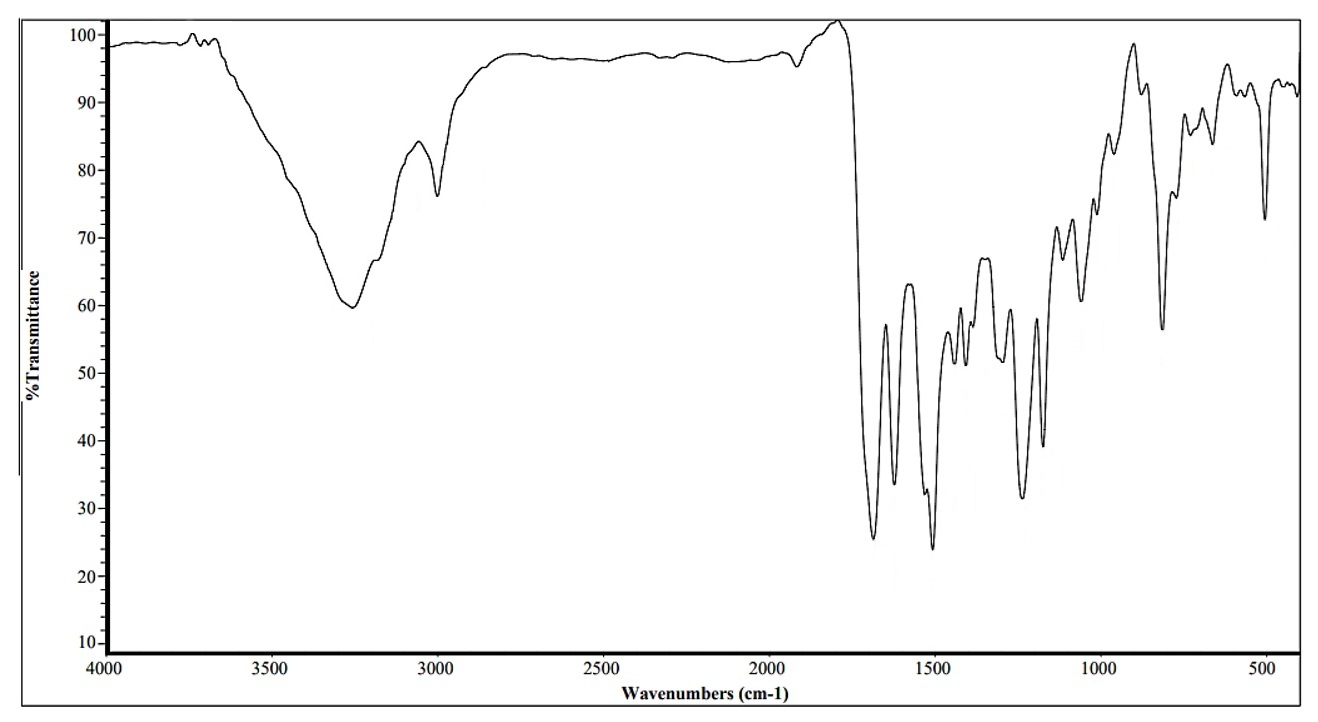
**

**Figure S14.** IR spectrum of compound **8**

IR (KBr): *ν_max_*, cm^-1^: 3230 (NH), 1675 (CO), 1618 (C=N) functional groups

**
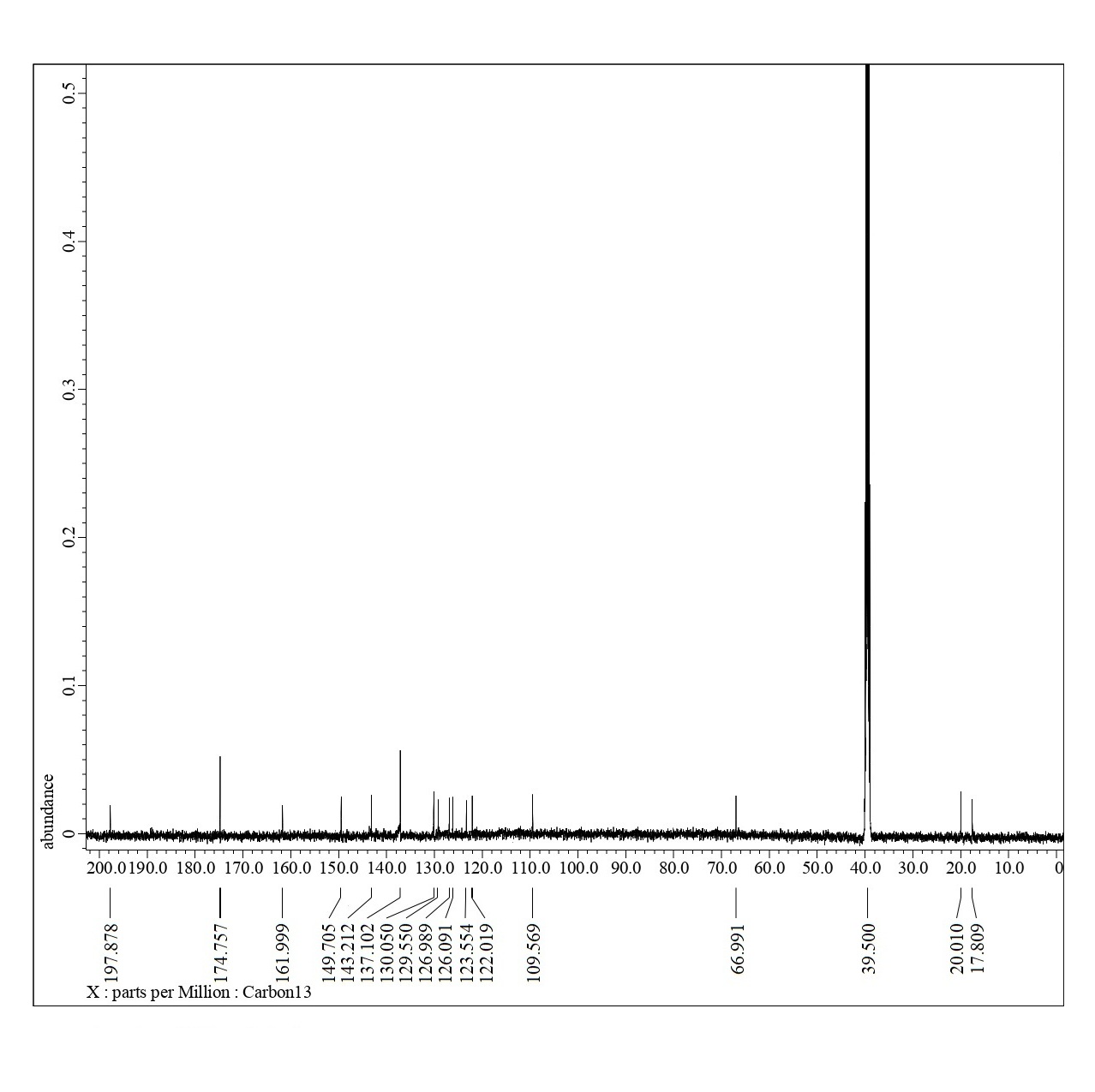
**

**Figure S15.** ^13^C-NMR of Compound **8**

^13^C-NMR spectrum displays signals for CH_3_ groups, pyridine, and carbonyl carbons confirming compound substitution.

**
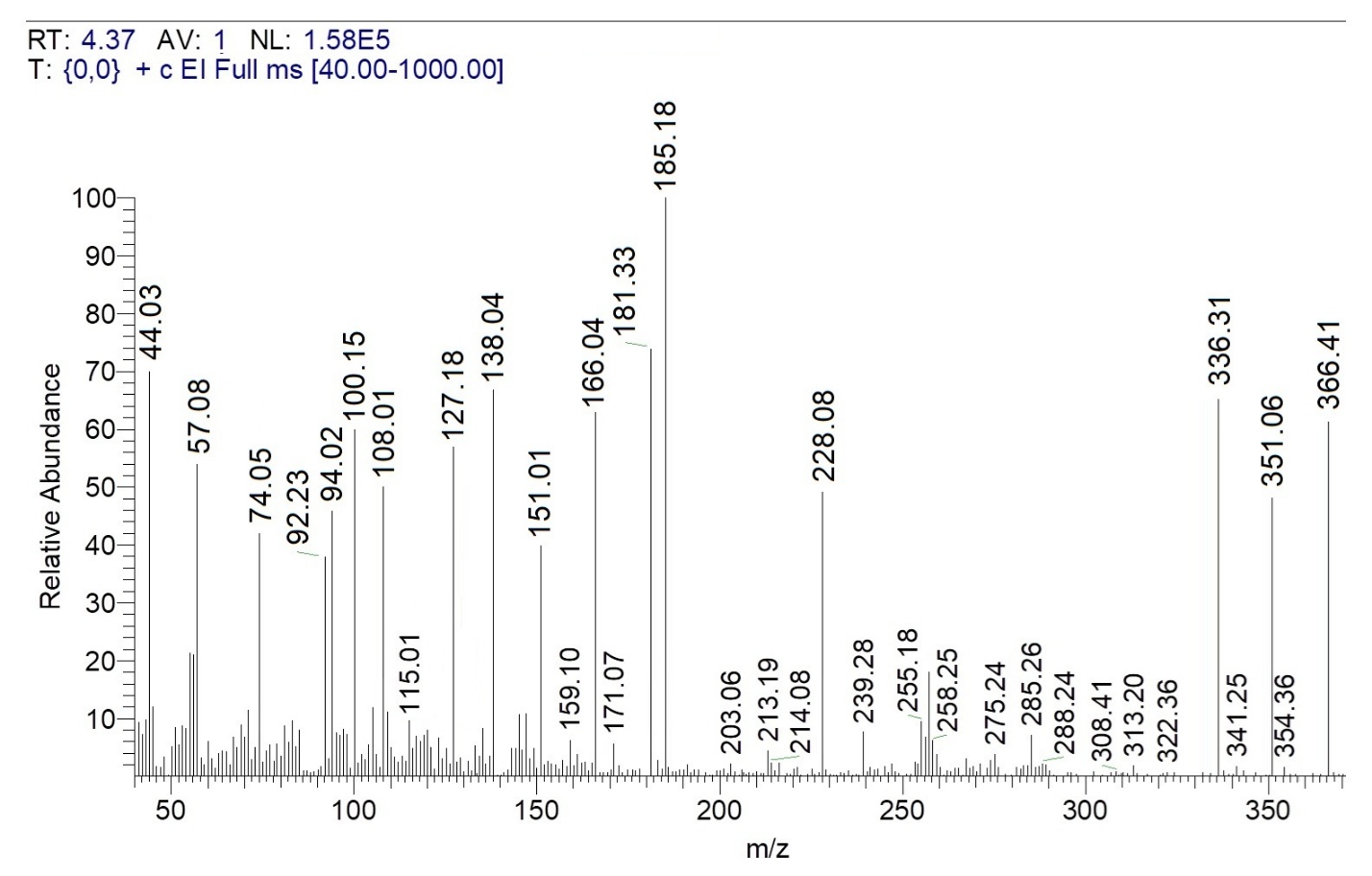
**

**Figure S16.** Mass spectrum of compound **8**

The mass spectrum of compound **8** shows a prominent peak at m/z 366, confirming the molecular weight of the compound.

**
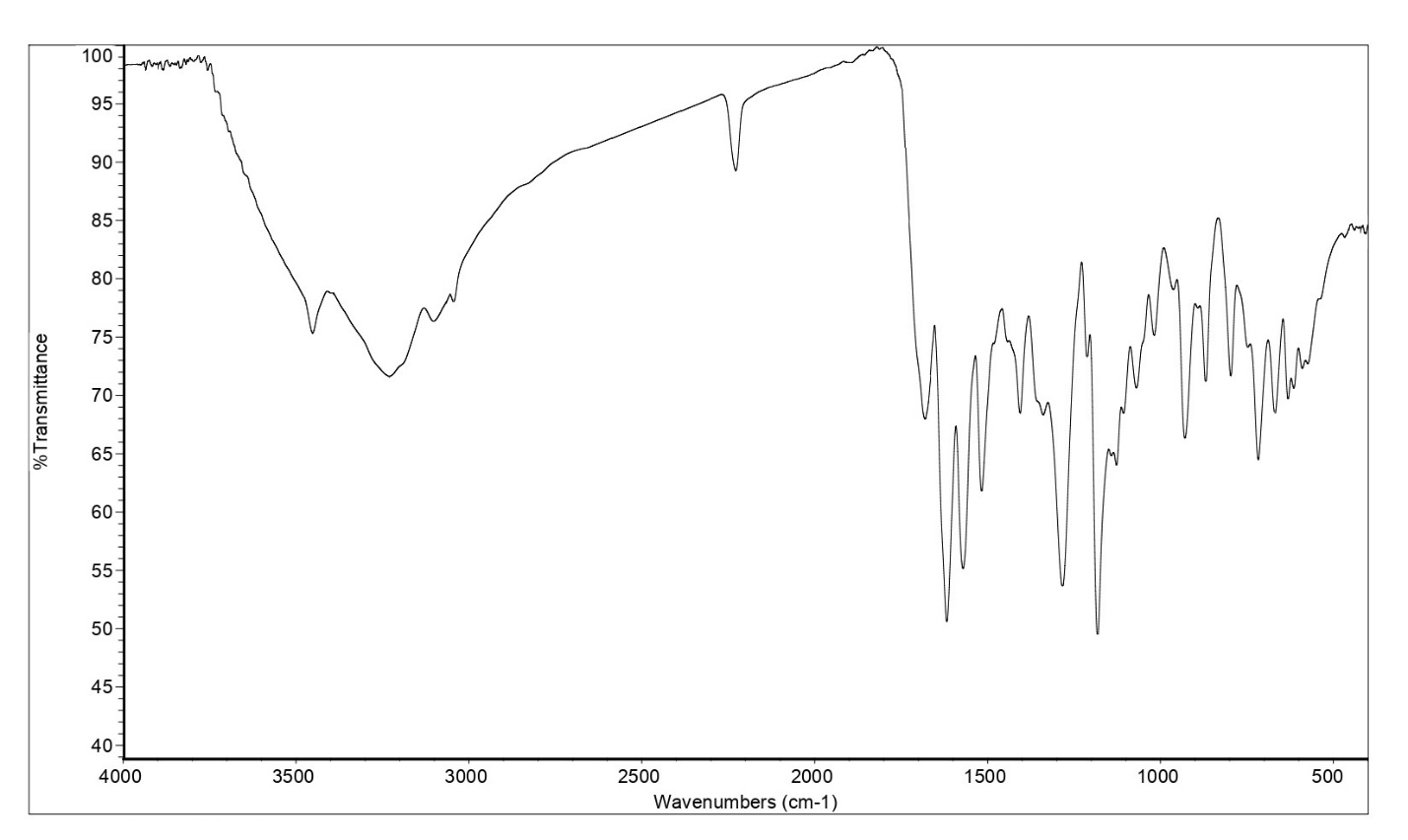
**

**Figure S17.** IR spectrum of compound **10**

IR (KBr): *ν_max_*, cm^-1^: 3450 (NH_2_), 3235 (NH), 2219 (CN), 1680 (CO), 1618 (C=N) functional groups

**
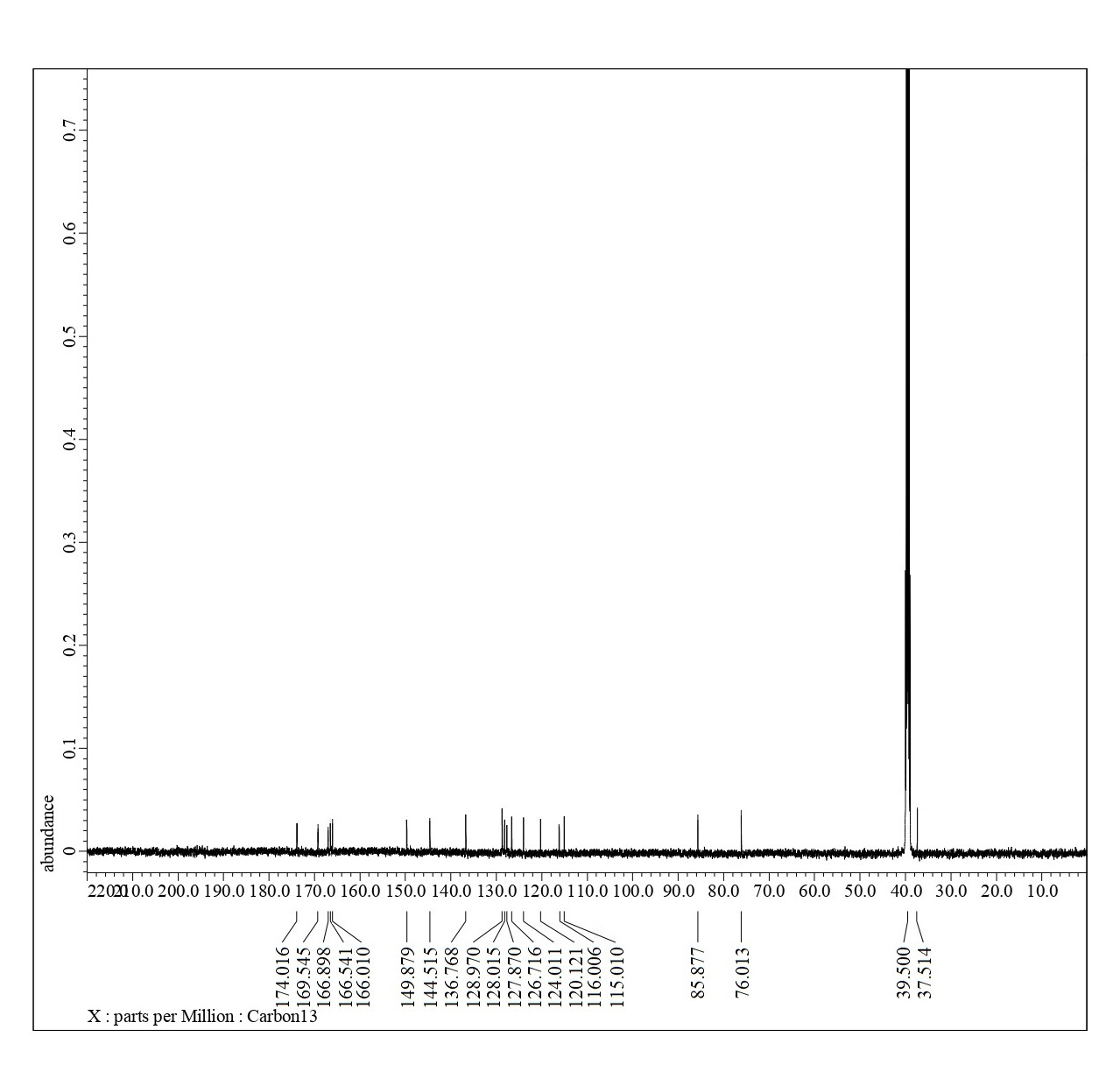
**

**Figure S18.** ^13^C-NMR of Compound **10**

Chemical shifts support the structure of substituted pyridine and quinoline rings.

**
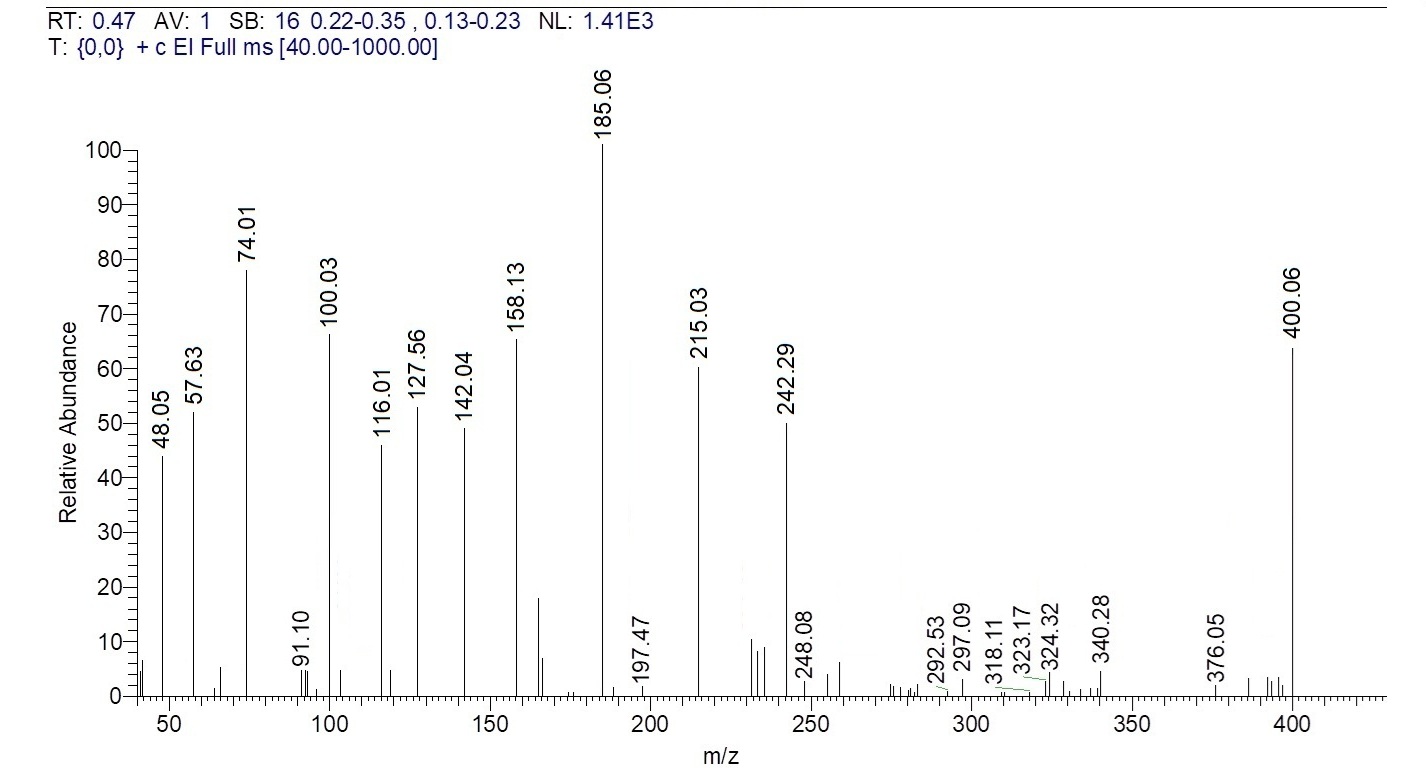
**

**Figure S19.** Mass spectrum of compound **10**

The mass spectrum of compound **10** shows a prominent peak at m/z 400, confirming the molecular weight of the compound.

**
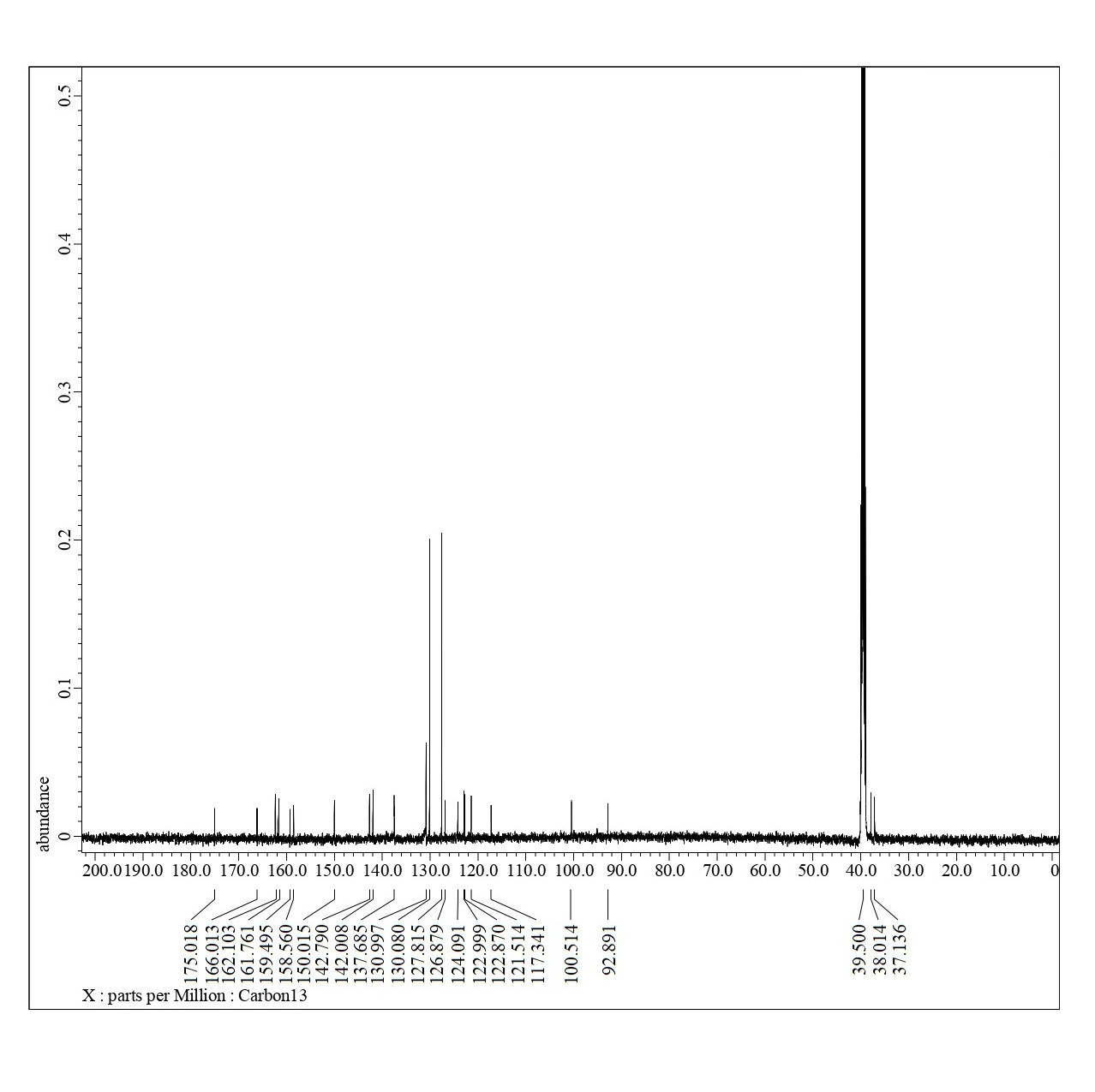
**

**Figure S20.** ^13^C-NMR of Compound **12**

Signals confirm the presence of pyrano[2,3-d]pyrimidine fused to quinoline-thiazole moiety.

**
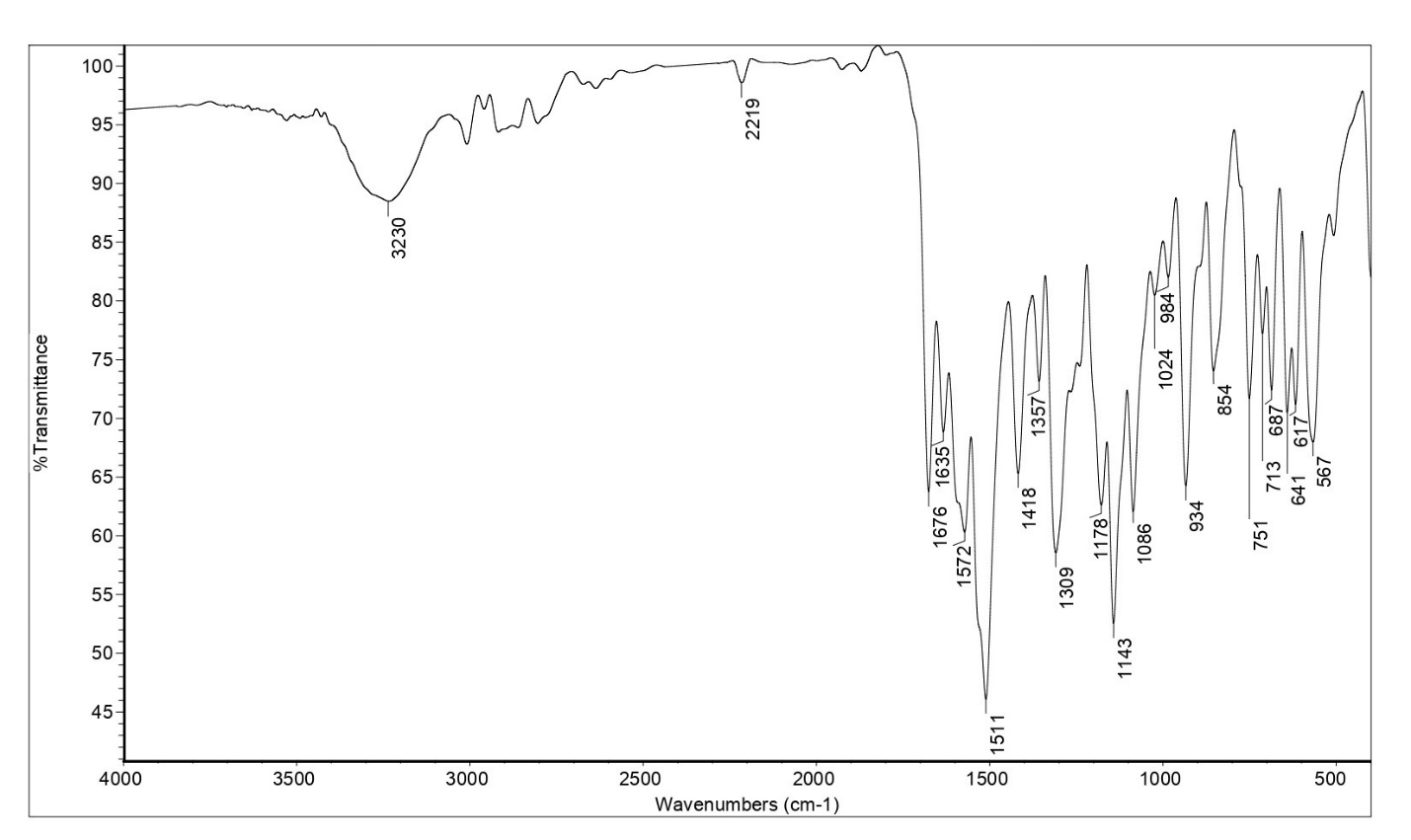
**

**Figure S21.** IR spectrum of compound **13**

IR (KBr): *ν_max_*, cm^-1^: 3230 (NH), 2219 (CN), 1676 (CO), 1635 (C=N) functional groups


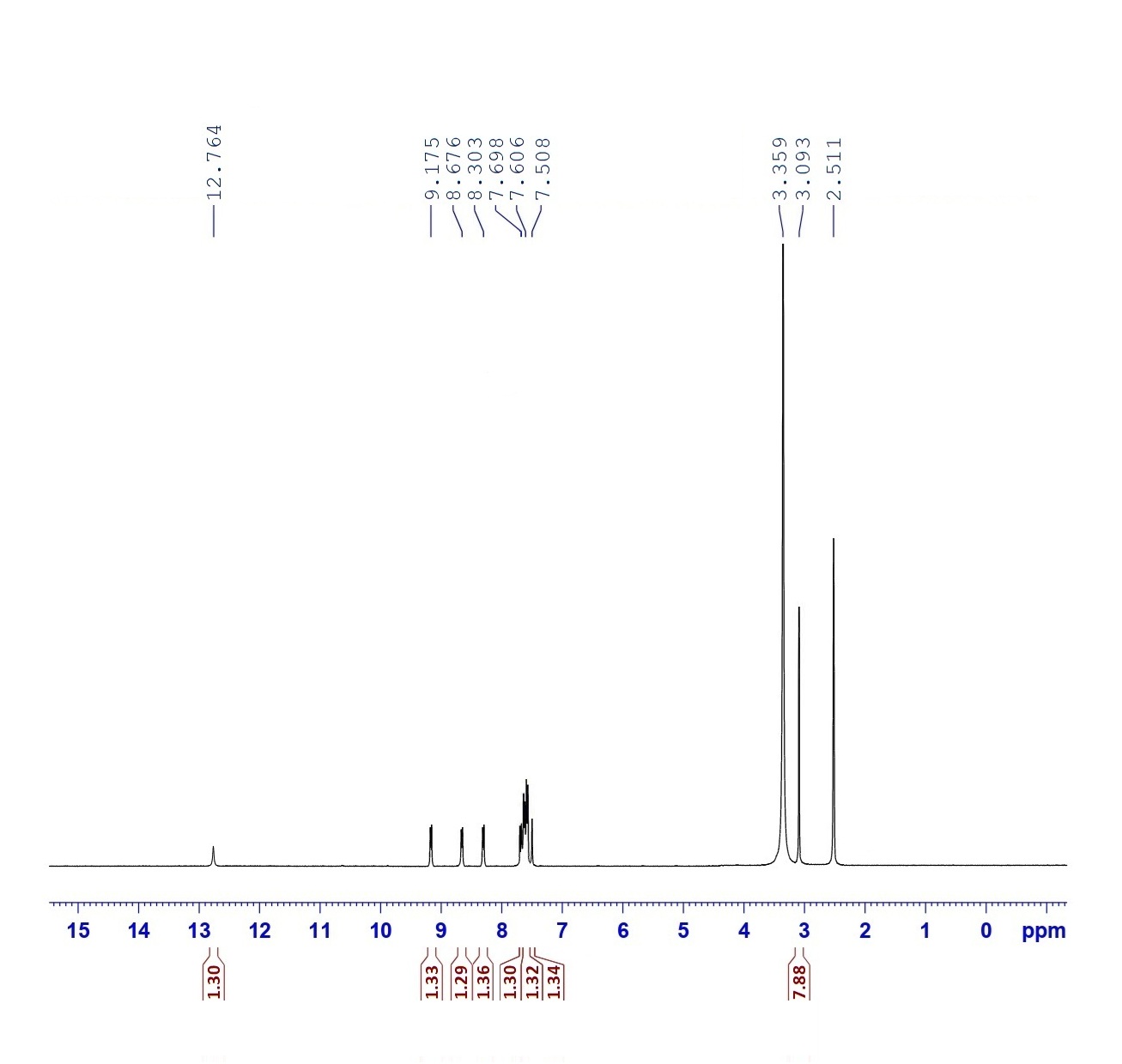


**Figure S22.** ^1^H-NMR of compound **13**

^1^H-NMR (DMSO-*d_6_*) *δ* ppm: 3.09 (s, 6H, 2CH_3_), 7.50 (s, 1H, olefinic-CH), 7.60 (m, 1H, pyridine-C_3_H), 7.69 (d, 1H, Ar-H), 8.30 (d, 1H, Ar-H), 8.67 (d, 1H, pyridine-C_4_H), 9.17 (d, 1H, pyridine-C_2_H), 12.76 (s, 1H, NH)

**
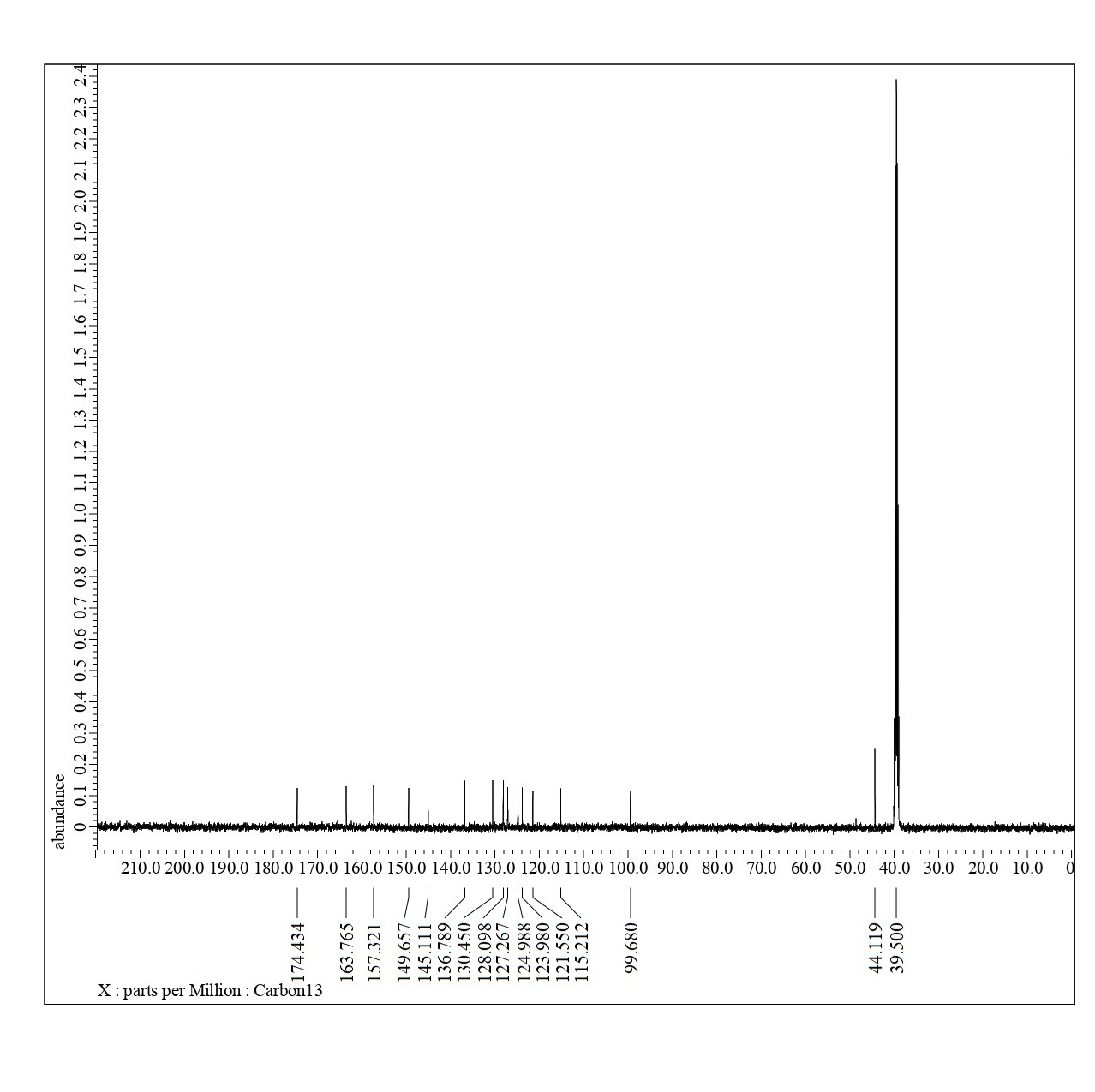
**

**Figure S23.** ^13^C-NMR of Compound **13**

Carbon shifts correspond to dimethylamino and cyanoacrylamide functionalities.

**
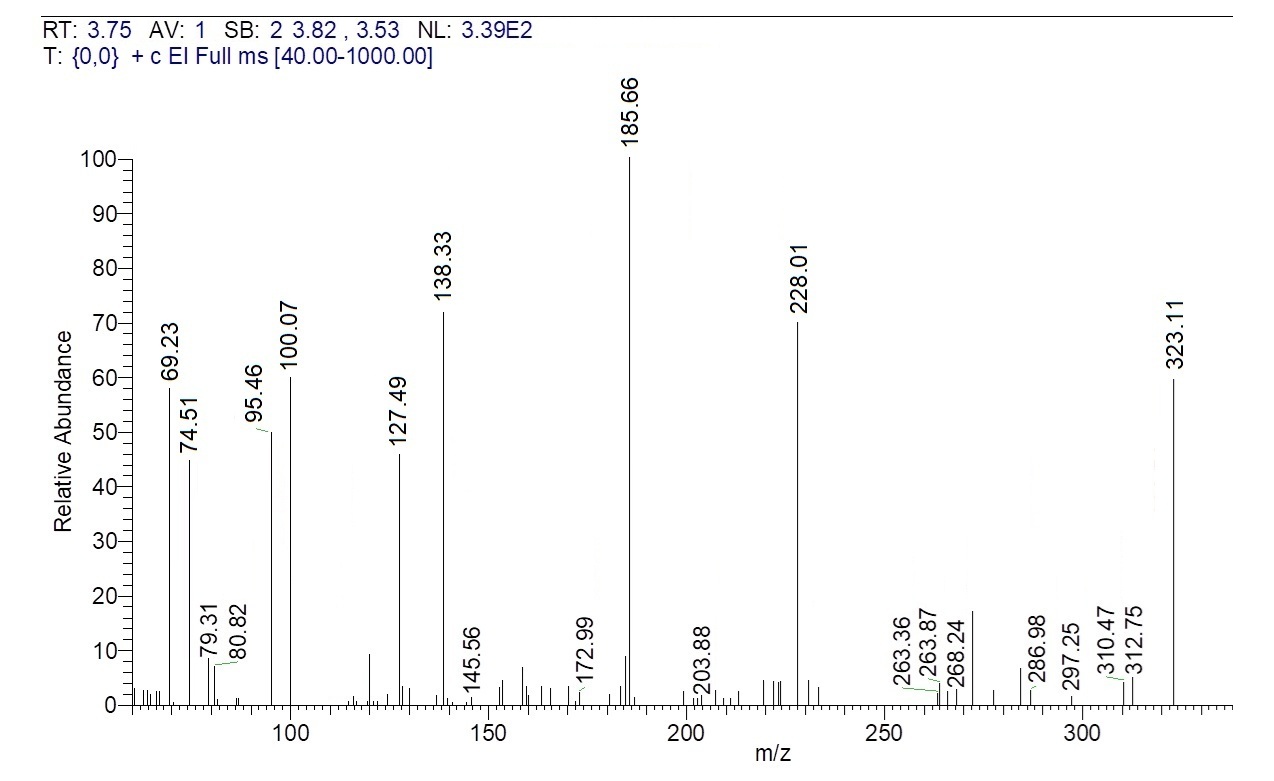
**

**Figure S24.** Mass spectrum of compound **13**

The mass spectrum of compound **13** shows a prominent peak at m/z 323, confirming the molecular weight of the compound.

**
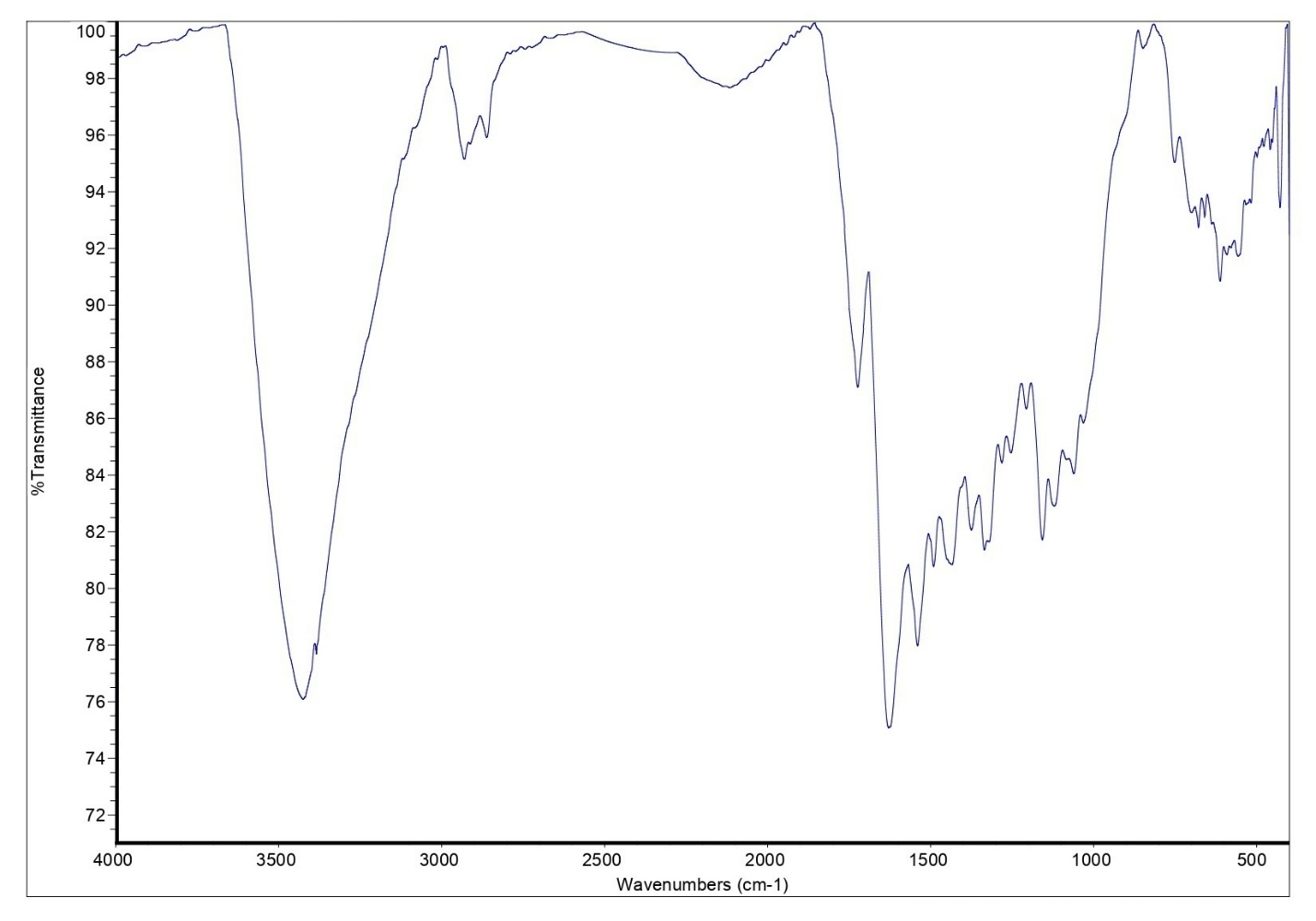
**

**Figure S25.** IR spectrum of compound **14**

IR (KBr): *ν_max_*, cm^-1^: 3420 (NH_2_), 3320 (NH), 1710 (CO) functional groups


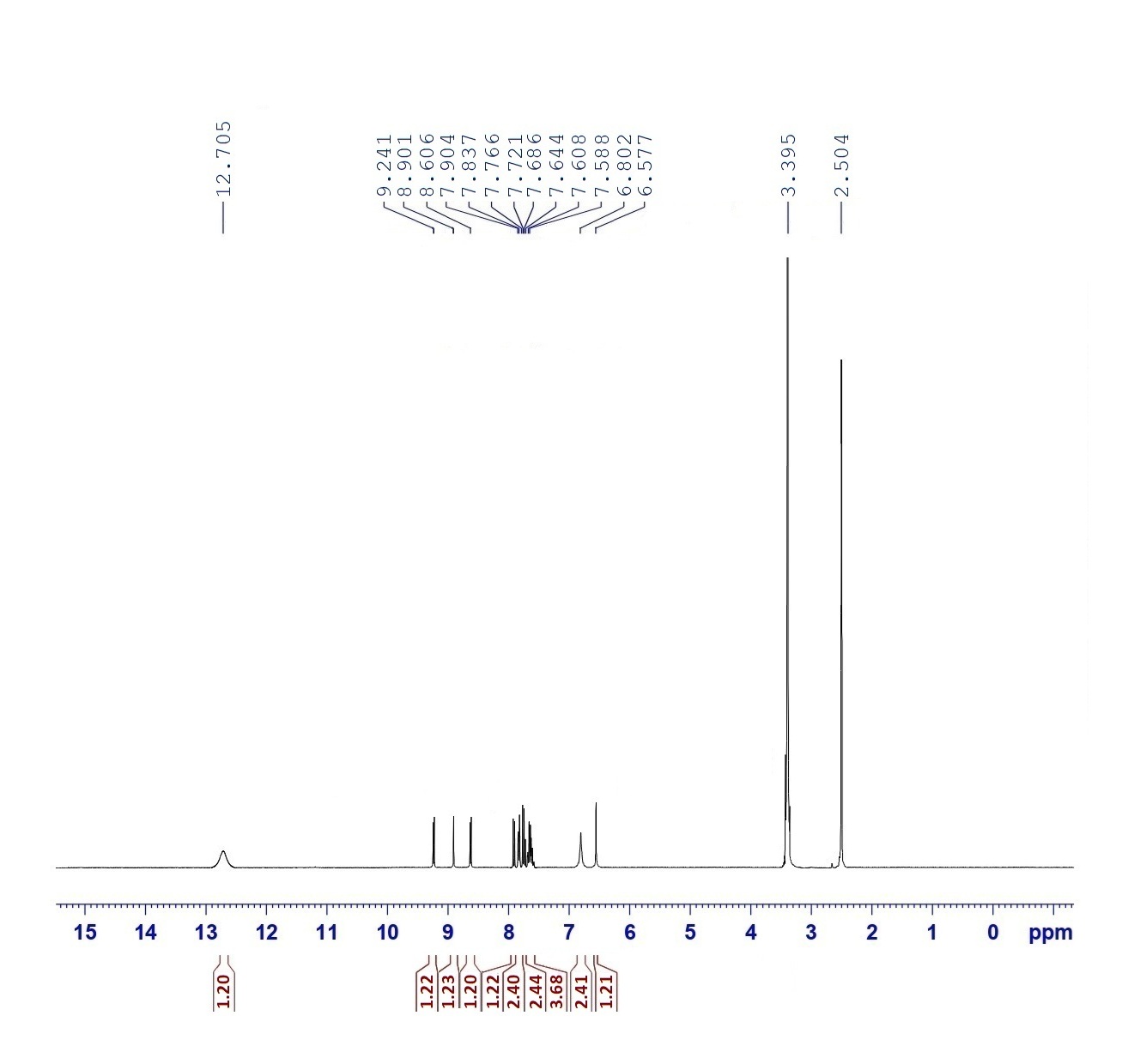


**Figure S26.** ^1^H-NMR of compound **14**

^1^H-NMR (DMSO-*d_6_*) *δ* ppm: 6.57 (s, 1H, pyrazole-CH), 6.80 (s, 2H, NH_2_), 7.58-7.90 (m, 8H, Ar-H + pyridine-C_3_H), 8.60 (d, 1H, pyridine-C_4_H), 8.90 (s, 1H, pyrimidine-CH), 9.24 (d, 1H, pyridine-C_2_H), 12.70 (s, 1H, NH)

**
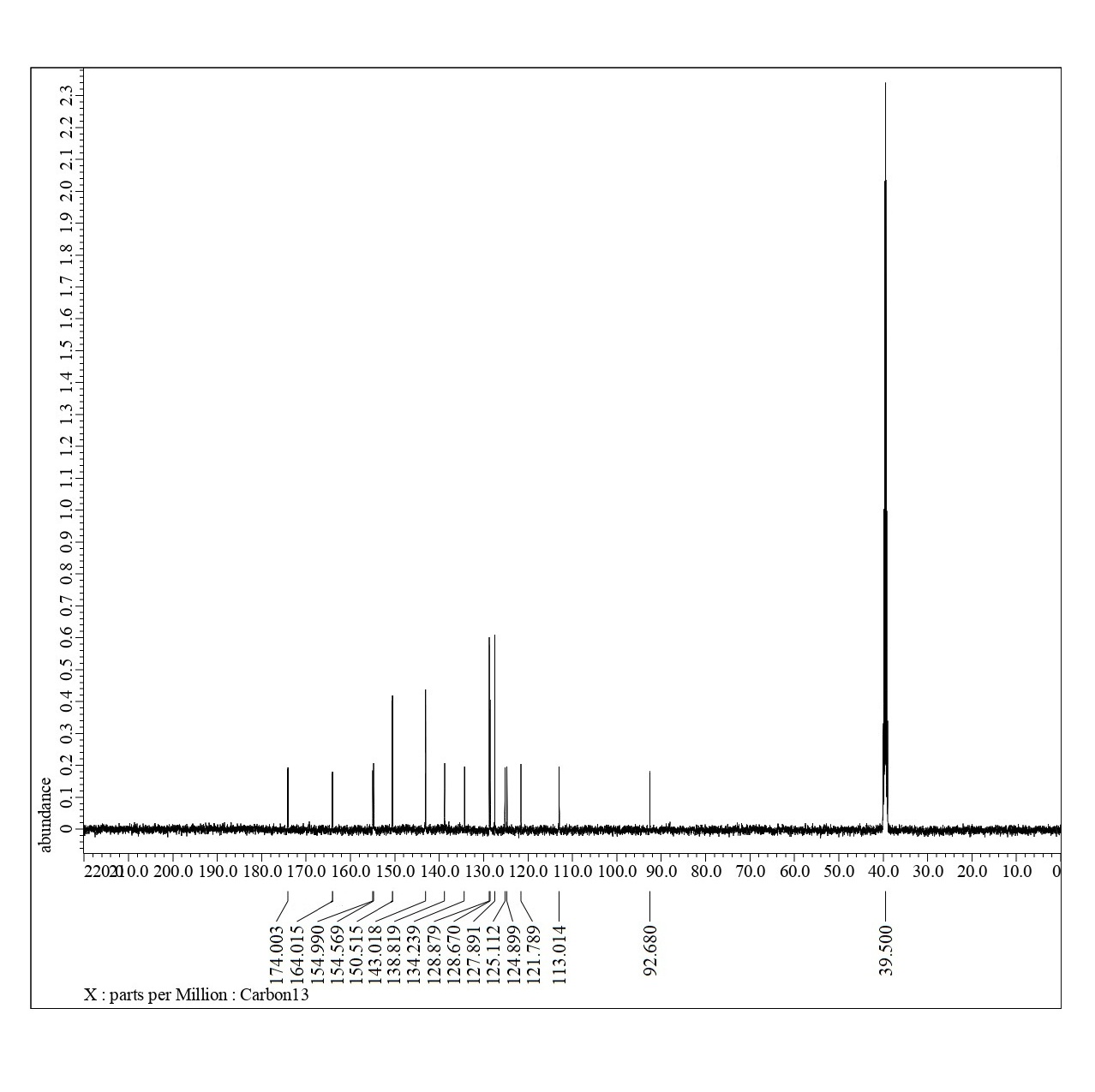
**

**Figure S27.** ^13^C-NMR of Compound **14**

Carbon shifts confirm presence of pyrazolo[1,5-a]pyrimidine framework.


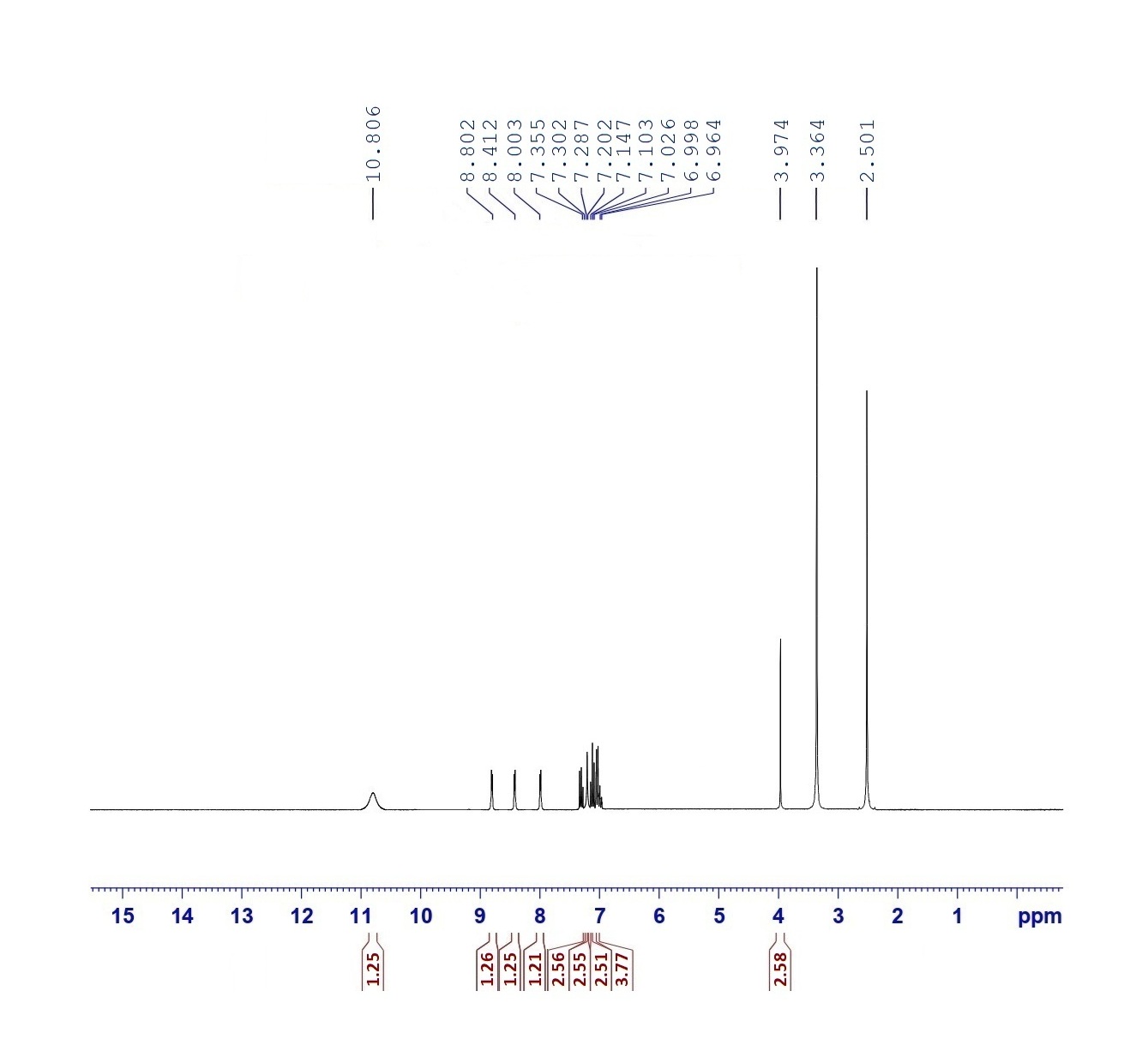


**Figure S28.** ^1^H-NMR of compound **16**

^1^H-NMR (DMSO-*d_6_*) *δ* ppm: 3.97 (s, 2H, CH_2_), 6.96-7.35 (m, 9H, Ar-H + NH_2_ + pyridine-C_3_H), 8.00 (d, 1H, Ar-H), 8.41 (d, 1H, pyridine-C_4_H), 8.80 (d, 1H, pyridine-C_2_H), 10.80 (s, 1H, NH)

**
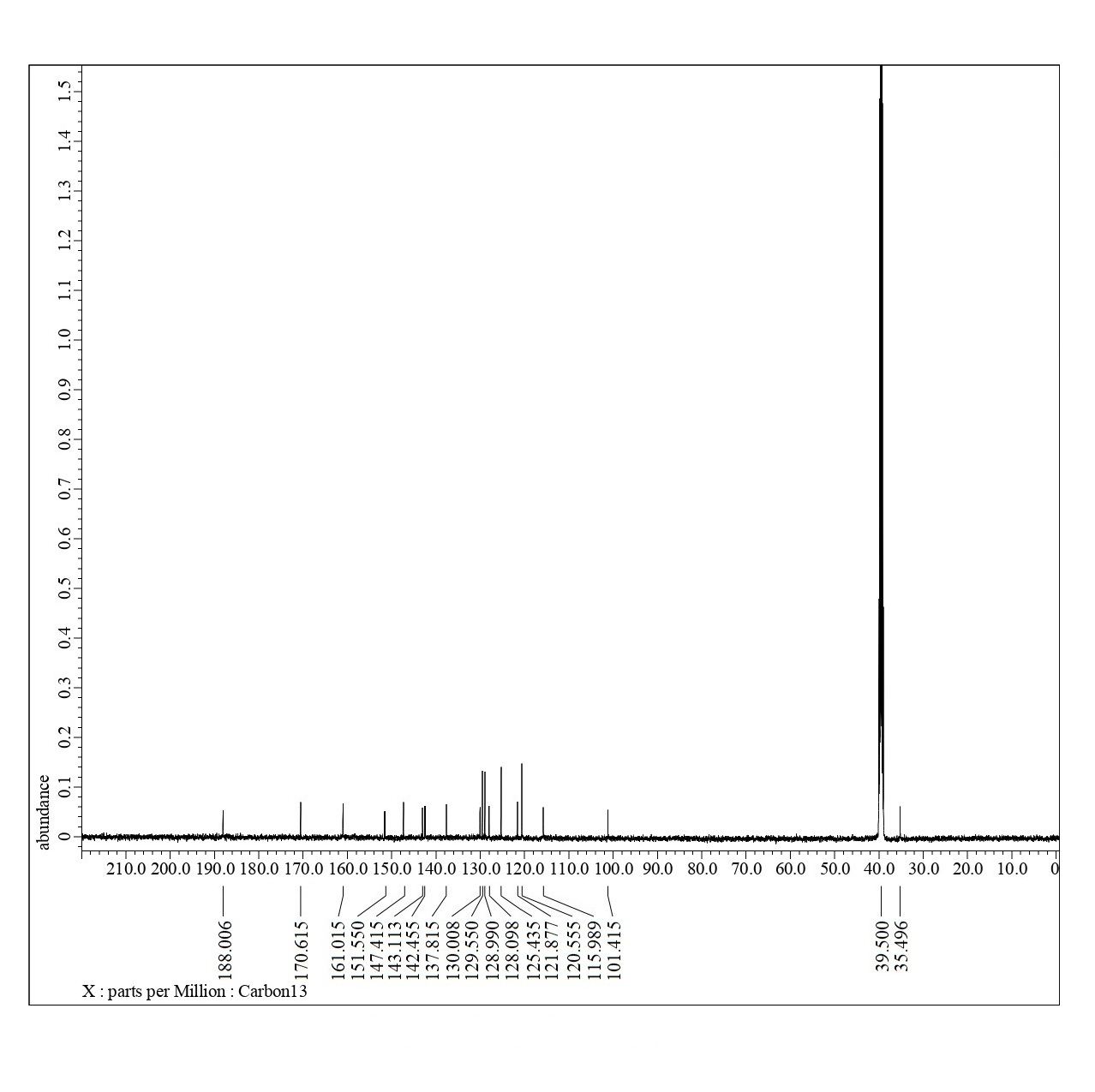
**

**Figure S29.** ^13^C-NMR of Compound **16**

Confirms presence of cyano, phenylamino, and carbonyl functionalities.

**
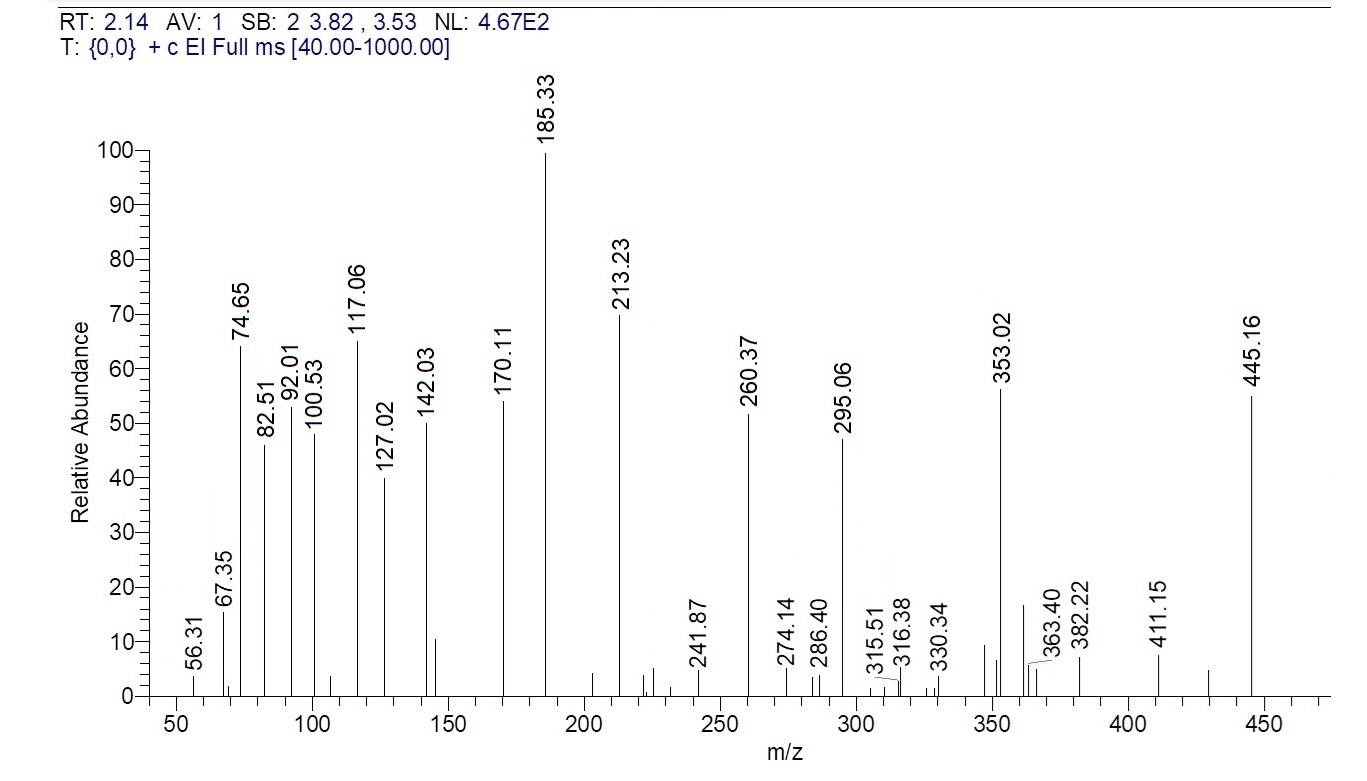
**

**Figure S30.** Mass spectrum of compound **16**

The mass spectrum of compound **16** shows a prominent peak at m/z 445, confirming the molecular weight of the compound.


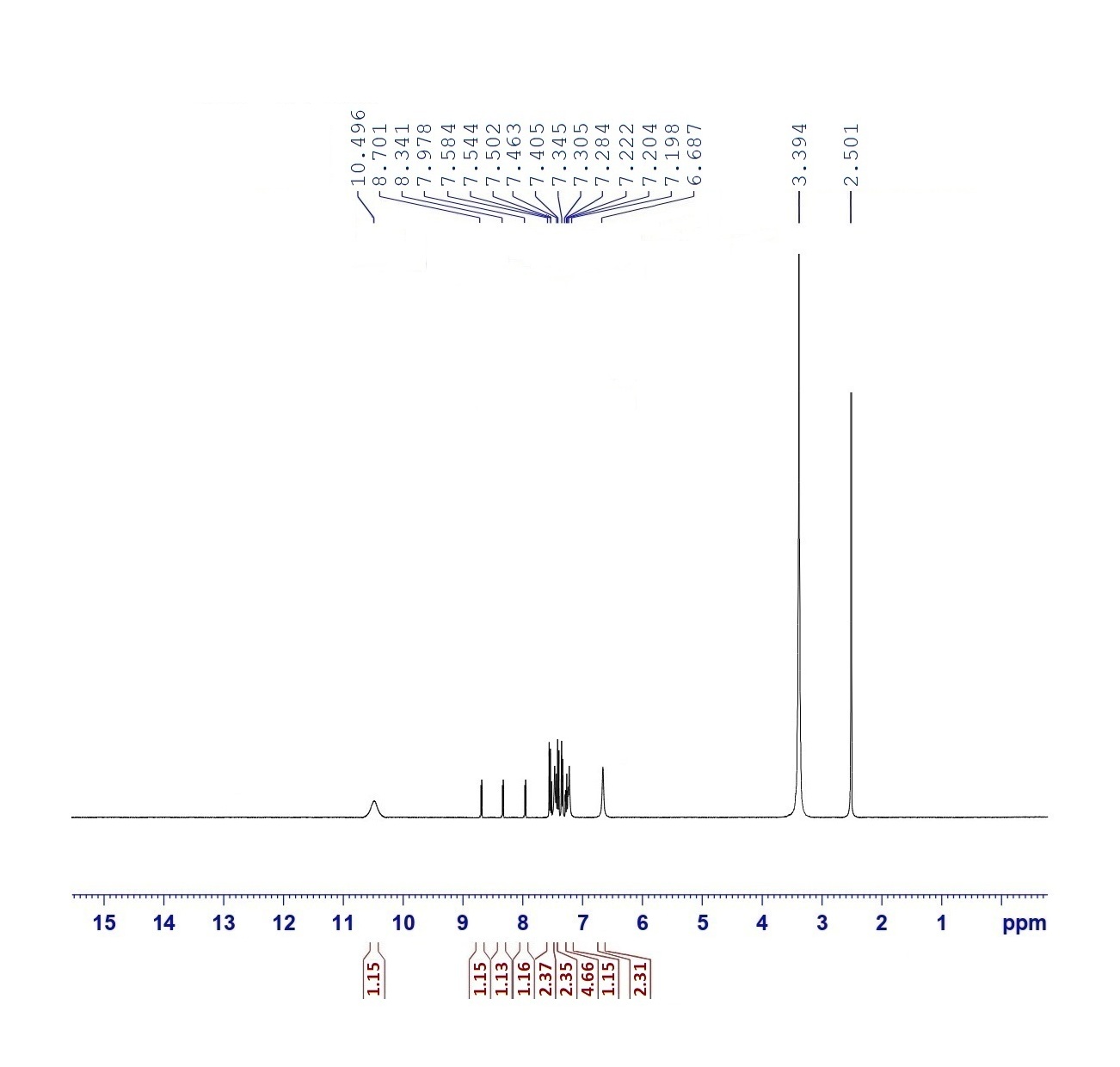


**Figure S31.** ^1^H-NMR of compound **17**

^1^H-NMR (DMSO-*d_6_*) *δ* ppm: 6.68 (s, 2H, CO-NH_2_), 7.19-7.58 (m, 9H, Ar-H + NH_2_ + pyridine-C_3_H), 7.97 (d, 1H, Ar-H), 8.34 (d, 1H, pyridine-C_4_H), 8.70 (d, 1H, pyridine-C_2_H), 10.49 (s, 1H, NH)

**
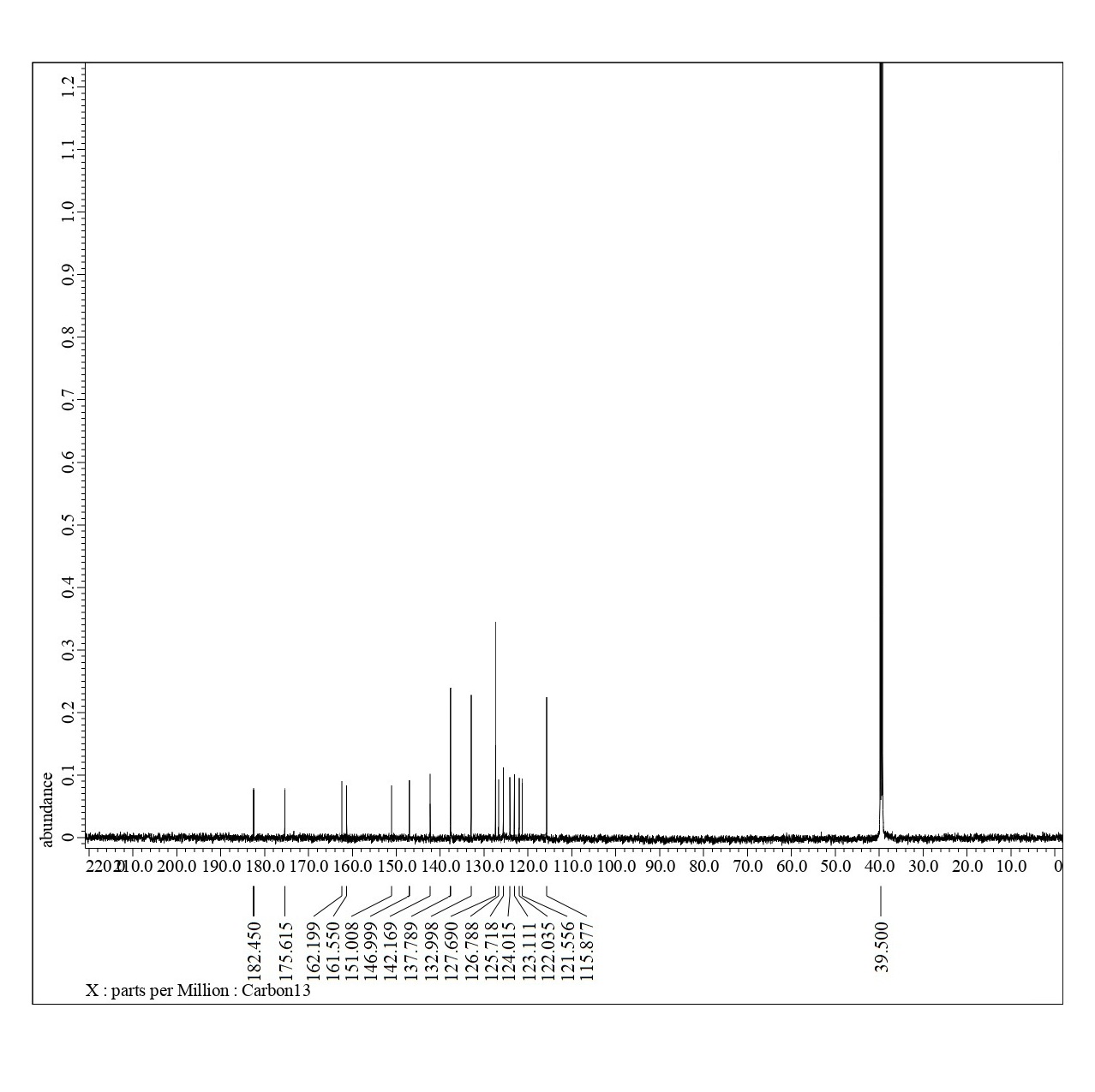
**

**Figure S32.** ^13^C-NMR of Compound **17**

Carbon signals confirm thiophene ring and other structural elements.

**
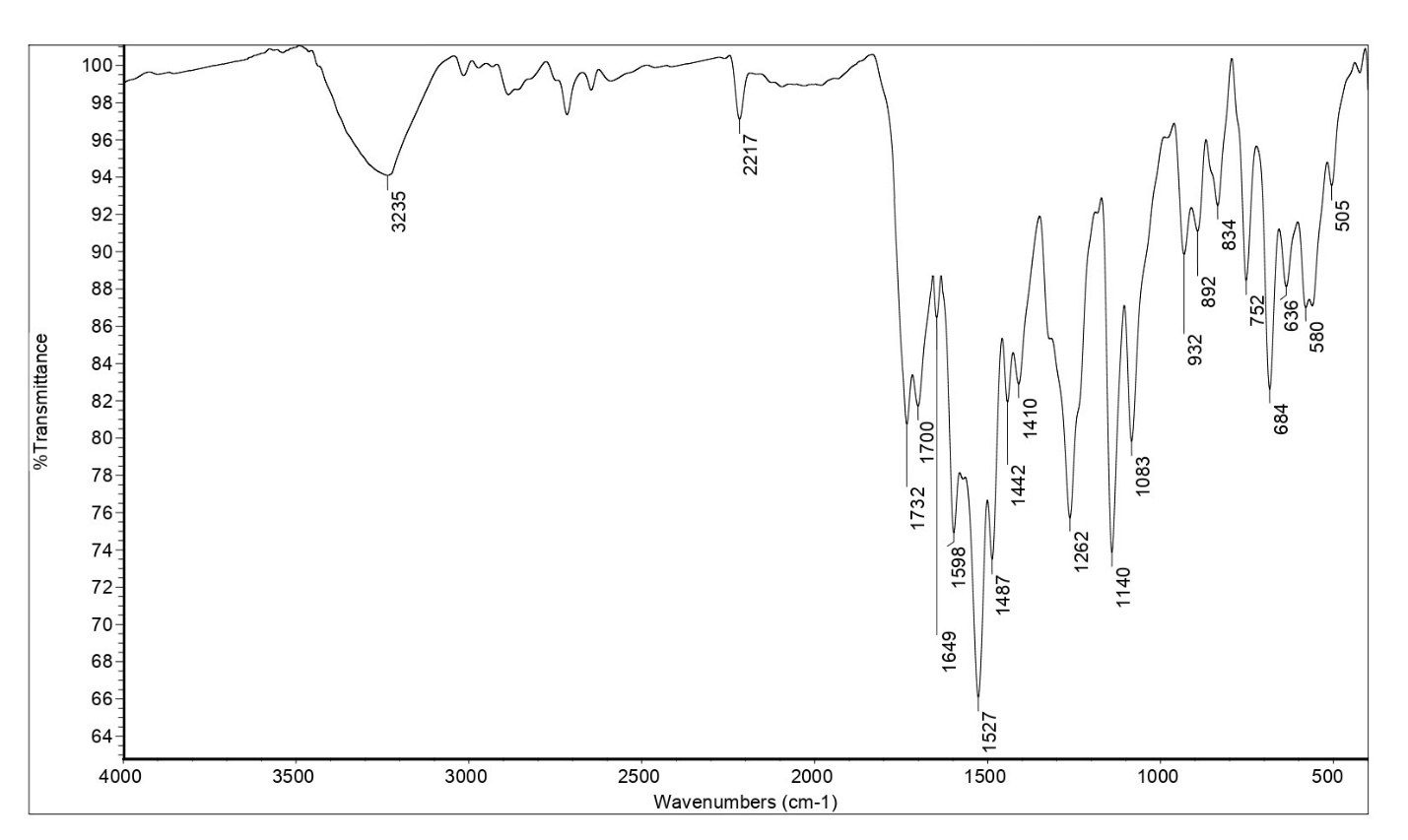
**

**Figure S33.** IR spectrum of compound **18**

IR (KBr): *ν_max_*, cm^-1^: 3235 (NH), 2217 (CN), 1732 (CO), 1700 (CO), 1649 (C=N) functional groups


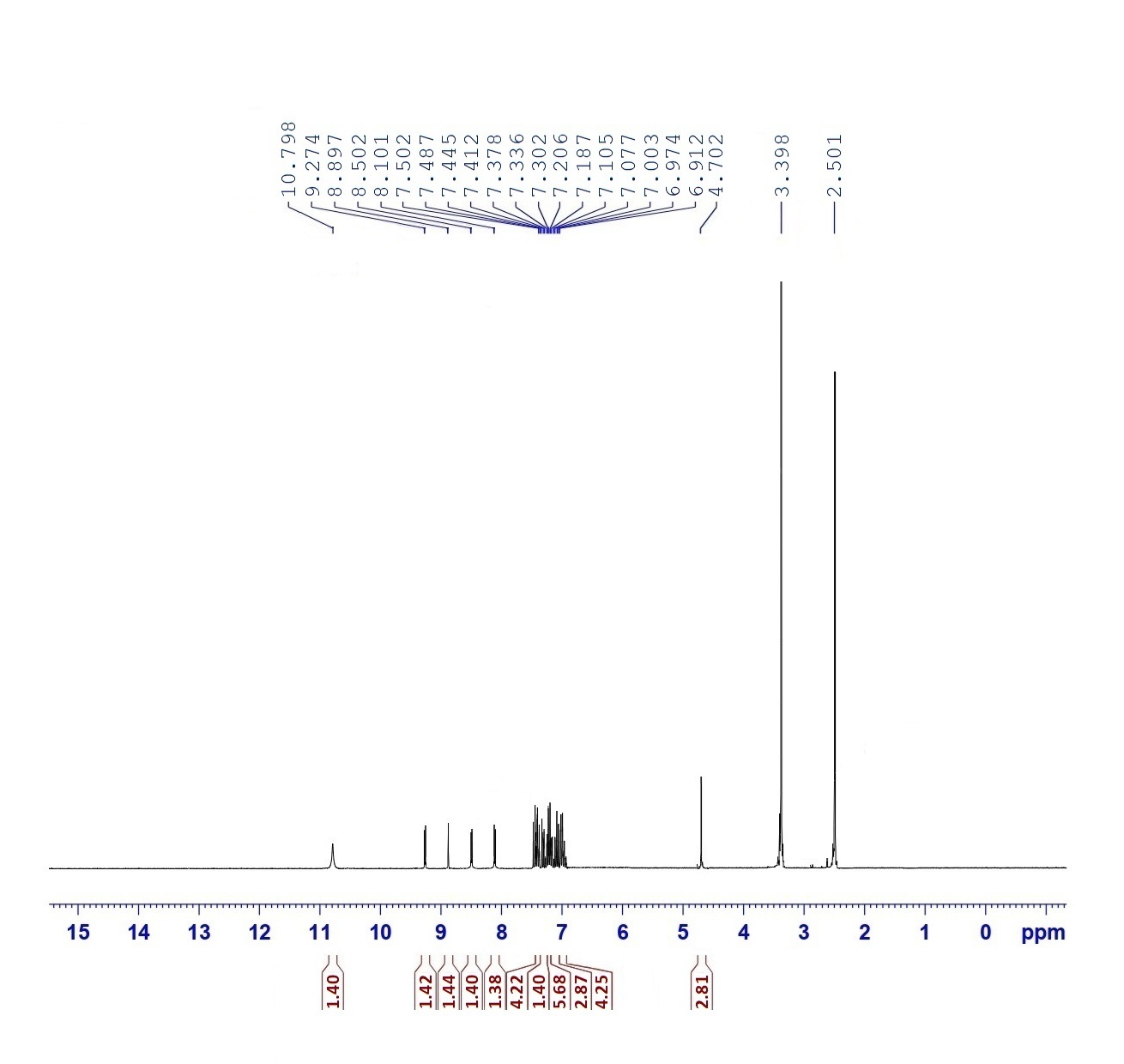


**Figure S34.** ^1^H-NMR of compound **18**

^1^H-NMR (DMSO-*d_6_*) *δ* ppm: 4.70 (s, 2H, CH_2_), 6.91-7.50 (m, 13H, Ar-H + pyridine-C_3_H), 8.10 (d, 1H, Ar-H), 8.50 (d, 1H, pyridine-C_4_H), 8.89 (s, 1H, Ar-H), 9.27 (d, 1H, pyridine-C_2_H), 10.79 (s, 1H, NH)

**
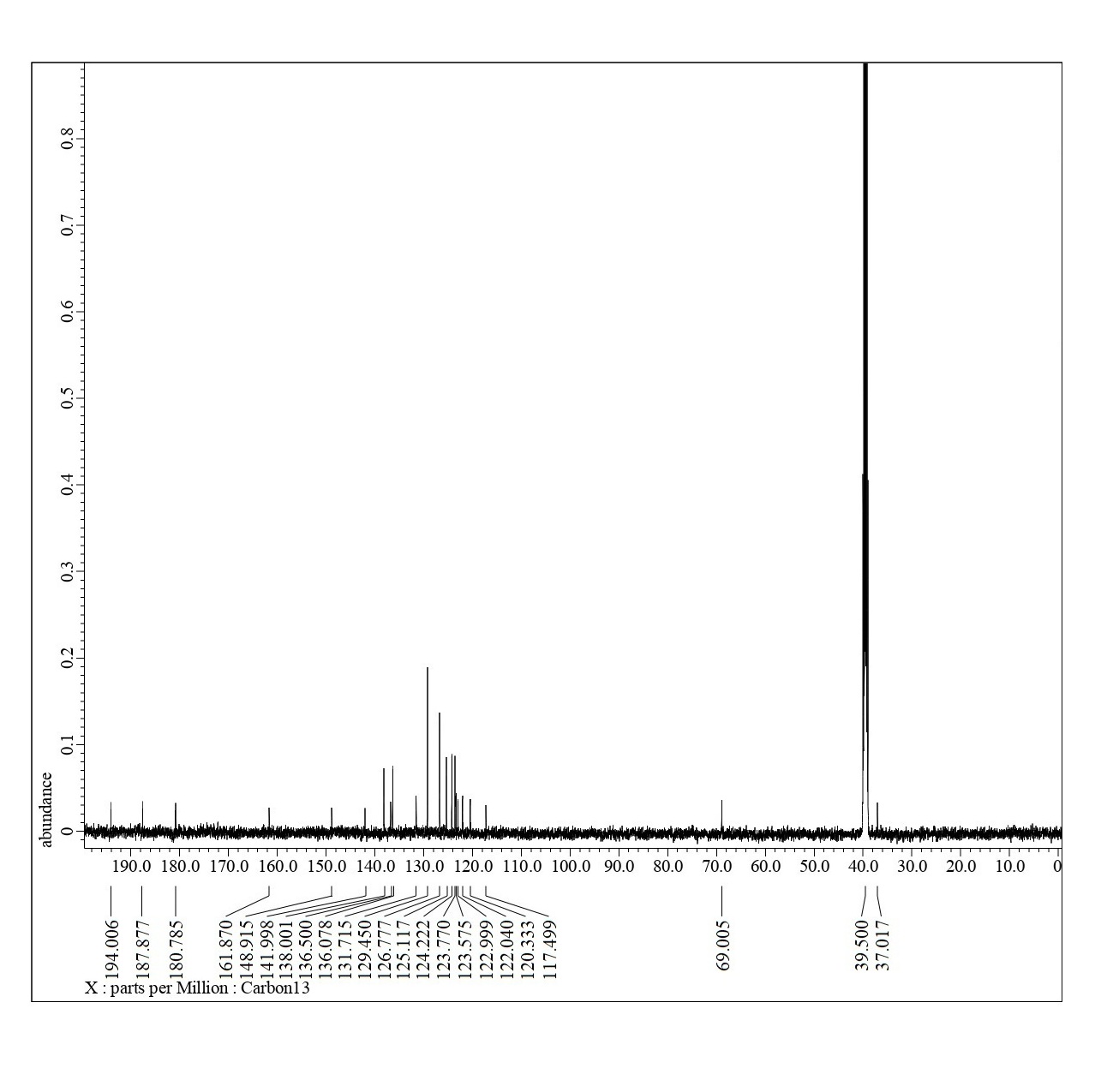
**

**Figure S35.** ^13^C-NMR of Compound **18**

Carbon shifts consistent with naphthoyl-thiophene substituted compound.

**
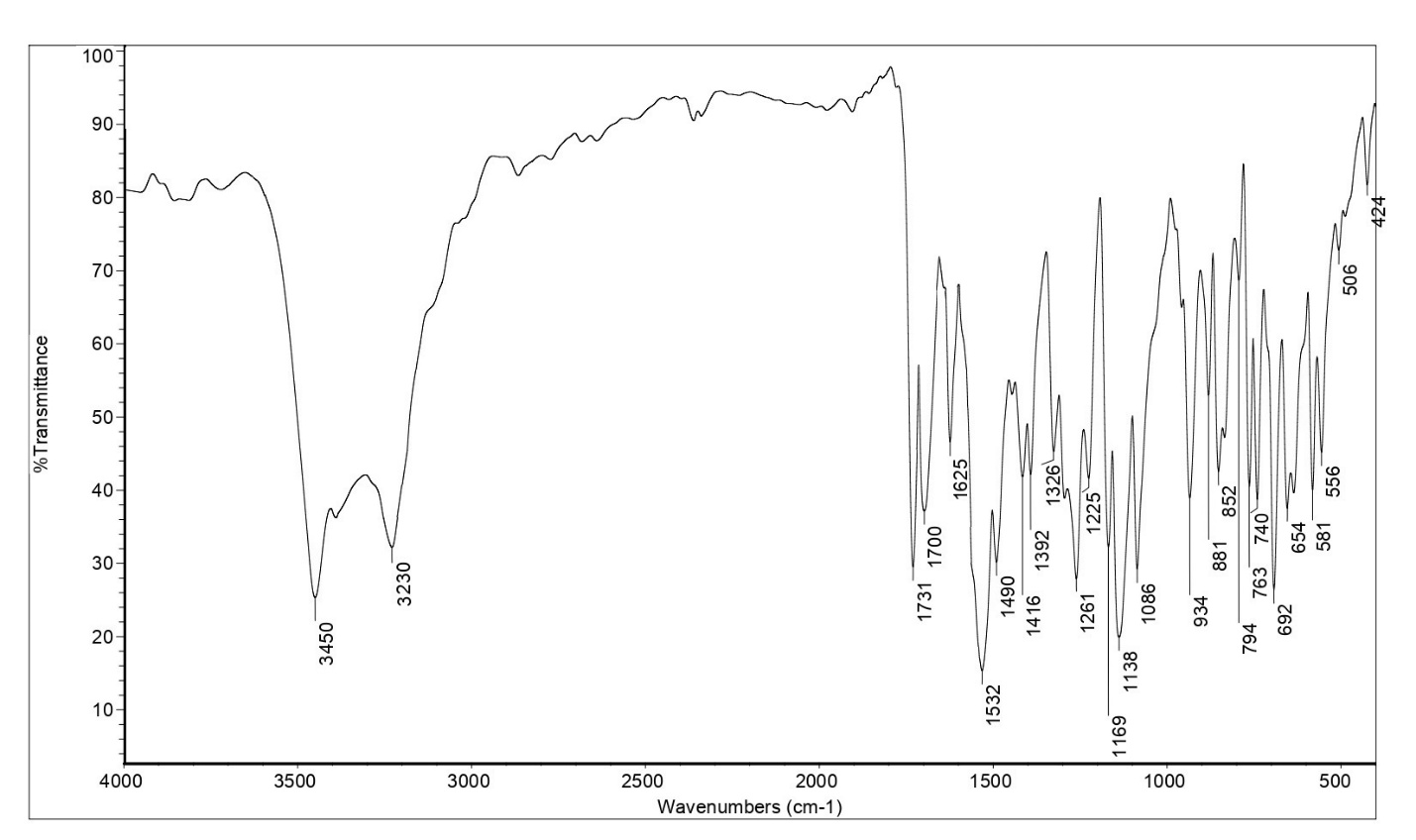
**

**Figure S36.** IR spectrum of compound **19**

IR (KBr): *ν_max_*, cm^-1^: 3450 (NH_2_), 3230 (NH), 1731 (CO), 1700 (CO), 1625 (C=N) functional groups

**
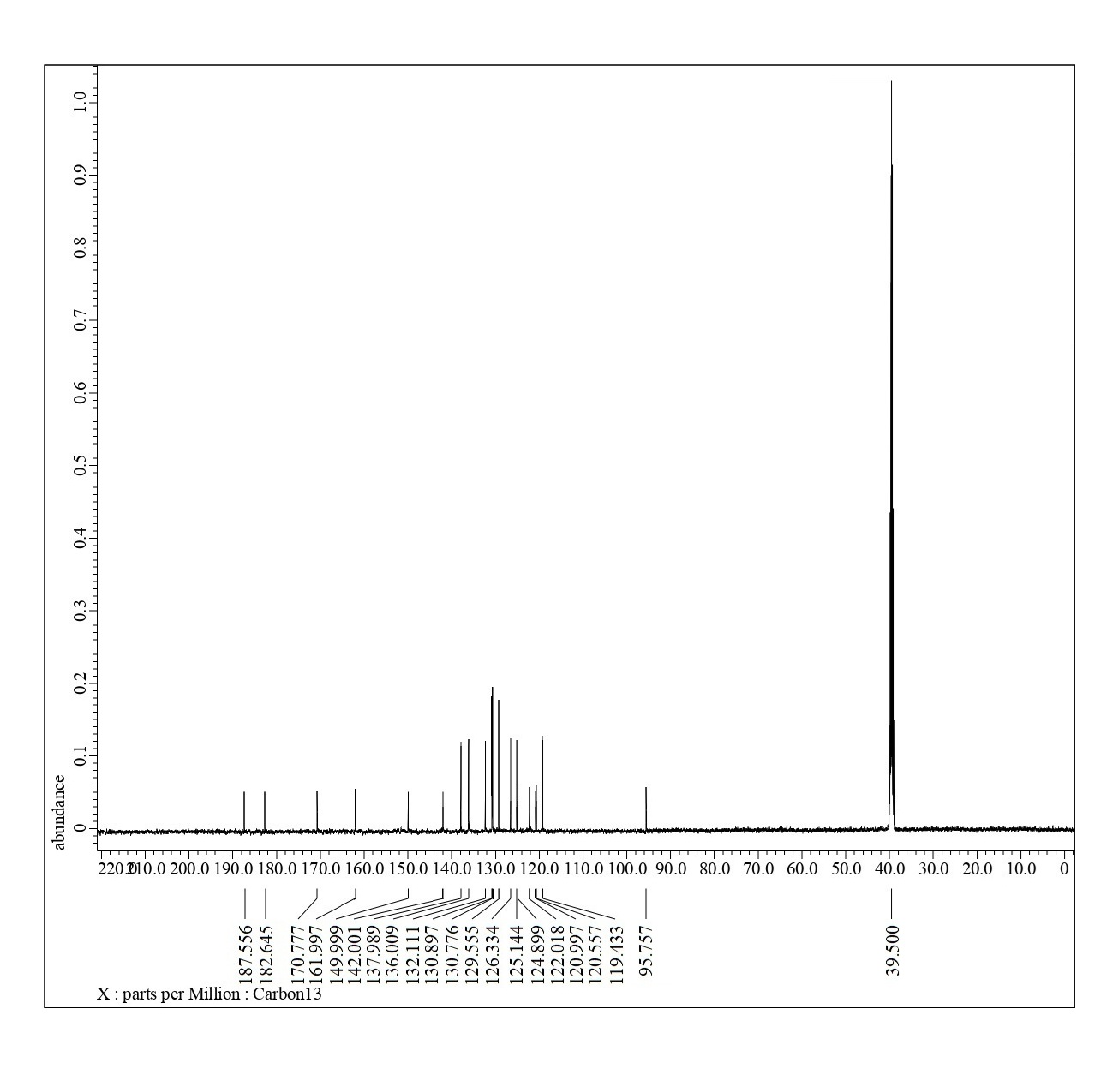
**

**Figure S37.** ^13^C-NMR of Compound **19**

Full signal pattern confirms complex aromatic substitution.

**
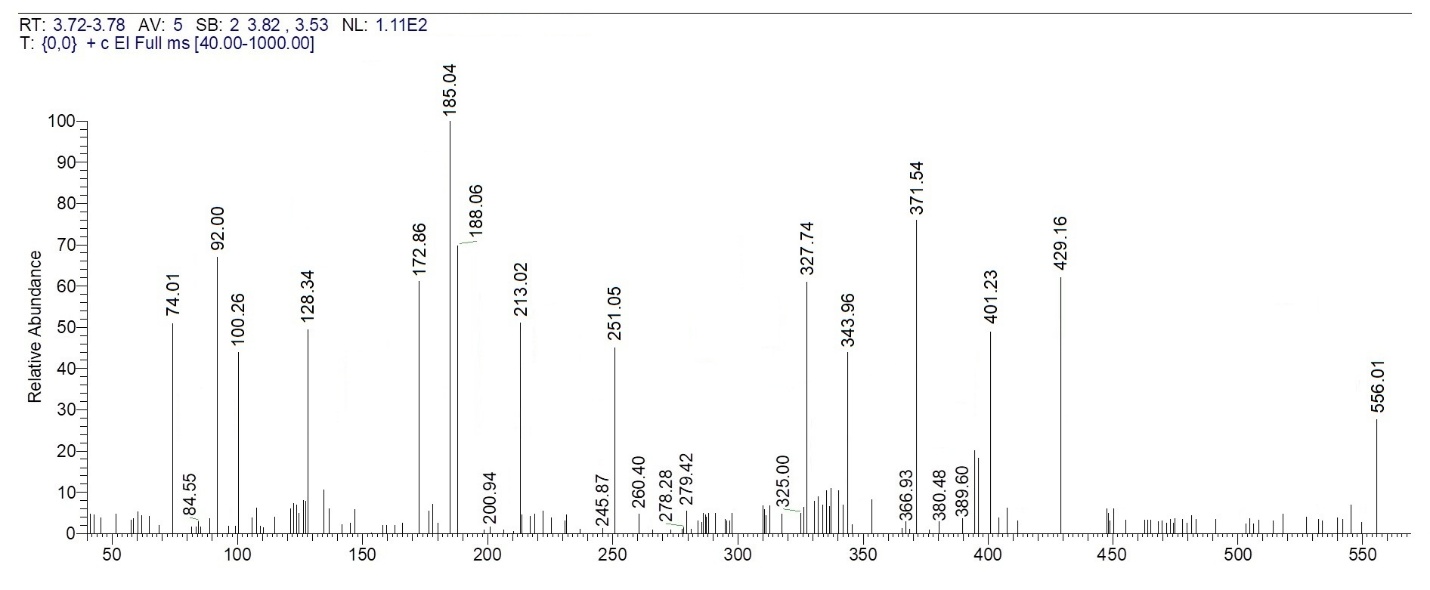
**

**Figure S38.** Mass spectrum of compound **19**

The mass spectrum of compound **19** shows a prominent peak at m/z 556, confirming the molecular weight of the compound.

**
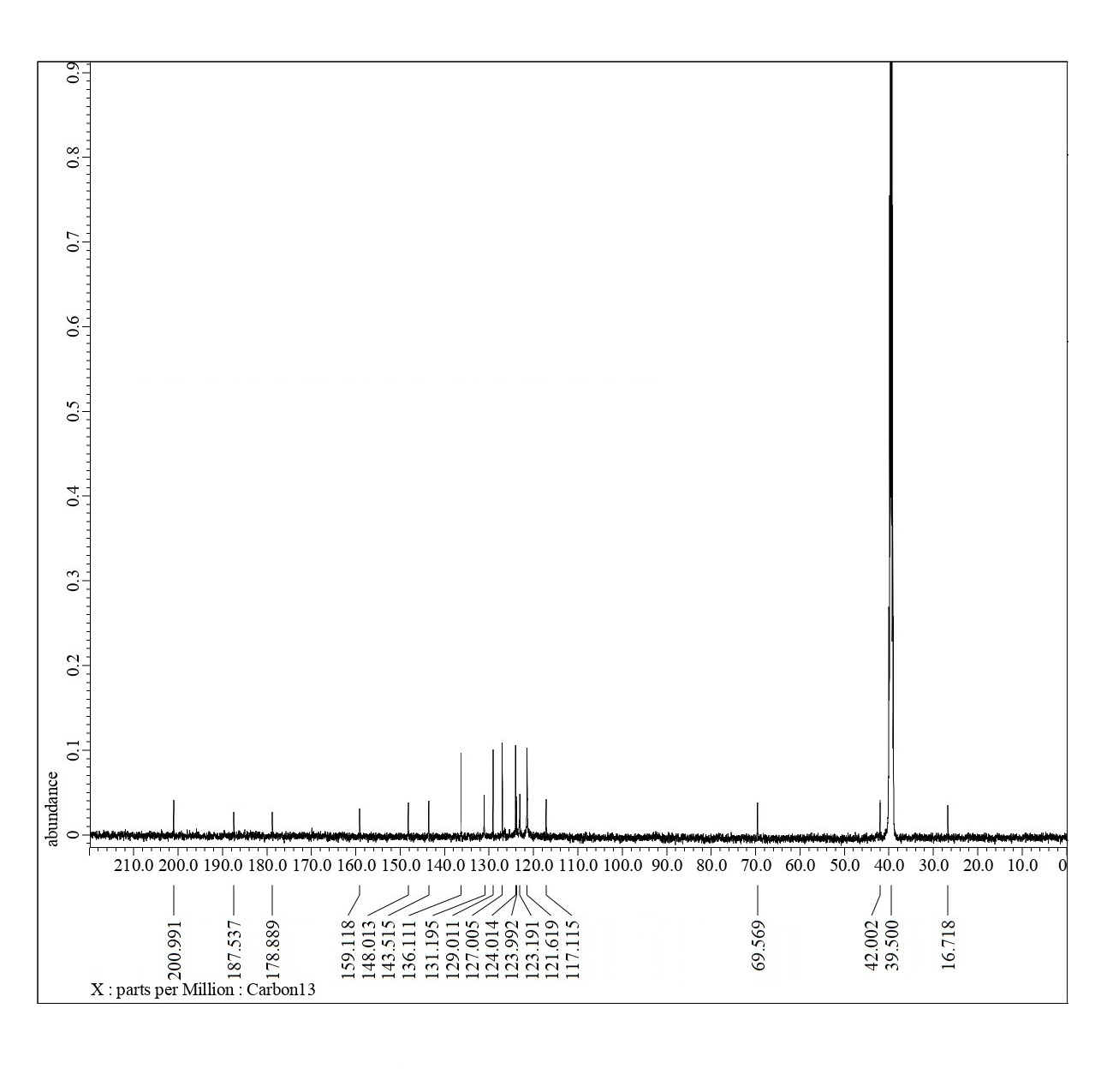
**

**Figure S39.** ^13^C-NMR of Compound **20**

^13^C-NMR spectrum shows chemical shifts consistent with the acyclic structure containing a thiophene moiety and oxopropyl group.

**
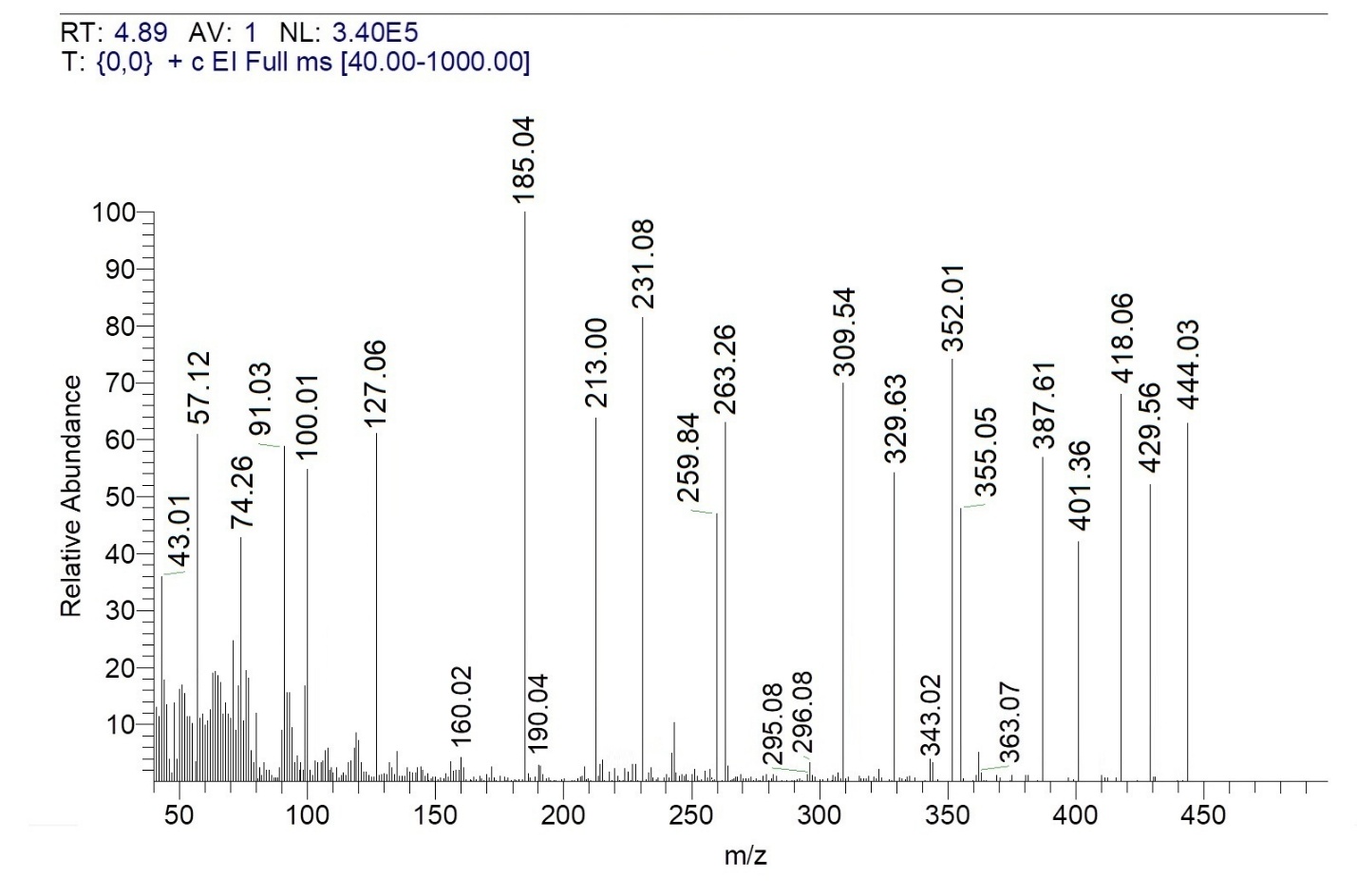
**

**Figure S40.** Mass spectrum of compound **20**

The mass spectrum of compound **20** shows a prominent peak at m/z 444, confirming the molecular weight of the compound.


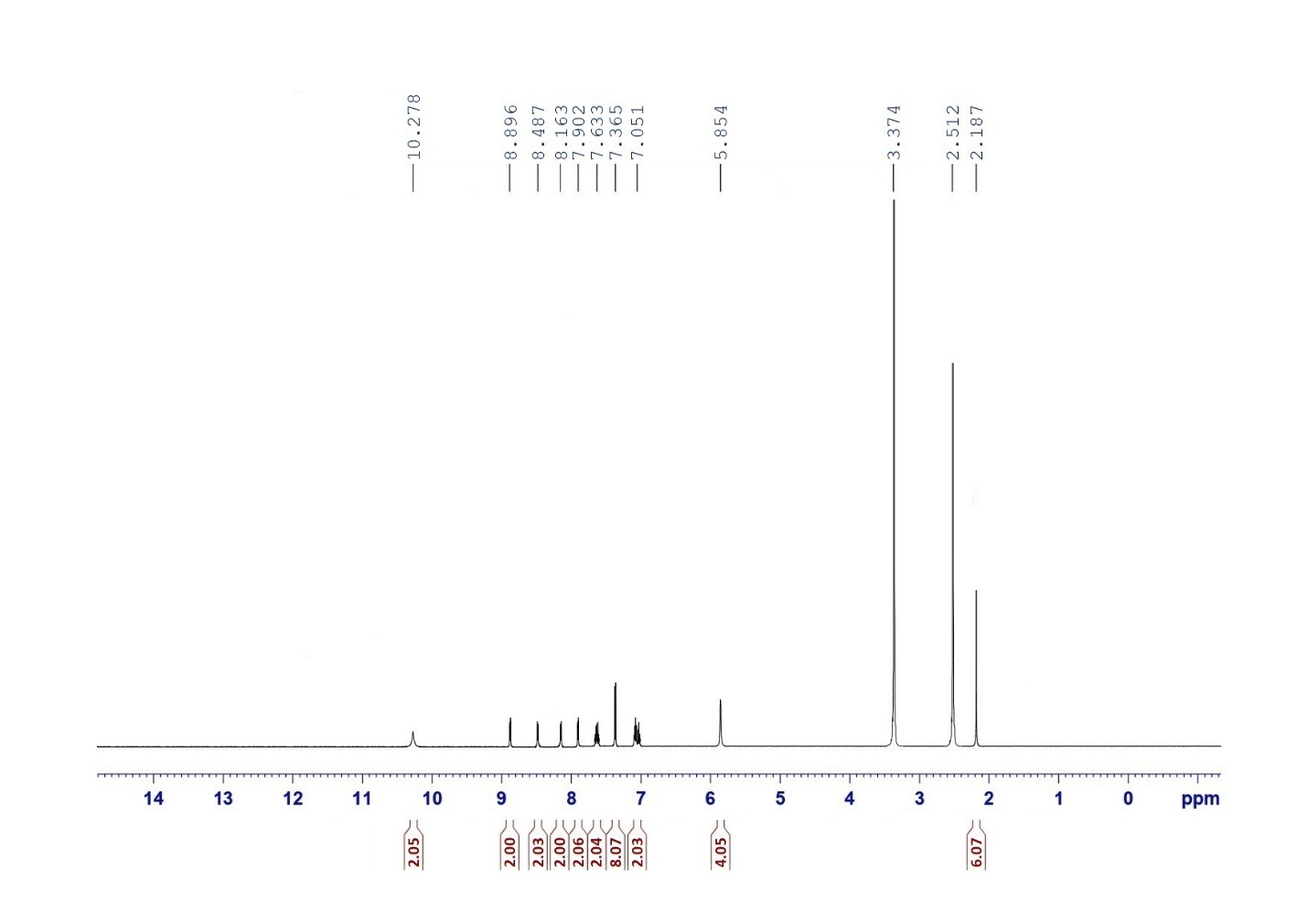


**Figure S41.** ^1^H-NMR of compound **21**

^1^H-NMR (DMSO-*d_6_*) *δ* ppm: 2.18 (s, 3H, CH_3_), 5.85 (s, 2H, NH_2_), 7.05 (m, 1H, Ar-H), 7.36 (d, 4H, Ar-H), 7.63 (m, 1H, pyridine-C_3_H), 7.90 (d, 1H, Ar-H), 8.16 (d, 1H, Ar-H), 8.48 (d, 1H, pyridine-C_4_H), 8.89 (d, 1H, pyridine-C_2_H), 10.27 (s, 1H, NH)

**
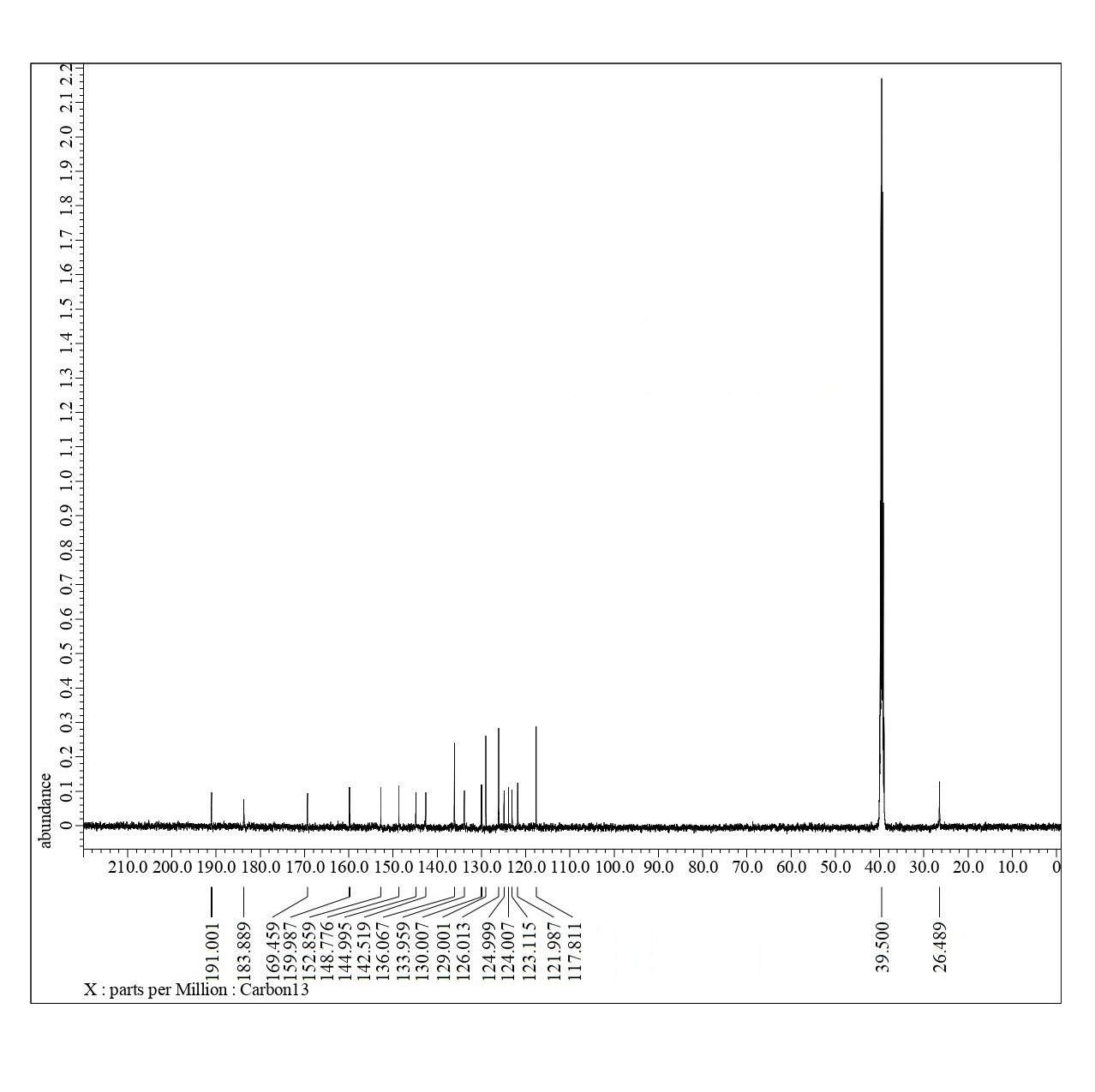
**

**Figure S42.** ^13^C-NMR of Compound **21**

Carbon shifts support the formation of the thiophene-fused system.

**
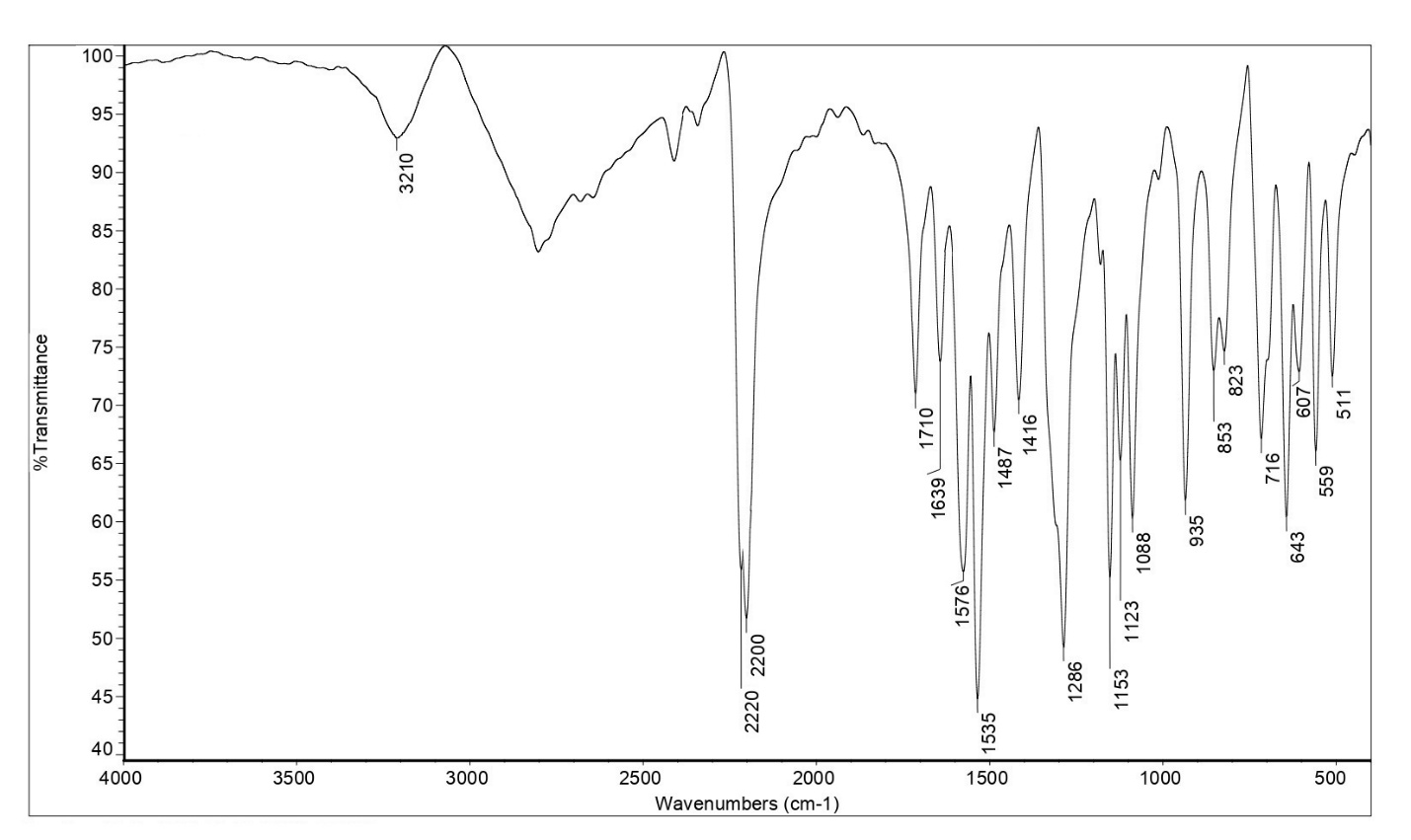
**

**Figure S43.** IR spectrum of compound **22**

IR (KBr): *ν_max_*, cm^-1^: 3210 (NH), 2220 (CN), 2200 (CN), 1710 (CO), 1639 (C=N) functional groups


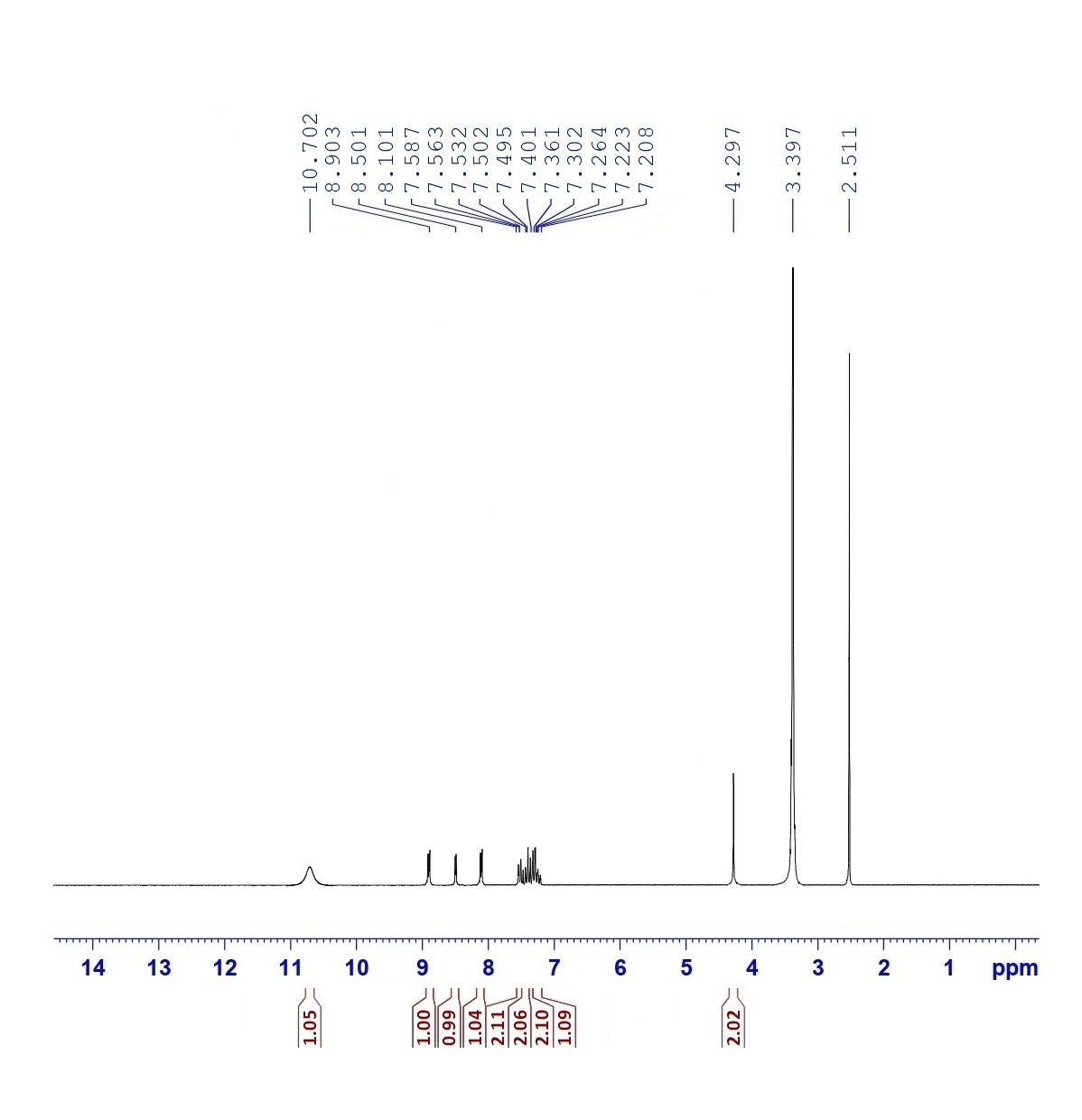


**Figure S44.** ^1^H-NMR of compound **22**

^1^H-NMR (DMSO-*d_6_*) *δ* ppm: 4.29 (s, 2H, CH_2_), 7.20-7.58 (m, 7H, Ar-H + pyridine-C_3_H), 8.10 (d, 1H, Ar-H), 8.50 (d, 1H, pyridine-C_4_H), 8.90 (d, 1H, pyridine-C_2_H), 10.70 (s, 1H, NH)

**
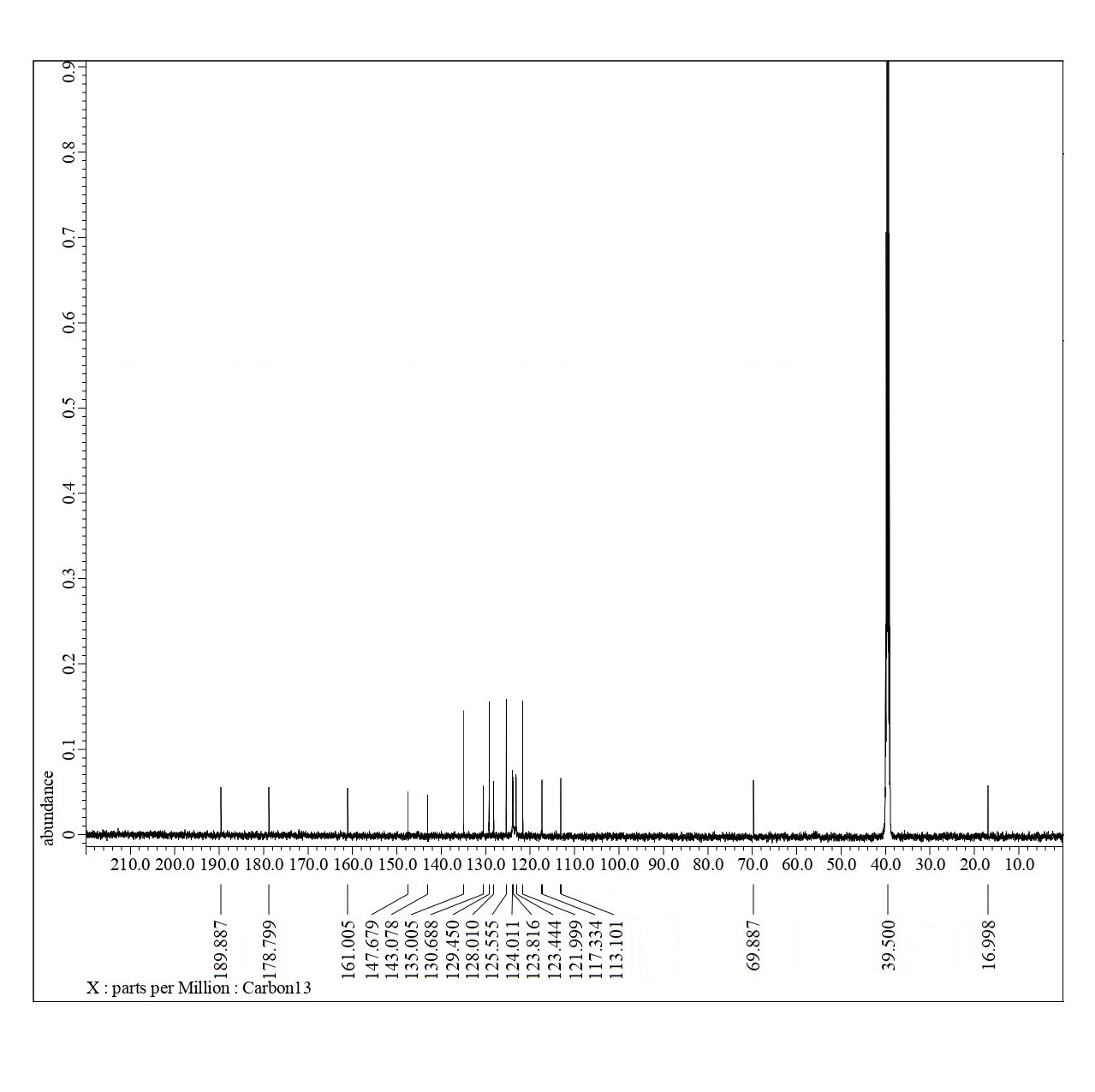
**

**Figure S45.** ^13^C-NMR of Compound **22**

Supports the presence of the phenylamino, cyano, and thiazoloquinoline moieties.

**
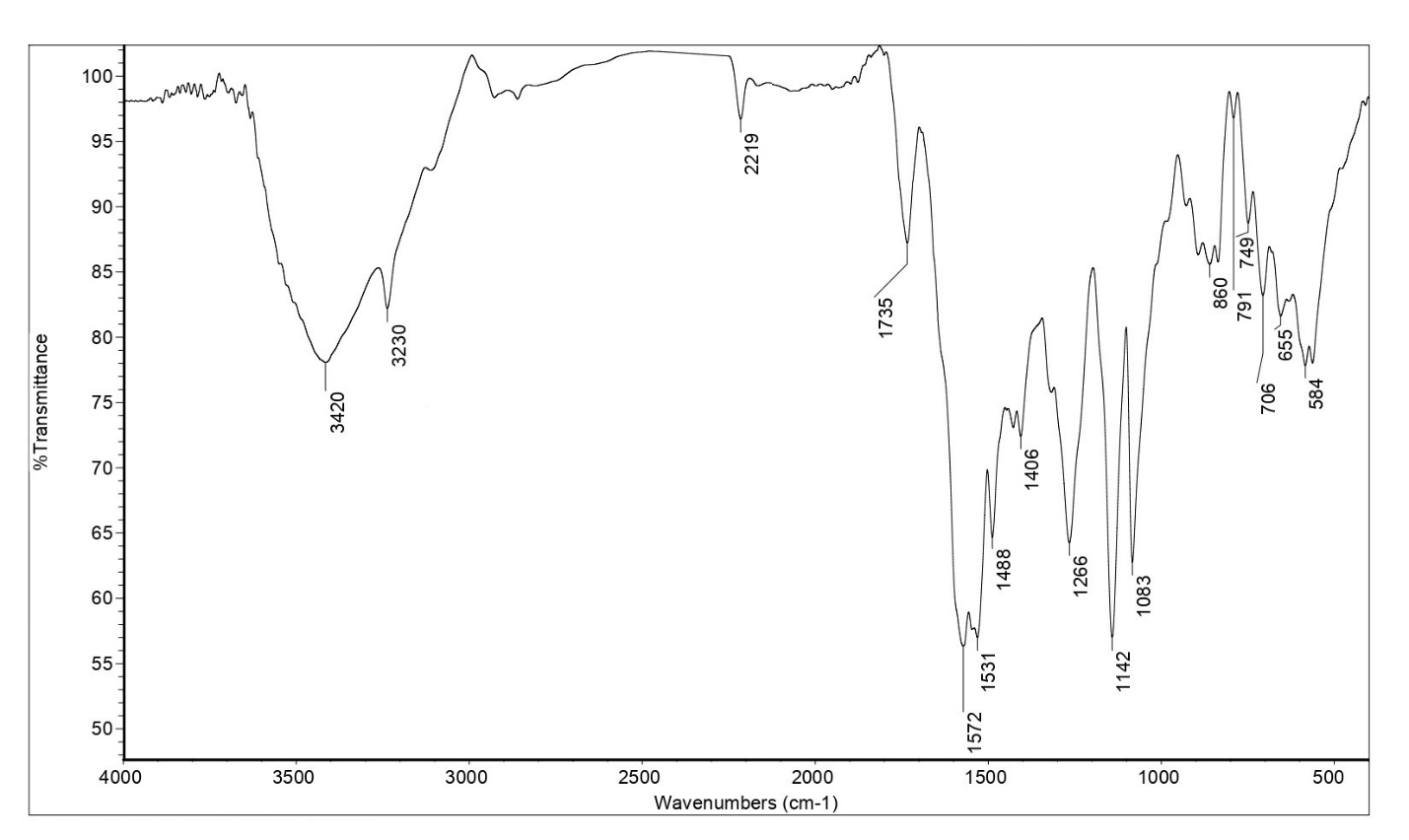
**

**Figure S46.** IR spectrum of compound **23**

IR (KBr): *ν_max_*, cm^-1^: 3420 (NH_2_), 3230 (NH), 2219 (CN), 1735 (CO) functional groups

**
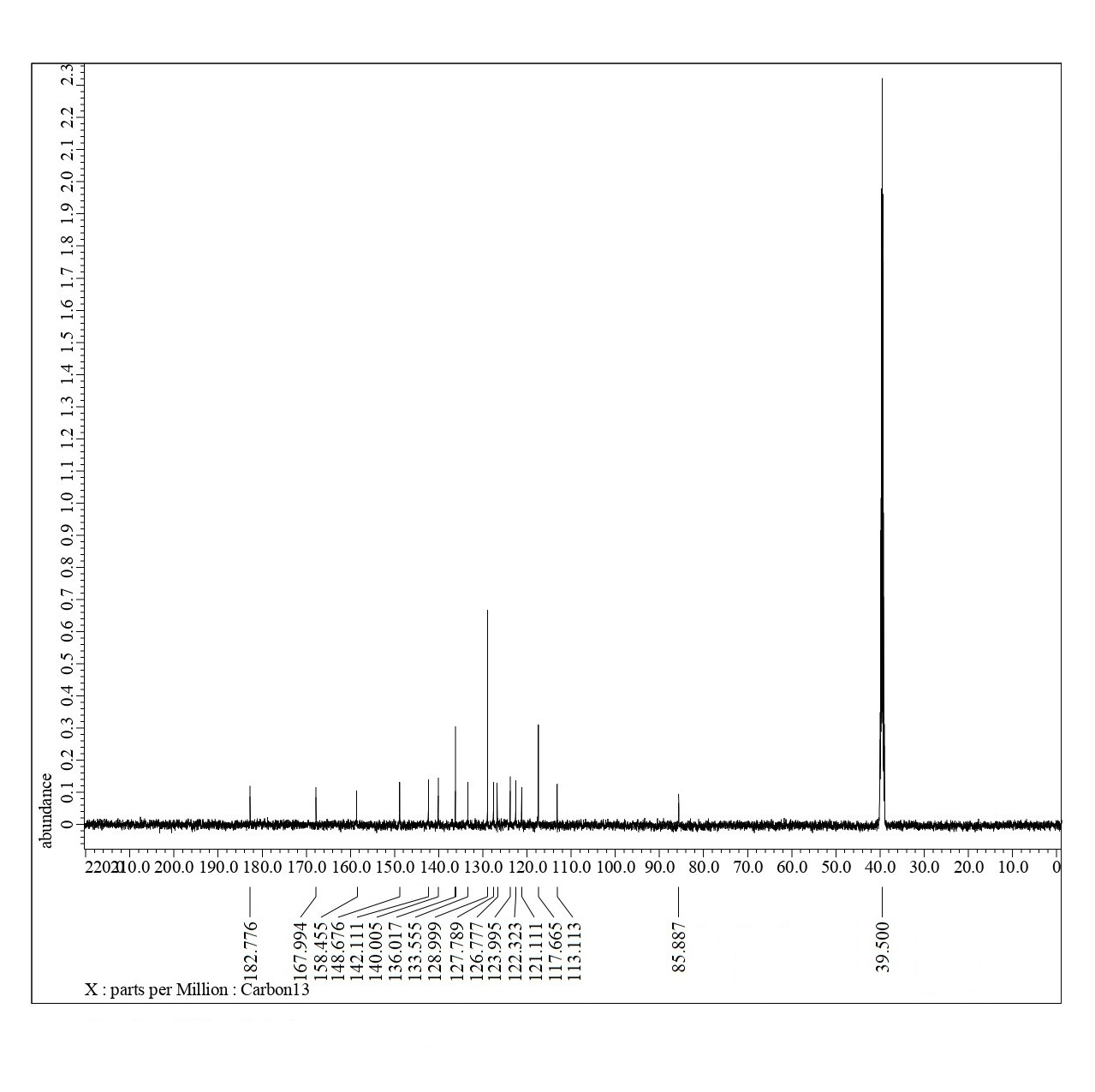
**

**Figure S47.** ^13^C-NMR of Compound **23**

Signals consistent with thiophene–quinoline–phenylamino hybrid structure.

**
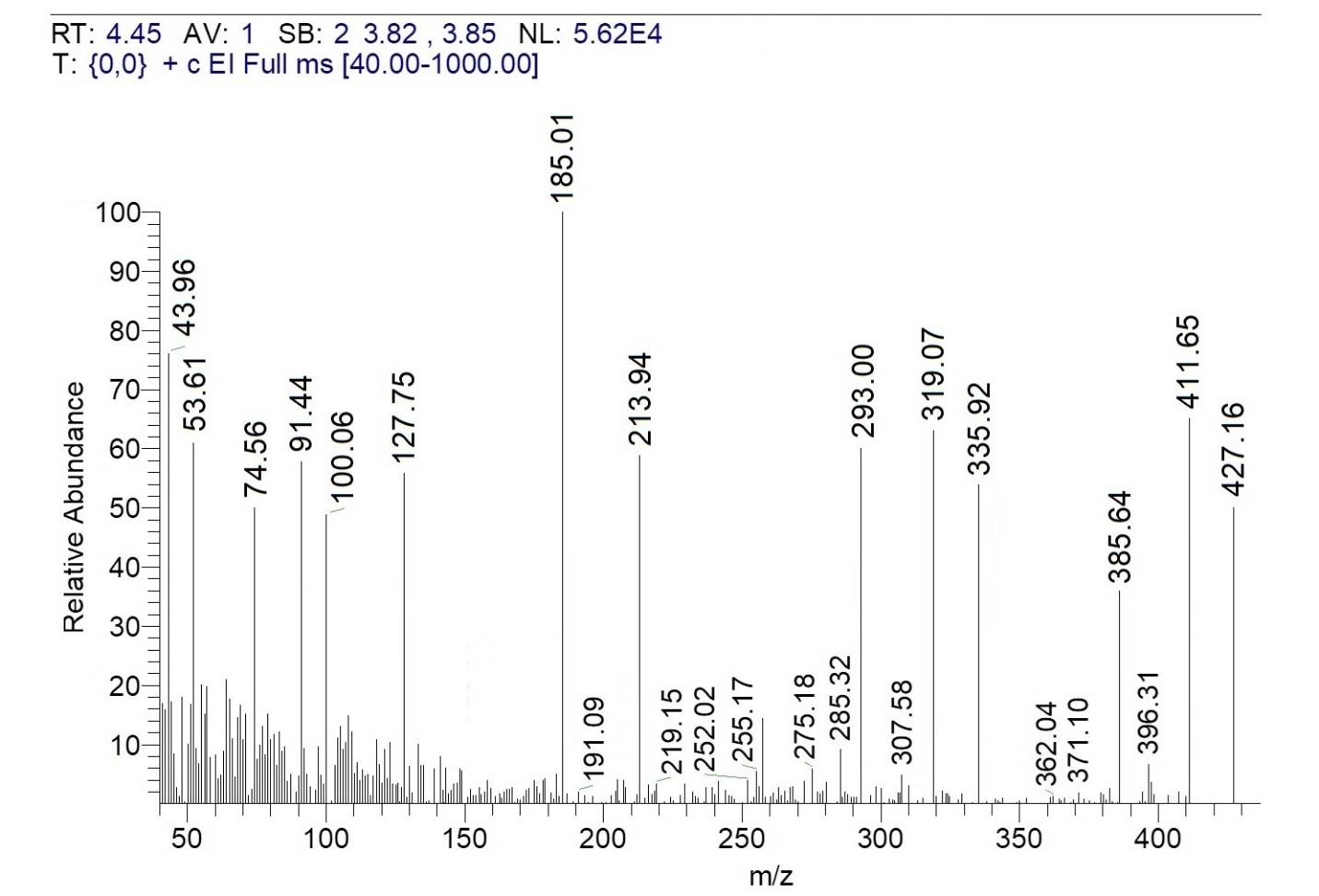
**

**Figure S48.** Mass spectrum of compound **23**

The mass spectrum of compound **23** shows a prominent peak at m/z 427, confirming the molecular weight of the compound.

**
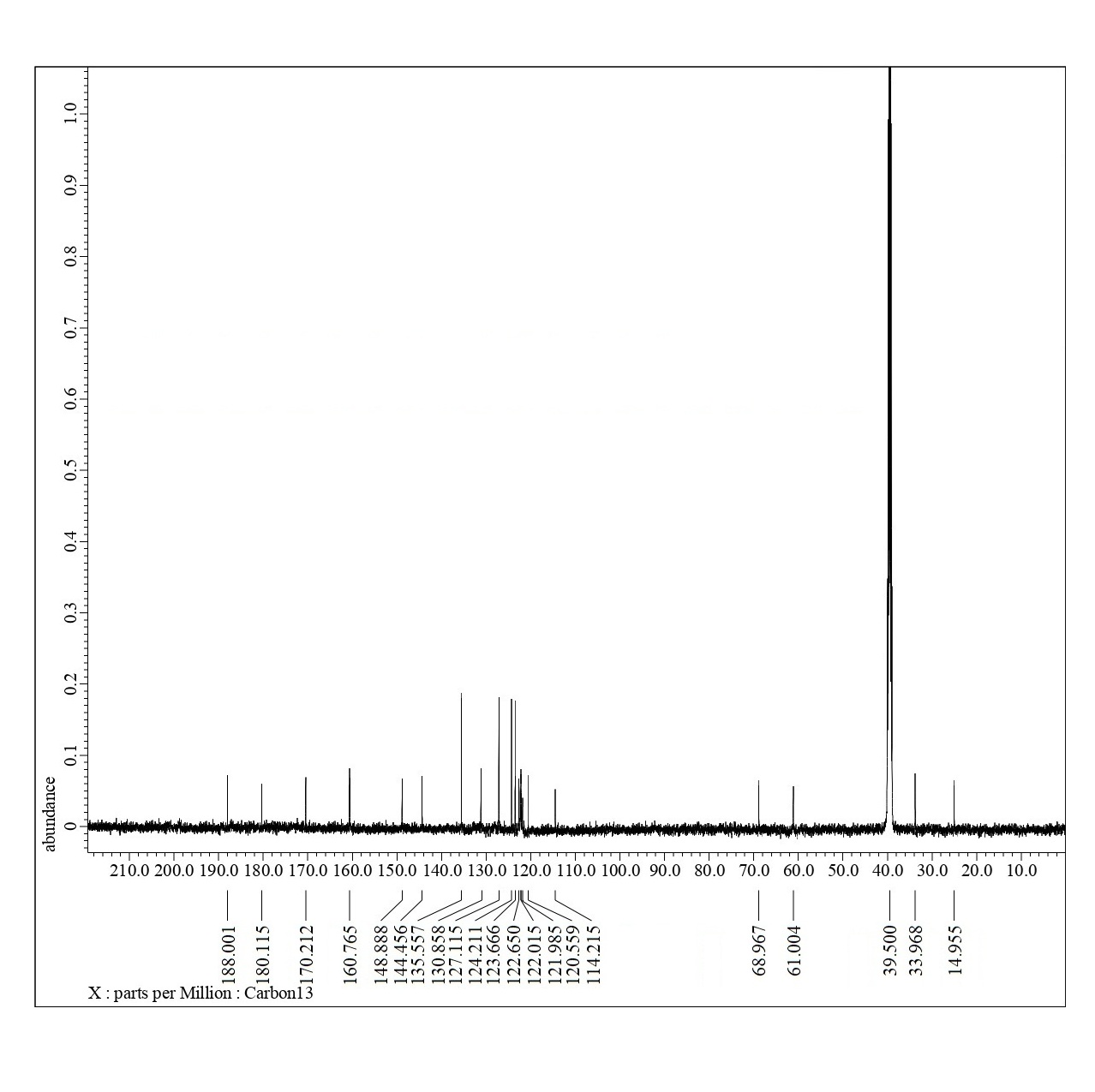
**

**Figure S49.** ^13^C-NMR of Compound **24**

Shows shifts for ethyl ester, CN, aromatic, and carbonyl carbons.

**
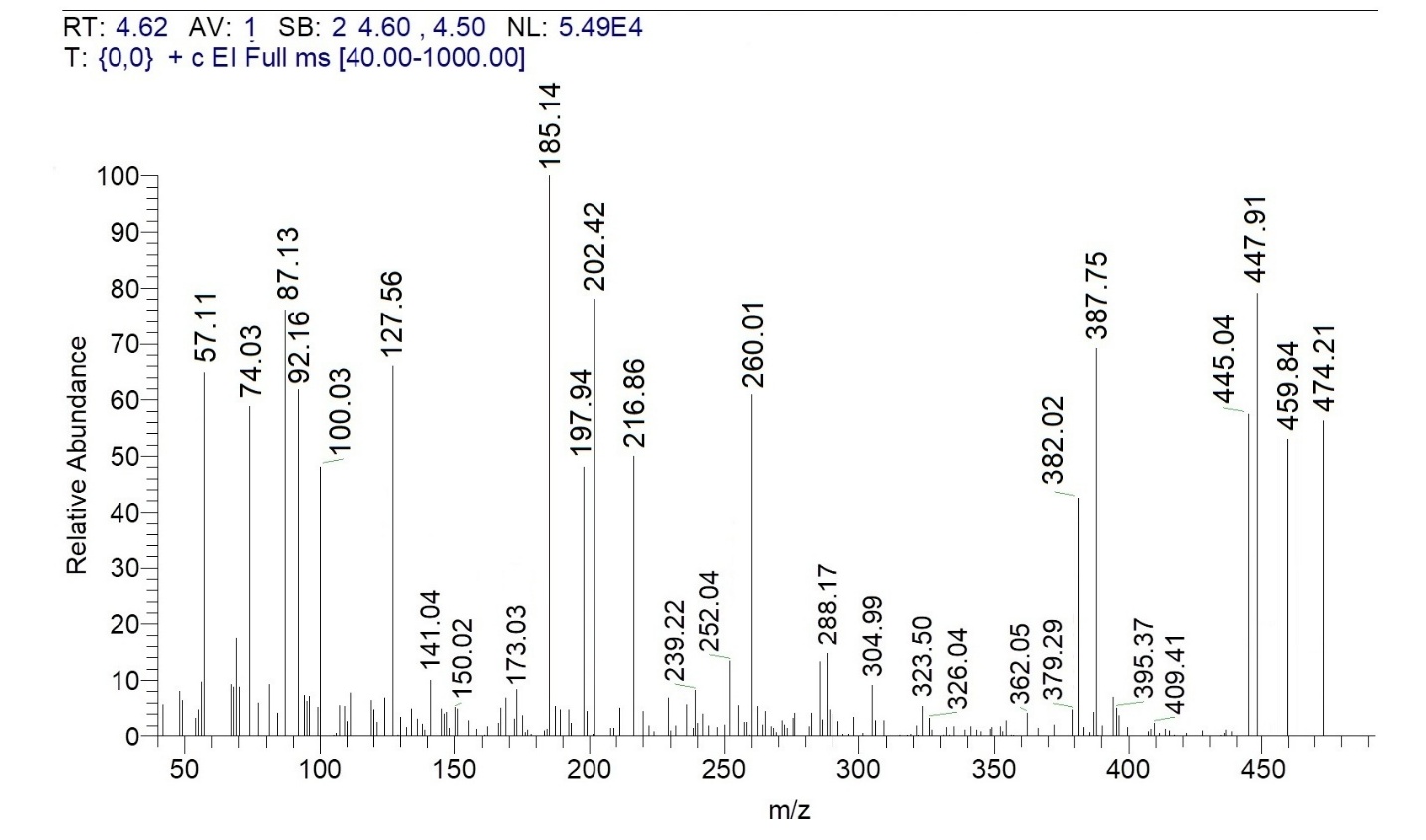
**

**Figure S50.** Mass spectrum of compound **24**

The mass spectrum of compound **24** shows a prominent peak at m/z 474, confirming the molecular weight of the compound.


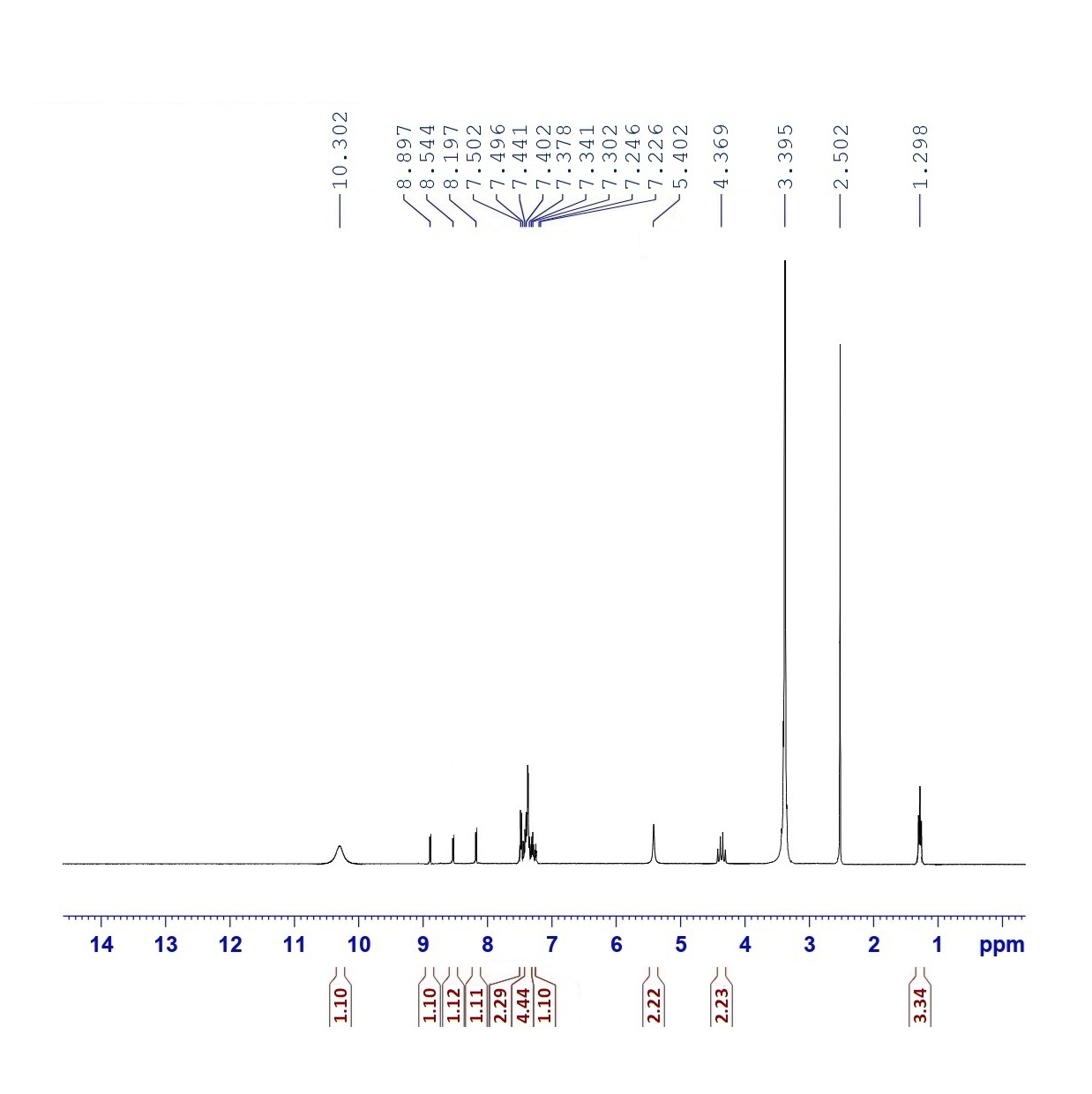


**Figure S51.** ^1^H-NMR of compound **25**

^1^H-NMR (DMSO-*d_6_*) *δ* ppm: 1.29 (t, 3H, CH_3_), 4.36 (q, 2H, CH_2_), 5.40 (s, 2H, NH_2_), 7.22-7.50 (m, 7H, Ar-H + pyridine-C_3_H), 8.19 (d, 1H, Ar-H), 8.54 (d, 1H, pyridine-C_4_H), 8.89 (d, 1H, pyridine-C_2_H), 10.30 (s, 1H, NH)


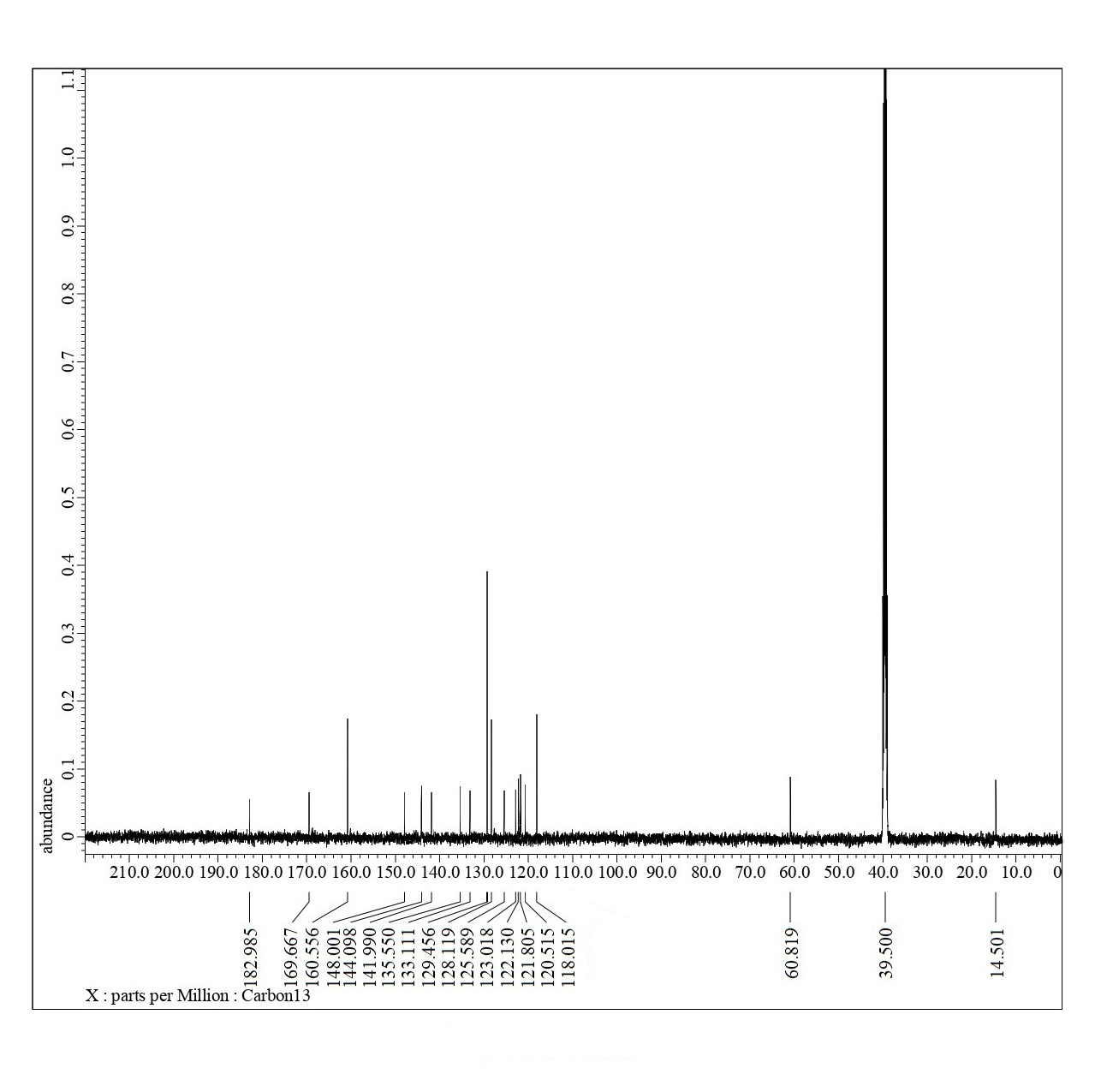


**Figure S52.** ^13^C-NMR of Compound **25**

Confirmatory peaks for ester, CN, thiophene, and thiazoloquinoline moieties.
